# Rational design of a Kappa opioid receptor peptide agonist with attenuated β-arrestin signaling

**DOI:** 10.1038/s41467-026-71455-3

**Published:** 2026-04-14

**Authors:** Huanhuan Zhang, Ruolan Wang, Pan Shi, Gaoming Wang, Qingjun Zhu, Xinheng He, Youwei Xu, Qingning Yuan, Wen Hu, Kai Wu, Yong Zheng, Li Zhou, Jun Liang, Pei Lv, Ziyan Xu, Fan Yang, Yingbin Liu, Youwen Zhuang, H. Eric Xu, Yue Wang, Changlin Tian

**Affiliations:** 1 Department of Endocrinology, the First Affiliated Hospital of USTC, School of Life Sciences, Division of Life Sciences and Medicine, University of Science and Technology of China, Anhui, P. R. China; 2 Anhui Engineering Laboratory of Peptide Drug, University of Science and Technology of China, Anhui, China; 3 The State Key Laboratory of Drug Research, Center for Structure and Function of Drug Targets, Shanghai Institute of Materia Medica, Chinese Academy of Sciences, Shanghai, China; 4 University of Chinese Academy of Sciences, Beijing, China; 5 Department of Biliary-Pancreatic Surgery, Renji Hospital Affiliated to Shanghai Jiao Tong University School of Medicine, Shanghai, China; 6 High Magnetic Field Laboratory, Hefei Institute of Physical Science, Chinese Academy of Sciences, Anhui, China; 7 The Shanghai Advanced Electron Microscope Center, Shanghai Institute of Materia Medica, Chinese Academy of Sciences, Shanghai, China; 8 School of Chinese Materia Medica, Nanjing University of Chinese Medicine, Nanjing, China; 9 School of Biomedical Engineering, Suzhou Institute for Advanced Research, University of Science and Technology of China, Suzhou, Jiangsu China; 10 Department of Pharmaceutical and Artificial-Intelligence Sciences, School of Medicine, Shanghai Jiao Tong University, Shanghai, China; 11 Beijing Life Science Academy, Beijing, China; 12 School of Chemistry and Chemical Engineering, ZhangJiang Institute for Advanced Study, Shanghai Jiao Tong University, Shanghai, China

**Keywords:** G protein-coupled receptors, Cryoelectron microscopy, Drug discovery

## Abstract

Difelikefalin is an FDA-approved κ-opioid receptor (KOR) peptide agonist used to treat chronic pruritus. However, as a balanced agonist that activates both G protein and β-arrestin pathways, difelikefalin remains associated with undesirable side effects linked to β-arrestin signaling. Here, we report the cryo-EM structure of the difelikefalin-KOR-Gi complex, identifying Y320^7.43^ as a key residue that is critical for signaling bias. Guided by this structural insight, we engineer beta01, a β-amino acid-substituted analog with potent G protein activation but minimal β-arrestin recruitment. In mouse models, beta01 retains robust antinociceptive and antipruritic efficacy while significantly reducing sedation and anxiety-like behaviors. Structural, molecular dynamics simulations and 2D ^13^C-Met NMR analyses further reveal beta01 stabilizes a unique KOR conformation with an expanded intracellular cavity that disfavors β-arrestin binding. This work establishes a rational structure-based framework for designing safer and more effective GPCR-targeted therapeutics.

## Abstract (web summary)

Difelikefalin is an FDA-approved KOR-targeted drug for chronic pruritus yet exhibits side effects. Here, researchers solve cryo-EM structure of difelikefalin KOR-Gi, identifying Y320^7.43^ as a key bias residue. Substituting D-Phe1 with β-phenylalanine develops beta01 which retains analgesic/antipruritic efficacy via a unique receptor conformation that reduces adverse effects.

## Introduction

Opioids play a crucial role in managing moderate to severe pain by engaging class A G protein-coupled receptors (GPCRs), specifically the μ-, δ-, and κ-opioid receptors (MOR, DOR and KOR) as well as the nonclassical nociceptin opioid receptor (NOPR)^1,2^. Although MOR-directed ligands dominate in current clinical practice, their utility is hampered by dose-limiting adverse events, including constipation, respiratory depression, and addiction liability^3–5^. In contrast, KOR agonists have emerged as promising therapeutic alternatives because of their low risk of addiction and minimal gastrointestinal and respiratory side effects^6–9^. Consequently, KOR has emerged as an attractive target for developing pharmacotherapies to address various conditions, including pain, pruritus, epilepsy, multiple sclerosis, inflammatory diseases, gastrointestinal disorders and ischemia^8,10–12^.

**Fig. 1:**
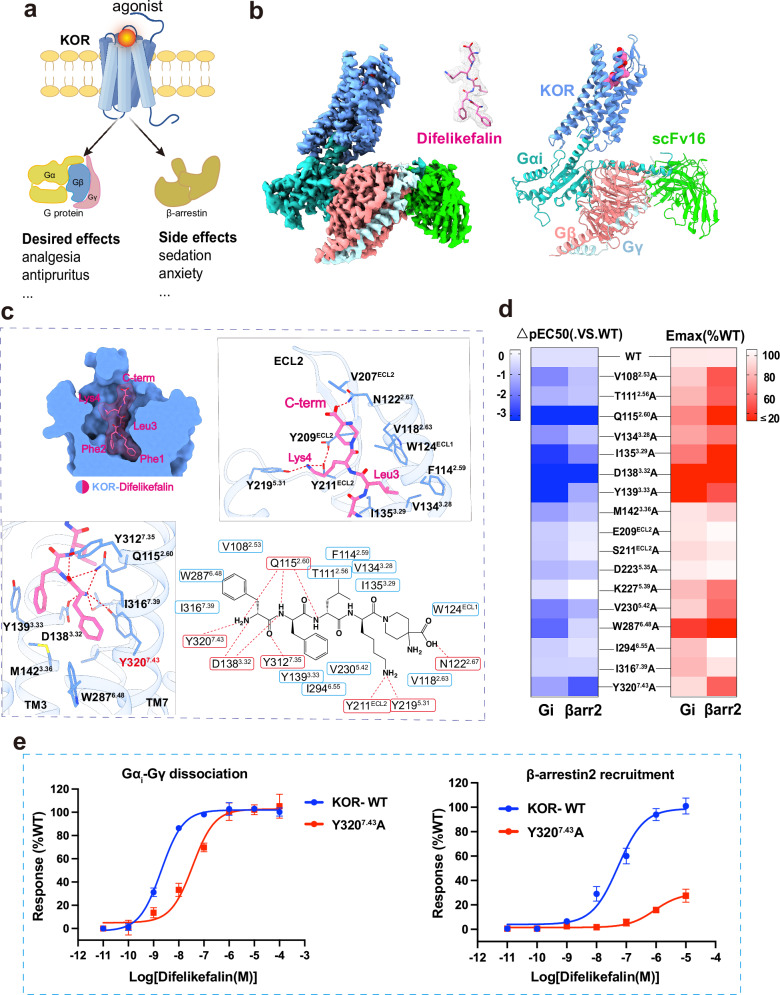
Molecular recognition of difelikefalin by KOR. **a** Agonist induced KOR signaling and potential pharmacological effects. KOR activation via the G-protein pathway is associated with therapeutic benefits, whereas the β-arrestin pathway is linked to adverse side effects. **b** The cryo-EM density maps and molecular model of difelikefalin-KOR-Gi complex. Colored by subunit (difelikefalin in hot pink, KOR in cornflower blue, Gα_i_ in light sea green, Gβ in light coral, Gγ in powder blue, scFv16 in lime). **c** The binding pocket of difelikefalin in KOR. Detailed interactions between residues in difelikefalin and KOR are shown in figures and the polar interactions are shown as red dashed lines. **d** Mutational effects on G protein and β-arrestin2 signaling of KOR induced by difelikefalin, assessed using a Gα_i1–γ2_ dissociation assay and β-arrestin2 NanoBiT assay, respectively. In the heatmap, darker colors indicate more severe functional impairment of the mutants. Data shown are means ± S.E.M. from three independent experiments performed in technical duplicates. Source data are provided as a Source Data file. **e** Effects of Y320^7.43^A of KOR on G-protein signaling (left) and β-arrestin2 recruitment (right) in response to difelikefalin. This result showed that the Y320 residue is important for arrestin signaling transduction. Data shown are means ± S.E.M. from three independent experiments performed in technical duplicates. Source data are provided as a Source Data file.

Recent efforts via various strategies have been applied to develop opioid drugs with improved safety profiles, with a particular focus on G protein-biased opioid agonists^13,14^. Early studies in β-arrestin2-deficient mice treated with morphine revealed attenuated constipation and respiratory depression but maintained analgesic effects^15–17^. This finding prompted the hypothesis that β-arrestin signaling might contribute to the side effects of MOR agonists, whereas the G protein pathway may mediate analgesia. However, subsequent studies have challenged this hypothesis, demonstrating that side effects can persist in the absence of β-arrestin2^18^. Despite these discrepancies, the concept of G protein bias remains promising for KOR-targeted therapies^19–21^. Accumulating evidence suggests that the KOR-mediated antinociceptive and antipruritic effects are driven primarily by G protein signaling, whereas β-arrestin signaling contributes to side effects, such as sedation and anxiety (Fig. 1a)^21,22^. Consistent with this model, several G protein-biased KOR agonists, such as nalfurafine, RB-64, triazole 1.1 and HS666, have exhibited robust analgesic and anti-itching effects with reduced side effects of sedation, anxiety or dysphoria^21,23–27^. Overall, designing KOR agonists with G protein-biased signaling and attenuated β-arrestin signaling is a rational strategy for developing safer opioid alternatives.

Difelikefalin (sequence: D-Phe-D-Phe-D-Leu-D-Lys-[γ-(4-N-piperidinyl) amino carboxylic acid] acetate salt; trade name: Korsuva) is a selective KOR peptide agonist approved by the US Food and Drug Administration in 2021 for treating moderate to severe pruritus associated with chronic kidney disease (CKD-aP) in hemodialysis patients^28–33^. Despite its analgesic and antipruritic efficacy, difelikefalin has frequently been reported to have several adverse side effects, including dizziness, falls, mental depression, anxiety, and difficulty walking^30,33,34^. Therefore, structural optimization of difelikefalin-based KOR peptide agonists is very important to improve their safety profiles. Given the clinical importance of difelikefalin as a selective KOR peptide agonist, its structural mechanism of selective KOR activation and biased signal transduction must be characterized, which is also important for the rational design of KOR peptide agonists.

In this work, we apply single-particle cryo-EM to determine the structure of the difelikefalin-bound KOR–Gi complex. Structural analysis reveals that the Y320^7.43^ residue in KOR interacts with the first residue of difelikefalin, while serial mutational analysis verify that the Y320^7.43^ residue is critical for modulating the balance between KOR-mediated G protein signaling and β-arrestin signaling. To explore structure–activity relationships, we synthesize and test over 50 difelikefalin-derived peptides for their effects on KOR signaling. Among them, the peptide agonist beta01, which features a single substitution at the N-terminal residue-replacing the native α-amino acid Phe with a β-amino acid analog-exhibits marked G protein bias, with negligible β-arrestin recruitment. This subtle backbone modification transforms difelikefalin from a full agonist into a G protein–biased agonist. In vivo mouse behavioral studies demonstrate that, compared with difelikefalin, beta01 more effectively induces antinociceptive and antipruritic effects with greater attenuation of adverse side effects, including sedation and anxiety. Combined analyses of the structures of the KOR–Gi complex bound with difelikefalin and beta01, molecular dynamics (MD) simulations and nuclear magnetic resonance (NMR) data reveal that KOR adopts a unique conformation upon binding beta01. In this unique conformation, KOR exhibits an expanded intracellular binding cavity, leading to less favorable for β-arrestin binding, and consequently supporting attenuated β-arrestin signaling upon beta01 binding. Our findings demonstrate a detailed process for successful structure-based development of KOR peptide agonists with attenuated β-arrestin signaling that exhibit therapeutic efficacy with reduced adverse side effects. These results not only advance the framework for KOR agonist development with reduced side effects but also provide a broader understanding of the biased agonism of GPCRs.

## Results

### Structural mechanism of KOR activation upon difelikefalin binding

The structure of the difelikefalin-bound KOR–Gi complex was determined at a resolution of 2.4 Å (Supplementary Fig. 1) using single-particle cryo-EM. The high resolution of this structure, particularly binding with the peptide agonist difelikefalin, allowed visualization of detail interactions between the residues of difelikefalin and KOR (Fig. 1b).

Difelikefalin is observed to adopt an extended, vertical conformation in the orthosteric binding pocket (OBP) of KOR, with its N-terminus oriented toward the bottom of the ligand pocket formed by transmembrane helices TM3 and TM6-7 (Fig. 1c). The N-terminal D-Phe1 residue is deeply anchored at the bottom of the binding pocket, establishing multiple interactions with KOR. Specifically, the backbone amide group of D-Phe1 forms hydrogen bonds with Q115^2.60^, D138^3.32^, I316^7.39^ and Y320^7.43^, while its carbonyl oxygen engages Q115^2.60^ and Y312^7.35^ (the superscripts refer to the Ballesteros–Weinstein nomenclature). Additionally, the benzene ring of D-Phe1 participates in hydrophobic interactions with V108^2.53^, Y320^7.43^, and W287^6.48^, collectively anchoring the peptide agonist in this location (Fig. 1c). Replacement of certain residues, including V108^2.53^, Q115^2.60^, D138^3.32^, W287^6.48^, I316^7.39^ and Y320^7.43,^ with alanine significantly decreased the potency and efficacy of difelikefalin-mediated KOR activation (Fig. 1e). The D-Phe2 residue of difelikefalin forms a π–π interaction with Y139^3.33^ together with its hydrophobic contacts with I294^6.55^ and V230^5.42^ (Fig. 1c). In addition, D-Leu3 of difelikefalin is embedded in a hydrophobic network defined by V118^2.63^, V134^3.28^, I135^3.29^ and W124^ECL1^, which are located in TM2, TM3, and ECL1 (Fig. 1c). D-Lys4 stabilizes ECL2 and the extracellular end of TM5 through polar interactions with Y209^ECL2^, Y211^ECL2^ and Y219^5.31^, whereas the C-terminus of difelikefalin participates in *van der Waals* interactions with V207^ECL2^ and polar interactions with N122^2.67^ in the extracellular segment of TM2 (Fig. 1c). The critical contributions of these interactions were validated through alanine mutation studies (Fig. 1d and Supplementary Table 2).

The cryo-EM structure (Fig. 1b, c) revealed several general key factors mediating the specificity of difelikefalin binding to KOR. First, difelikefalin exhibits a high positive surface charge, complementally matching with the unique negative charge of the KOR orthosteric pocket. Nevertheless, the homologous orthosteric pockets in MOR, DOR and NOPR lack this complementary charge (Supplementary Fig. 2a–c). Secondly, structural superposition revealed steric clashes between difelikefalin and Y^3.33^ or Y^7.43^ in MOR, DOR or NOPR, preventing the peptide agonist binding to these receptors (Supplementary Fig. 2d). Thirdly, the center residues of the extracellular segment of KOR-TM2 are in close proximity to the C-terminus of difelikefalin, e.g., residue KOR-N^2.67^ can form a hydrogen bond with difelikefalin. However, the outwardly rotated TM2 segments of the other opioid subtypes (MOR, DOR or NOPR) generate a spatial mismatch, destabilizing difelikefalin binding (Supplementary Fig. 2e). Fourthly, KOR possesses an elongated ECL2 that folds into a lid-like structure over the orthosteric pocket. Replacing KOR-ECL2 with the shorter ECL2 of MOR, DOR, or NOPR markedly attenuated receptor activation by difelikefalin, confirming that ECL2 is also crucial for maintaining the high affinity of difelikefalin for KOR (Supplementary Fig. 2e, g). Finally, residues Y^7.35^ and V^2.53^ of KOR can interact with difelikefalin through hydrogen bonding and hydrophobic interactions, respectively, while the homologous positions of the MOR, DOR or NOPR only contains residues with shorter side chains, not forming effective interactions with difelikefalin (Supplementary Fig. 2f).

To identify the key residues responsible for the induction of G protein or β-arrestin signaling by KOR upon difelikefalin binding, systematic alanine scanning of the residues that interact with difelikefalin was performed. Ligand potency (EC_50_) and efficacy (E_max_) were analyzed to assess the effects of difelikefalin-induced G protein or β-arrestin signaling upon KOR activation (Fig. 1d and Supplementary Table 2). Mutagenesis analysis revealed the critical role of Y320^7.43^, as its substitution with alanine significantly impaired β-arrestin recruitment triggered by difelikefalin. Specifically, the EC_50_ for β-arrestin recruitment increased markedly from 30.2 nM to 23.4 μM, accompanied by a reduction in maximal efficacy from 100% to 20%. In contrast, G protein signaling showed only a modest decrease in potency, with the EC_50_ shifting from 2.63 nM to 42.7 nM, while its maximal efficacy remained largely unchanged (Fig. 1e). These findings suggest that Y320^7.43^ plays a more pivotal role in KOR-mediated β-arrestin signaling than in G protein activation. Importantly, the residue Y^7.43^ is highly conserved across all opioid receptor subtypes (Supplementary Fig. 3a). To assess whether this role is shared by other opioid receptors, we introduced the equivalent Y^7.43^ mutations in both MOR and DOR. In both cases, mutating Y^7.43^ led to a marked reduction in β-arrestin recruitment, while exerting minimal effects on G protein signaling (Supplementary Fig. 3b–e and Supplementary Table 3). Taken together, these findings establish that Y^7.43^ is the key residue modulating β-arrestin signaling across the opioid receptor family, highlighting its potential as a key target for designing biased ligands.

### Difelikefalin-derived KOR peptide agonist development

**Fig. 2:**
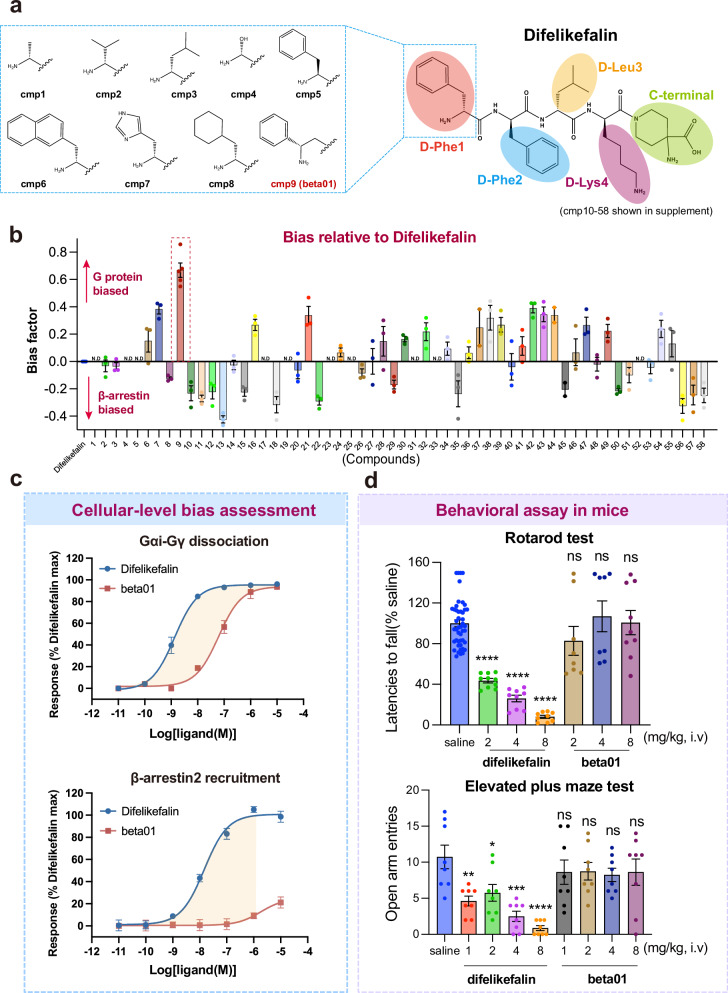
Structure-based development of difelikefalin analogs with reduced β-arrestin2 activities and side effects. **a** Design of difelikefalin derivatives by modifying the phenylalanine at the N-terminus. **b** Bias factors of difelikefalin derivatives relative to difelikefalin. Calculation of the bias factor is detailed in the methods. Bias factor >0 indicates Gi signaling biased while bias <0 implies β-arrestin2 signaling. Data shown are means ± S.E.M. from three independent experiments performed in technical duplicates. N.D means not detectectable. Source data are provided as a Source Data file. **c** Concentration-dependent activation of G-protein and β-arrestin2 signaling in response to difelikefalin and beta01. Data are the mean ± SEM of three independent experiments performed in triplicate. Source data are provided as a Source Data file. **d** Sedation and anxiogenic effect was tested on the rotarod test and the elevated plus maze respectively. Data are presented as the mean ± S.E.M. from at least 6 mice/data point (In rotarod assay, *n* = 44 for saline; 2 mg/kg difelikefalin, *n* = 10, *p* < 0.0001; 4 mg/kg difelikefalin, *n* = 9, *p* < 0.0001; 8 mg/kg difelikefalin, *n* = 9, *p* < 0.0001; 2 mg/kg beta01, *n* = 8, *p* > 0.05; 4 mg/kg beta01, *n* = 8, *p* > 0.05; 8 mg/kg beta01. *n* = 9, *p* > 0.05; In the elevated plus maze test, *n* = 8 for saline; 1 mg/kg difelikefalin, *n* = 8, *p* = 0.0038; 2 mg/kg difelikefalin, *n* = 8, *p* = 0.0257; 4 mg/kg difelikefalin, *n* = 8, *p* = 0.0004; 8 mg/kg difelikefalin, *n* = 8, *p* < 0.0001; 1 mg/kg beta01, *n* = 8, *p* > 0.05; 2 mg/kg beta01, *n* = 8, *p* > 0.05; 4 mg/kg beta01. *n* = 8, *p* > 0.05; 8 mg/kg beta01, *n* = 8, *p* > 0.05. All statistical tests used were two-tailed t-tests). *P < 0.05, **P < 0.01, ***P < 0.001, ****P < 0.0001; ns, not significant *p* > 0.05. Source data and exact *p*-value are provided as a Source Data file.

Because residue Y320^7.43^ is important to dictate the biased signaling profile of KOR upon difelikefalin binding, we focused on the first N-terminal residue D-Phe1, which was observed to directly interact with Y320^7.43^ through a combination of π–π stacking and hydrogen bonding interactions (Fig. 1c). We therefore synthesized a series of difelikefalin-derived peptides (cmp1-cmp9) with various D-Phe1 substitutions (Fig. 2a). These peptide derivatives were evaluated in terms of their potency, efficacy and functional selectivity by assessing Gαi1-γ2 dissociation and β-arrestin2 recruitment (Fig. 2b). The peptides with various of D-Phe1 substitution of difelikefalin consistently modulated KOR downstream signaling bias and particularly affected β-arrestin signaling (Supplementary Fig. 4 and Supplementary Table 4). Among them, the most prominent peptide is compound 9 (cmp9), with D-Phe1 of difelikefalin substituted with β-phenylalanine (named as beta01) (Supplementary Fig. 5). Beta01 exhibited pronounced differences in potency and efficacy in inducing G protein and β-arrestin2 signaling, effectively activating the G protein signaling pathway while exhibiting negligible effects on β-arrestin signaling (Fig. 2c). Furthermore, other 49 difelikefalin peptide derivatives were synthesized with changes to residues other than 1 (D-Phe1). Function analysis of KOR (especially the potency and efficacy) upon binding of these peptides revealed either abolished Gi potency or failure to enact signaling bias (Fig. 2b, Supplementary Fig. 6 and Supplementary Table 4). These observations underscore the unique importance of the pair of interacting residues, D-Phe1 of difelikefalin and KOR-Y320^7.43^, which might dictate downstream signaling pathway selectivity. Essentially, beta01 retained the exceptional KOR subtype selectivity of difelikefalin over MOR, DOR or NOPR (Supplementary Fig. 2h), indicating that G protein bias can be modulated with little compromise in terms of target specificity.

### Beta01 elicits neglectable β-arrestin signaling-mediated adverse side effects

To evaluate the therapeutic potential of beta01, comprehensive in vivo evaluations using well-validated murine models were conducted according to previous works^35–38^. In a 4-chloroquine-induced itch model, beta01 suppressed scratching behavior in a dose-dependent manner, achieving nearly complete antipruritic efficacy (≈ 100%) at 0.125 mg/kg (Supplementary Fig. 7a). In the acetic acid writhing assay, beta01 showed antinociceptive effects in a dose-dependent manner, with an estimated EC_50_ of 0.25 mg/kg (Supplementary Fig. 7b). Since beta01 displayed ~2-fold lower potency than difelikefalin at an equivalent dose, doubling the beta01 dose were implemented to restore analgesic magnitude without compromising safety margins.

The main adverse side effects of KOR agonists are sedation, anxiety and depression. To evaluate side effects of sedation, we employed open-field and rotarod assays. Difelikefalin (1 mg/kg) markedly reduced horizontal locomotion (*p* < 0.0001), an effect that intensified with increasing dose. In contrast, the effect of beta01 (up to 8 mg/kg) did not differ from that of the vehicle control (Supplementary Fig. 7c). Similar results were observed in the rotarod test, where mice injected with difelikefalin remained on the rod for a significantly shorter duration than those injected with beta01 did, highlighting the better performance of beta01 (Fig. 2d and Supplementary Fig. 7d). Anxiogenic liability was evaluated in the elevated plus maze test. Difelikefalin reduced the number of open-arm entries and the open-arm distance traveled (*p* < 0.0001), indicating increased anxiety. The open-arm exploration after beta01 administration remained at the level of mice treated with vehicle across the same dose range (Fig. 2d and Supplementary Fig. 7f). Additionally, the tail suspension test revealed that beta01 induced fewer depression-like side effects than difelikefalin did (Supplementary Fig. 7e). These data demonstrated that beta01 elicits effective KOR-mediated analgesia and antipruritic effects, while greatly reduces sedative, anxiogenic and depressive side effects compared with difelikefalin.

### Structural mechanism of attenuated β-arrestin signaling by KOR bound to beta01

**Fig. 3:**
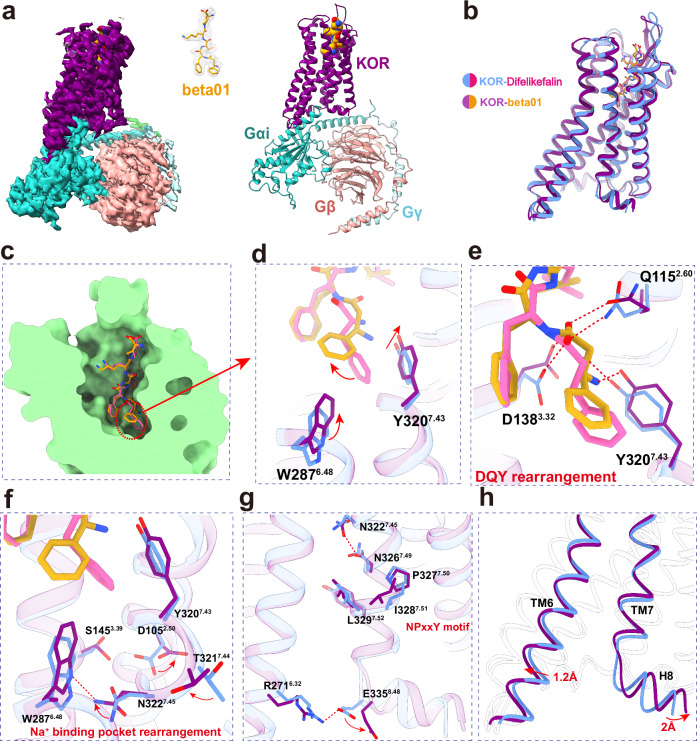
Structure of beta01-bound KOR and the molecular mechanism of reduced β-arrestin effect of beta01. **a** The cryo-EM density maps and molecular model of beta01-KOR-Gi complex. Colored by subunit (beta01 in orangered, KOR in hot purple, Gαi in light sea green, Gβ in light coral, Gγ in powder blue, scFv16 in lime). The density map of beta01 is also shown in the figure. **b** Structure alignment of KOR bound to difelikefalin and beta01. **c**, Superimposition of difelikefalin and beta01 binding pockets in KOR. **d** Comparisons of binding modes of difelikefalin and beta01 in KOR. The phenyl ring of the beta01’s N-terminal phenylalanine has been rotated upwards relative to difelikefalin, leading to the absence of π-π stacking with Y320^7.43^. The side chains of W287^6.48^ and Y320^7.43^ undergo distinct rotameric transitions. **e** The rearrangement of the DQY motif in KOR upon binding to difelikefalin or beta01. **f** The rearrangement of the sodium pocket in KOR upon binding to difelikefalin or beta01. **g** N322^7.45^ induces a conformational change in the NPxxY motif, leading to disruption of the E335^8.48^-R271^6.32^ salt bridge and an upward displacement of helix 8 in the beta01-bound KOR. **h** Conformational changes intracellular ends of TM6 and H8 upon KOR binding with Difelikefalin or beta01. Color usage: difelikefalin and beta01 are colored in hot pink and orange, respectively; difelikefalin bound KOR, cornflower blue; beta01 bound KOR, purple.

To understand how beta01 binding to KOR preserves robust Gi protein signaling while markedly reducing the potency and efficacy of β-arrestin recruitment, we determined the cryo-EM structure of the beta01-bound KOR–Gi complex at a nominal resolution of 2.9 Å (Fig. 3a and Supplementary Fig. 8). Comparing the structures of the difelikefalin- and beta01-bound KOR–Gi complexes, both structures exhibited overall conserved active state architectures (Fig. 3b), characterized by the inward movement of TM5 and TM7 and the outward movement of TM6 relative to their positions in the inactive state of KOR (PDB: 4DJH) (Supplementary Fig. 9).

In the cryo-EM structure of the beta01-bound KOR–Gi complex, the β-Phe1 side chain of beta01 is rotated ~120° upward relative to the position of D-Phe1 in the structure of difelikefalin-bound KOR–Gi complex. This reorientation disrupted the π–π interactions between D-Phe1 and residues Y320^7.43^ and W287^6.48^, which in turn caused W287^6.48^ to tilt upward, displacing Y320^7.43^, which moves outward (Fig. 3c, d). The backbone carbonyl of β-Phe1 is repositioned to form reinforced hydrogen bonds with Q115^2.60^ and D138^3.32^, leading to rearrangement of the DQY motif (Fig. 3e). The upward-shifted W287^6.48^ residue engages in hydrogen bonding interactions that alter the conformation of N322^7.45^, a key residue involved in the formation of the sodium ion pocket. This results in the sodium ion pocket adopting a distinct conformation, with a more pronounced disruption occurring upon beta01 binding, possibly attributing to the increased opening of TM6 (Fig. 3f). In addition, the hydrogen bonding interactions with N322^7.45^ also cause the differential deflection of N326^7.49^, triggering a rearrangement of the NPxxY motif, which is closely associated with the movement of TM7-Helix8 (Fig. 3g). Upon beta01 binding, residue E335^8.48^ at the TM7-Helix8 junction was observed to undergo significant deflection, losing its salt bridge interaction with R271^6.32^, thereby causing relative displacement of Helix8 (Fig. 3g). Collectively, these structural rearrangements result in beta01-bound KOR having a larger intracellular cavity (Fig. 3h and Supplementary Fig. 10).

We hypothesized that this enlarged intracellular cavity may be less favorable for β-arrestin binding, thereby contributing to the attenuated β-arrestin recruitment. This hypothesis aligns with prior observations of angiotensin II type 1 receptor (AT_1_R)^39^, β1 adrenergic receptor (β_1_AR)^40^, and MOR^41^, in which narrower clefts favor β-arrestin binding and larger intracellular pockets are more suitable for G protein binding.

Although the visual conformational differences in the intracellular region are relatively subtle, subsequent molecular dynamics simulations provided quantitative evidence that beta01 binding leads to a significant increase in both the TM6-Helix8 distance and the volume of the intracellular cavity.

To investigate the dynamic responses of KOR upon ligand binding, three independent 500 ns MD simulations were conducted for KOR bound to either difelikefalin or beta01. To minimize the influence of initial structure, we put in the difelikefalin-bound state, TM6-TM7 and Helix8 of KOR move toward the receptor core, characterized by the reduced distance between TM6 and Helix8, which is consistent with the measured distances between Cα of residue I272^6.33^ and Cα of E335^8.48^: 9.35 ± 1.74 Å in the difelikefalin-bound KOR–Gi complex versus 12.22 ± 2.30 Å in the beta01-bound KOR–Gi complex (Supplementary Fig. 11a, b). At the same time, the solvent-accessible surface area (SASA) was measured significantly lower in the difelikefalin-bound structure (1739 ± 123 Å^2^) than those in the beta01-bound structure (1801 ± 117 Å^2^) (Supplementary Fig. 11c, d). The measured larger distance between Cα of I272^6.33^ and Cα of E335^8.48^ and enlarged SASA of KOR upon binding of beta01 versus difelikefalin in MD simulation are consistent with the observed expansion of the intracellular cavity in cryo-EM structure of beta01-bound KOR-Gi complex **(**Fig. 3h and Supplementary Fig. 10**)**. Based on the MD simulation data of KOR bound with difelikefalin or beta01, we calculated the heavy atom root mean square deviation (RMSD) for residues I316^7.39^ - L333^7.56^. These results indicate that difelikefalin, owing to its π-π interaction with Y320^7.43^, stabilizes the conformation of TM7 with a lower RMSD (1.24 ± 0.14 Å), while beta01 binding enhances the dynamic fluctuations of TM7, resulting in a higher RMSD (1.53 ± 0.27 Å) (Supplementary Fig. 11e, f). Such movements were visualized in Supplementary Video. 1, in which cyan KOR-difelikefalin shows TM7 inward movement while magenta KOR-beta01 shows outward TM7 movement.

To further validate the hypothesis of biased signaling, we conducted simulations for KOR^WT^-cmp7 and KOR^WT^-cmp13 since they are G protein and β-arrestin biased ligands, respectively. As shown in Supplementary Fig. 12, in the field of TM6-TM7 distance and SASA, cmp13 takes similar mode with difelikefalin to narrowed the intracellular pocket of KOR (distance: 8.83 ± 1.57 Å, SASA: 1656 ± 116 Å^2^). In contrast, cmp7 acts in the same way as beta01 to enlarge pocket and performs as G protein biased agonist (distance: 12.23 ± 1.87 Å, SASA: 1781 ± 136 Å^2^). These results demonstrate that cmp7 and cmp13 differentially regulate the intracellular pocket of KOR in a manner consistent with their respective signaling biases.

From the perspective of KOR mutants, we also run MD simulations for KOR^Y7.43A^-difelikefalin and KOR^Y7.43A^-beta01. Besides, for both ligands, the Y^7.43^A mutation leads to an increase in the TM6-TM7 distance and SASA compared with the KOR^WT^-difelikefalin system (KOR^Y7.43A^-difelikefalin: 11.99 ± 2.45 Å and 1767 ± 155 Å^2^, KOR^Y7.43A^-beta01: 11.12 ± 1.56 Å and 1764 ± 145 Å^2^) (Supplementary Fig. 13). Consistent with experiments, these observations indicates that the Y^7.43^A promotes a more open intracellular pocket and shifts the receptor towards G-protein signaling.

### Beta01 induces a unique KOR conformation unfavorable for β-arrestin binding

**Fig. 4:**
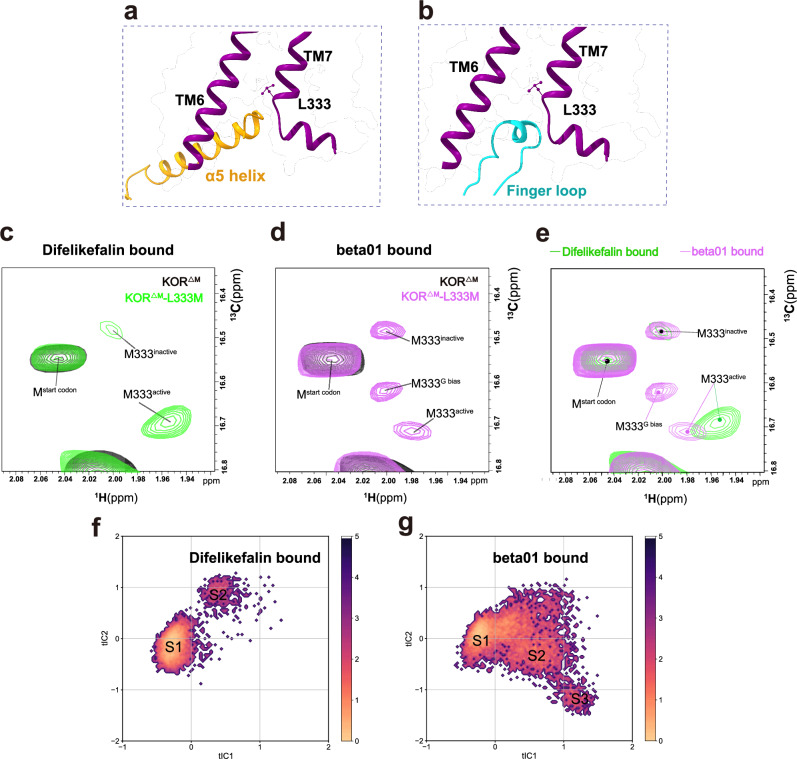
Conformational alterations of the intracellular domain induced by difelikefalin and beta01 in NMR simulations and MD. **a** The binding position of the α5 helix of Gi in KOR. The TM6-Helix8 conformation in cryo-EM structure. The residue L333 is depicted in the figure. **b** The binding site of the β-arrestin finger loop in KOR, as determined by structural alignment of the KOR-Gi complex with the NTSR1-β-arrestin1(PDB 6UP7). The residue L333 is depicted in the figure. **c** ^1^H-^13^C HMQC spectra of the [methyl−^13^C-Met] KOR^△M^ and KOR^△M^-L333M in the difelikefalin bound. **d** ^1^H−^13^C HMQC spectra of the [methyl−^13^C-Met] KOR^△M^ and KOR^△M^-L333M in the beta01 bound. **e** Superposition of HMQC spectra of KOR in complex with difelikefalin and beta01. **f**, **g** Free energy landscapes showing the conformational states of L333^7.56^ in the KOR-difelikefalin (**f**) and KOR-beta01 (**g**) systems in MD. The color bar indicates the free energy values (in kcal/mol). These landscapes were generated from the two-dimensional tICA projections computed using the globally aligned heavy-atom coordinates of L3337.56. S1: -1 < tIC1 < 0, -1 < tIC2 < 1; S2: 0 < tIC1 < 1, -1 < tIC2 < 1; S3: 1 < tIC1 < 2, −2 < tIC2 < −1.

To investigate whether beta01 stabilizes a distinct intracellular conformation of KOR that contribute to attenuated β-arrestin recruitment, we employed NMR spectroscopy to monitor the conformational changes of KOR. From the difelikefalin- (Fig. 1b) and beta01-bound (Fig. 3a) KOR–Gi complex structures, the intracellular residue L333^7.56^ was observed to interact with the α5 helix of the Gi protein (Fig. 4a) and may also contact with the finger loop of β-arrestin (Fig. 4b)^42^. This makes L333^7.56^ an ideal probe to monitor conformational shifts in TM7-Helix8 associated with transducer binding (Fig. 4a, b). To implement the frequently used ^13^CH_3_-Met NMR analysis^43–45^, we engineered a mutant by replacing all Met with Leu and generating a single Met probe at position 333 (named KOR^ΔM^-L333M), retaining the capacity to activate Gi protein and recruit β-arrestin2. Then expressed the mutant KOR in media supplemented with ^13^CH_3_-Met, yielding ^13^CH_3_-Met-labeled KOR protein (Supplementary Fig. 14a, b). Two-dimensional ^1^H−^13^C heteronuclear multiple quantum correlation (HMQC) spectra were acquired for the ^13^CH_3_-Met labeled KOR, bound with different ligands, including various of antagonists or agonists. Upon antagonist (naltrexone) binding, only one signal was observed for L333M^7.56^, which is consistent with a homogeneous inactive state. This signal was designated as L333M^inactive^ (Supplementary Fig. 14c). Upon difelikefalin binding, a well-resolved resonance was observed in the 2D spectrum, indicating a canonical active state. This signal was designated as L333M^active^ (Fig. 4c). Interestingly, upon beta01 binding, three resonances were observed, with two signals exhibiting good overlap with the resonances L333M^inactive^ and L333M^active^, together with another resonance (Fig. 4d). This third resonance might indicate the unique conformation of L333M^7.56^ upon the binding of beta01. Notably, a comparable third signal was also observed in the spectrum of KOR upon binding of nalfurafine, the classical KOR agonist with biased G protein signaling (Supplementary Fig. 14d). These observations suggest the existence of a third conformational state of L333M^7.56^, which may be linked to a receptor conformation associated with G protein biased signaling.

In support of our NMR observations, MD simulations provided further evidence for beta01-induced conformational bias in KOR. Time-lagged Independent Component Analysis (tICA) based on the heavy-atom trajectory of L333^7.56^ revealed that there were two distinct conformational clusters (designated S1 and S2) in the KOR-difelikefalin system (Fig. 4f), whereas three well-defined conformational clusters (designated S1, S2, and S3) were observed in the KOR-beta01 system, highlighting the differences in the states of L333^7.56^ between the two systems (Fig. 4g). Such results provided valuable complementary evidence, confirming that the conformational states of L333^7.56^ differ between the KOR-difelikefalin and KOR-beta01 systems. Together, the NMR and MD data offer a multi-timescale perspective on the conformational landscape of L333^7.56^, demonstrating that L333^7.56^ adopts distinct conformational states in the two ligand-bound systems.

**Fig. 5:**
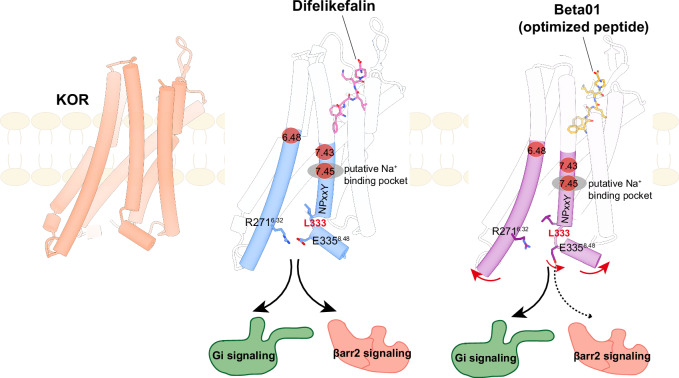
A cartoon model of this study. Beta01 binding induces rearrangements in key KOR motifs, including the putative sodium ion pocket and NPxxY motif. These changes alter the conformations of TM6 and H8, creating an intracellular receptor conformation less conducive to β-arrestin binding. Thus, beta01 exhibited reduced β-arrestin signaling and side effects in mice.

In summary, beta01 was rationally developed by modifying difelikefalin to fine-tune KOR signaling bias. While preserving strong Gi protein activation, beta01 markedly reduces β-arrestin recruitment. Combining structural analysis, MD simulations and NMR studies, we revealed that the binding of beta01 induced distinct rearrangements of key KOR motifs, triggering subtle but distinct rearrangements of the W^6.48^ toggle switch, the sodium ion pocket, and the NPxxY motif. These rearrangements led to conformational changes in TM6 and Helix8, positioning the intracellular region of the receptor in a unique conformation that is less favorable for β-arrestin binding (Fig. 5). These findings establish a structural framework for the further development of KOR agonists, especially G protein-biased therapeutics with improved safety profiles.

## Discussion

In this study, we determined the high-resolution cryo-EM structure of the difelikefalin-bound KOR–Gi complex, providing detailed insights into the structural basis of KOR activation by a clinically approved peptide agonist. Guided by this structure, we performed rational modifications at a key interaction site—specifically, the N-terminal D-Phe1 residue of difelikefalin, which engages in π–π interactions with Y320^7.43^ of KOR. This residue was identified as a crucial determinant in balancing G protein and β-arrestin signaling, as its interaction modulates conformational rearrangements within the receptor’s intracellular cavity.

By substituting D-Phe1 with various unnatural amino acids, we discovered beta01, a β-phenylalanine-modified analog of difelikefalin. Functional assays demonstrated that beta01 retained potent G protein signaling while exhibiting markedly reduced β-arrestin recruitment, representing a Gi protein-biased KOR agonist. Importantly, in murine models, beta01 preserved the strong antinociceptive and antipruritic efficacy of difelikefalin, but significantly reduced adverse side effects such as sedation, anxiogenesis, and depressive-like behaviors—highlighting the therapeutic advantage of biased signaling.

To elucidate the structural mechanism underlying this signaling bias, we solved the cryo-EM structure of the beta01-bound KOR–Gi complex. Compared with the difelikefalin-bound structure, beta01 induced distinct conformational changes in several key microswitches of KOR, including W287^6.48^ (toggle switch), the sodium ion pocket, and the NPxxY motif. These changes led to an outward displacement of TM6 and TM7, as well as a significant deflection of Helix8, collectively resulting in an expanded intracellular cavity that is structurally less favorable for β-arrestin binding.

Supporting these structural findings, NMR spectroscopy using ^13^CH_3_-Met-labeled KOR revealed that beta01 induces a unique conformational ensemble at the intracellular end of TM7, distinguishable from the canonical active and inactive states. Molecular dynamics simulations further confirmed these observations, showing that beta01 stabilizes L333^7.56^ in distinct χ_₂_ rotameric states and induces greater conformational heterogeneity in the intracellular pocket—favoring Gi protein engagement while disfavoring β-arrestin interaction.

Together, these results establish a clear structure-function relationship in which specific ligand–receptor interactions fine-tune the conformational landscape of KOR, directing signaling bias. Our work demonstrates a successful example of structure-based design of a biased GPCR agonist, leveraging structural, functional, and computational analyses to rationally modify a clinically used ligand for improved signaling selectivity and therapeutic safety.

## Methods

### Ethical statement

In this study, all experiments were specifically designed to minimize the number of animals used and were approved by the Animal Ethics Committee of the Hefei Institutes of Physical Science, Chinese Academy of Sciences and were conducted in accordance with the National Institutes of Health Guide for the Care and Use of Laboratory Animals (DWLL (E)-2024-06).

### Constructs

The optimized coding DNA for wild type *Homo sapiens* KOR (UniProt accession: P41145) was synthesized by Genscript. And residues 54–358 of KOR with an N-terminal thermostabilized apocytochrome b562RIL (BRIL)^46^ and a C-terminal LgBiT were cloned into pFastBac vector using homologous recombination (CloneExpress One Step Cloning Kit, Vazyme). The N-terminus was modified with haemagglutinin (HA) signal sequence followed by a Flag tag for expression and purification. In addition, the 8×His tag was fused at the C-terminus of KOR for two-step purification. The Gα_i_ construct was designed with dominant-negative mutations S47N, G203A, A326S, and E245A to decrease the affinity of nucleotide-binding and stable Gαβγ complex^47^. Rat Gβ_1_ with an N-terminal His6 tag was followed by HiBiT at its C terminus. Rat Gβ1 was fused with a HiBiT at C-terminal for structural complementation of LgBiT to form a NanoBiT^48^. The single-chain variable fragment scFv16 was applied to bind the Gα_i_βγ protein for stabilization^49^. Gα_i_, Gβ_1_-HiBiT, Gγ, and scFv16 were cloned into the pFastBac vector separately (Invitrogen). The wild-type and mutants of KOR were constructed into the pcDNA3.1 vector for functional assays.

### Expression and purification of agonists-KOR–G_i_ complex

*Spodoptera frugiperda* (Sf9) insect cells were grown in ESF 921 medium to a density of 2.0 × 10^6^ cell per ml and were then co-infected with five separate baculoviruses at a ratio of 1:1:1:1:1 for KOR-LgBit, DN-Gα_i1_, Gβ_1_-HiBiT, Gγ_2_ and scFv16, while for difelikefalin-KOR-Gi complex, we used KOR, DN-Gα_i1_, Gβ_1_, Gγ_2_ instead of these five baculoviruses above-mentioned to form the stable signaling complex. After culture for 48 h at 27 °C, the cells were collected by centrifugation and cell pellets were then stored at −80 °C for future use.

The cell pellets were lysed by dounce homogenization in 20 mM HEPES pH7.4, 100 mM NaCl, 10 mM MgCl_2_, 5 mM CaCl_2_, 10% glycerol, 100 μM TCEP (Sigma-Aldrich), 25 mUml^−1^ Apyrase (Sigma-Aldrich), EDTA-free protease inhibitor cocktail (TargetMol, USA), 20 μM peptide (difelikefalin, MedChemExpress；beta01, Hefei KS-V Peptide Biological Technology Co. Ltd) and 20 μg mL^−1^ scFv16 to stimulate the formation of signaling complex. The suspension was continually incubated for one hour at room temperature before being extracted from the membrane using 1% (w/v) lauryl maltose neopentylglycol (LMNG, Anatrace) and 0.1% (w/v) cholesteryl hemisuccinate TRIS salt (CHS, Anatrace). After membrane solubilization for 3 hours at 4 °C, the solubilized fractions were isolated by centrifugation at 100,000 *× g*. for 45 min. For difelikefalin-KOR-Gi complex, the solubilized fractions were incubated overnight at 4 °C with pre-equilibrated Nickel-NTA resin using distilled deionized water. After batch binding, the nickel resin with immobilized protein complex was manually loaded onto a gravity flow column. The nickel resin was firstly washed with 20 column volumes of 20 mM HEPES, pH 7.4, 100 mM NaCl, 25 mM imidazole, 0.3 mM TCEP, 0.05% LMNG (w/v), 0.01% CHS (w/v) and 10 mM difelikefalin, and then eluted with the same buffer containing 300 mM imidazole. The Ni-NTA eluates were further incubated with pre-equilibrated anti-Flag G1 Affinity resin (GenScript) for 1 h at 4 °C. For beta01-KOR-G_i_ complex using one-step purification, the solubilized fractions were incubated with pre-equilibrated anti-Flag G1 Affinity resin for 1 h at 4˚C. The resin was collected by centrifugation at 500 *×g* for 10 min, and then loaded onto a gravity flow column and washed with 10 column volumes of buffer containing 20 mM HEPES pH 7.4, 100 mM NaCl, 10 mM MgCl_2_, 5 mM CaCl_2_, 10% glycerol, 100 μM TCEP, 10 μM peptide, 0.1% (w/v) LMNG and 0.01% (w/v) CHS. And then protein bound resin was washed with 10 column volumes of wash buffer containing 20 mM HEPES pH7.4, 100 mM NaCl, 10 mM MgCl_2_, 5 mM CaCl_2_, 10% glycerol, 100 μM TCEP, 20 μM peptide, 0.03% (w/v) LMHG, 0.01% (w/v) glyco-diosgenin (GDN, Anatrace) and 0.003% (w/v) CHS. The complex was eluted by 5 column volumes of elution buffer containing 20 mM HEPES (pH 7.5), 100 mM NaCl, 5 mM MgCl_2_, 0.03% (w/v) LMNG, 0.01% (w/v) GDN and 0.003% (w/v) CHS and 200 μg/mL Flag peptide. The collected protein was concentrated and further purified by size-exclusion chromatography on Superdex 200 10/300 GL column (GE Healthcare) with running buffer containing 20 mM HEPES (pH 7.5), 100 mM NaCl, 5 mM MgCl_2_, 0.00075% LMNG, 0.00025% GDN, 0.000075% CHS, 20 μM peptide. The peak of complex was collected and concentrated with an Amicon Ultra Centrifugal Filter (MWCO 100 kDa) to 1.2 mg/mL for cryo-EM sample preparation.

### Expression and purification of scFv16

ScFv16^49^ with a His8 tag at C-terminus was expressed in *Sf9* insect cells and subsequently purified as follows. In detail, the cells infected with scFv16 virus for 48 h were collected by centrifugation (4000 *× g*, 10 min). The supernatant was purified with nickel affinity chromatography. The precipitates were resuspended in HEPES buffer (20 mM HEPES, pH 7.5, 100 mM NaCl), and then the cells were disrupted. And the supernatant obtained after high-speed centrifugation (20,000 *× g*, 30 min, 4 °C) was also purified with nickel affinity chromatography. The Superdex 200 Increase 10/ 300 GL column (GE Healthcare) was used to separate the monomeric fractions of scFv16 with running buffer containing 20 mM HEPES, pH 7.5, 100 mM NaCl, 2 mM MgCl_2_. The purified scFv16 was flash-frozen by liquid nitrogen and stored at −80 °C until use.

### Cryo-EM grid preparation and data collection

Cryo-EM grids were prepared using a Vitrobot Mark IV plunger (FEI) set at 4 °C and 100% humidity. A three-microliter drop of the difelikefalin-bound KOR-Gi complex was applied to the glow-discharged EM grids (Quantifoil R1.2/1.3 holey carbon films, 300 mesh Au). The sample was incubated on the grids for 5 s before being blotted for 3 s (double-sided, blot force 3) and then immediately flash-frozen in liquid ethane. The same preparation conditions were applied to the beta01-bound KOR-Gi complex samples.

For the difelikefalin-bound KOR-Gi complex, a dataset consisting of 6,015 movies was collected on a Titan Krios equipped with a Gatan K3 direct electron detection device at 300 kV, with a magnification of 105,000, resulting in a pixel size of 0.824 Å. Image acquisition was performed using EPU Software (FEI Eindhoven, Netherlands), capturing a total of 36 frames with an accumulated dose of 50 e⁻ Å⁻² over 2.5 s of exposure. Each movie was divided into 36 frames during motion correction.

For the beta01-bound KOR-Gi complex, a dataset of 8,429 movies was collected on a Titan Krios equipped with a Falcon4 direct electron detection device at 300 kV, with a magnification of 165,000, corresponding to a pixel size of 0.73 Å. Each EER format movie was collected with a total dose of 50 e⁻ Å⁻² over 2.5 s of exposure.

### Cryo-EM data processing

All dose-fractionated images were motion-corrected and dose-weighted using MotionCorr2 software^50^, and their contrast transfer functions were estimated using patch CTF estimation^51^ in cryoSPARC v4.5.3. Subsequent steps were carried out within cryoSPARC.

For the difelikefalin-bound KOR-G_i_ dataset, 1,079,900 particles were automatically picked and extracted from 3000 cryo-EM micrographs. Following two rounds of reference-free 2D classification, 299,597 particles were selected as references for template picking from the total of 6015 cryo-EM micrographs. This was followed by two additional rounds of 2D classification and four rounds of heterogeneous refinement, resulting in 623,181 particles. After homogeneous and non-uniform refinement, these particles were reconstructed to a resolution of 2.43 Å.

For the beta01-bound KOR-Gi dataset, particles generated by Blob picking underwent 2D classification to create input particles for both template picking and Topaz picking. After two rounds of 2D classification, 862,355 particles were selected for heterogeneous refinement, which ultimately yielded 431,242 particles for final non-uniform refinement. These particles were reconstructed to a resolution of 2.86 Å.

### Model building and refinement

The cryo-EM structure of the dynorphinA-KOR-Gi-scFv16 (PDB: 8F7W) was utilized to construct models for both the difelikefalin-bound and beta01-bound KOR-Gi complexes. All models were fitted into the EM density map using UCSF Chimera^52^, followed by iterative rounds of manual adjustment and automated rebuilding in COOT^53^ and PHENIX^54^, respectively. Final model statistics were validated using the Comprehensive Validation (cryo-EM) feature in PHENIX and are detailed in the extended table. All structural figures were generated using Chimera^52^, Chimera X^55^, and PyMOL (https://pymol.org/2/).

### Experimental Procedure for Synthesis of Difelikefalin Derivatives

All chemicals and solvents were commercially available and used as received without further purification. All difelikefalin derivatives were synthesized primarily via 9-fluorenylmethyloxycarbonyl-based solid-phase peptide synthesis (Fmoc-SPPS), starting from 0.4 mmol 2-Cl Trityl resin (0.4 mmol/g). Building blocks were used at 4 equiv and coupled sequentially from the C-terminus to the N-terminus. After chain elongation, a trifluoroacetic acid (TFA) cleavage cocktail (TFA:H₂O:TIPS:phenol = 85:5:5:5) was employed to cleave the peptide from the resin and remove side-chain protecting groups. All crude peptides were further purified by semi-preparative HPLC (SHIMADZU). The purities of all final products were determined to be above 95% by reversed-phase high-performance liquid chromatography (HPLC, SHIMADZU), and their molecular weights were verified by ESI-MS (SHIMADZU LCMS-2020).

### NanoBiT based G-protein dissociation assay

HEK 293 T cells were cultured in DMEM (Gibco, Thermo Fisher Scientific) supplemented with 10% FBS and 1% penicillin/streptomycin at 37 °C, and 5% CO_2_. The cells were transiently transfected with wild type or mutant KOR, Gα_i_-LgBiT, Gβ and Gγ-SmBiT using Lipofectamine 3000 (Thermo Fisher Scientific). Cells were distributed into 96-well microplates with a density of 5 × 10^4^ cells per well after 24 hours transfection and incubated with 50 μM furimazine (TargetMol, USA) at room temperature in the dark for 50 min. Luminescence signals were measured for 10 min as baseline using Multifunctional Microplate Reader (FlexStation3, Molecular Devices), and then read for 20 min after addition of test agonists (6× diluted in OMEM, 20 μL per well). Data analysis was performed using the non-linear regression algorithms (three-parameter logistic curve) in Prism (GraphPad Software, USA) to calculate the values of E_max_ and EC_50_. Data points represent three independent experiments performed in triplicate. Statistics for mutational studies were performed using one-way analysis of variance followed by Dunnett’s test with wild-type receptors as the control. The primer sequences used for site-directed mutagenesis are listed in Supplementary Table 5.

### NanoBiT based β-arrestin2 recruitment assay

The recruitment of β-arrestin2 to KOR was detected in HEK293T cells using the NanoLuc Binary System. The full-length KOR (1–380) was cloned into pcDNA3.1 vector with a Flag tag at its N terminus and LgBiT at its C terminus. Human β-arrestin2 was cloned into pcDNA3.1 vector with a SmBiT at its N terminus. HEK293T cells were transfected with KOR-LgBiT (WT or mutants) and SmBiT-β-arrestin2 plasmids in equal proportions with Lipofectamin3000 (Invitrogen). After 24 h, the transfected cells were plated into 96-well microplates in OMEM (Gibco) at 50000 cells/80 μL/well. Next, the cells were added into 50 μM furimazine (TargetMol, USA) and incubated for 50 min at room temperature. Luminescence signals were measured for 10 min as baseline using Multifunctional Microplate Reader (FlexStation3, Molecular Devices), and then read for 20 min after addition of test agonists (6× diluted in OMEM, 20 μL per well). Each mutant was performed in three independent experiments. The data were analyzed using Graphpad Prism software 9.0. The primer sequences used for site-directed mutagenesis are listed in Supplementary Table 5.

### Cell surface expression level analysis

Cell surface expression level of KOR and mutants were determined by flow cytometry. HEK293T cells were cultured in DMEM (Gibco, Thermo Fisher Scientific) supplemented with 10% v/v FBS at 37 °C and 5% CO_2_. The cells were seeded in six-well plates 24 h before transfection. And then the cells were transiently transfected with KOR wild-type or mutants using Lipofectamine 3000 (Invitrogen) for 24 h. After transfection, cells were washed twice with 1 mL PBS (Gibco,10010023) containing 3% BSA. The cells were then pelleted and resuspended in 200 μL PBS containing 3% BSA and 1 μL PE-anti-DYKDDDDK tag (BioLegend, Cat: 637310). After incubation in a 4 °C and dark environment for 30 minutes, the cells were washed twice with PBS and the expression level were detected by flow cytometry (BD Biosciences). The FACS data were analyzed by BD Accuri C6 software 1.0.264.21. All mutants’ expression level were normalized to the expression of wild-type opioid receptors. Each mutant was performed in three independent experiments. The gating strategy and calculation method of expression are shown in Supplementary Fig. 15.

### Animal administration

Male KM mice (6–8 weeks old) were obtained from Jiangsu Huachuang Xinnuo Pharmaceutical Technology Co., Ltd. Male C57BL/6 J mice (6–8 weeks old) were obtained from Hangzhou Ziyuan Experimental Animal Technology Co., Ltd. Male ICR mice (6–8 weeks old) were obtained from Hangzhou Ziyuan Experimental Animal Technology Co., Ltd. The mice were housed at the Hefei Institutes of Physical Science, Chinese Academy of Sciences facility under standard laboratory conditions. These conditions included a 12 h light/dark cycle, a temperature of 20–22 °C, and humidity ranging from 40% to 70%. The mice had ad libitum access to water and food throughout the study.

### Abdominal constriction test

Abdominal constriction tests were performed in Kunming mice (male, 18-22 g) according to the method previously described^34,35^. In datail, an abdominal constriction was defined as a wave of contraction of the abdominal musculature followed by extension of the hind limbs. After receiving graded doses of drugs 20 min before the test (i.v., 10 mL/kg body weight), an intraperitoneal injection of 0.8% acetic acid (10 mL/ kg body weight) was administered to each mouse, and then the number of writhing signs displayed by each mouse was counted for 20 min. Antinociception was evaluated using percentage analgesia expressed as 100 × (no. of mean control abdominal constriction - no. of test abdominal constriction)/no. of mean control abdominal constriction. The antinociceptive ED50 value of each compound was obtained as the dose that produced 50% analgesia. Each experiment group has no less than six mice, and data points were excluded from the analysis only if they were identified as statistical outliers by Grubbs’ test (*α* = 0.05).

### Scratching test

The experiment was carried out according to the previous study^17,35^ with C57BL/6 J mice (male, 18–22 g). In detail, mice were habituated to clear acrylic testing boxes for 1 h prior to the start of the experiment. Mice were injected with test compounds (0.5–3.0 mg/kg, i.v., −20 min), then received chloroquine diphosphate (3.4 mg/mL, 300 μL, s.c._neck_) injection to induce acute pruritus. Immediately after that, the number of scratching bouts was recorded for 20 min. One bout of scratching was defined as a lifting of either hind paw to scratch the injection region of body and then replacing it back to the floor. Anti-scratching was evaluated using percentage pruritus expressed as 100 × (basal scratches number - scratches number after drug treatment) / basal scratches number. Each experiment group has no less than six mice, and data points were excluded from the analysis only if they were identified as statistical outliers by Grubbs’ test (*α* = 0.05).

### Spontaneous locomotor activity

The experiment was carried out according to the previous study^22,36^. In detail, Kunming mice (male, 18-22 g) were monitored in an open field activity monitor (Versamax [20 × 20 cm^2^] by Accuscan Instruments) immediately following injection (10 mL/kg weight, i.v.) with vehicle or test compounds. Spontaneous activity was measured over 15 min, before each experiment, the test equipment should be wiped clean with 75% alcohol to prevent the influence of odor on the test results. Each experiment group has no less than six mice, and data points were excluded from the analysis only if they were identified as statistical outliers by Grubbs’ test (*α* = 0.05).

### Rotarod test

The experiment was carried out according to the previous study^35,36,56^ with ICR mice (male, 18–22 g). Before the formal experiment begins, it is necessary to train the mice first. The mice were trained three times per day for three consecutive days, and mice with baseline >160 s were screened for the following test. On the day of testing, following baseline determination from three trials, mice were tail vein injected with either saline, difelikefalin (2, 4 and 8.0 mg/kg, −20 min) or different doses of beta01 (2, 4 and 8.0 mg/kg, −20 min, −1, −2, −3, −4, −5, −6, −7, or −8 h). Time duration for each mouse to fall was recorded. Each experiment group has no less than six mice, and data points were excluded from the analysis only if they were identified as statistical outliers by Grubbs’ test (*α* = 0.05).

### Elevated plus maze test

The experiment was carried out according to the previous study^57,58^ with KM mice (male, 18–22 g). Mice were tail vein injected with either saline, difelikefalin (1, 2, 4 and 8.0 mg/kg, −20 min) or different doses of beta01 (1, 2, 4, and 8.0 mg/kg, −20 min). Mice were individually placed in the center, facing an open arm, and allowed to explore for 5 min. Based on the intrinsic characteristics27, animals tend to avoid open arms because of higher levels of fear and anxiety, so, anxiety-related behavior is quantified for 5 min by the total number of entries into the open and closed arms. The apparatus was cleaned using 75% ethanol after each animal. Each experiment group has no less than six mice, and data points were excluded from the analysis only if they were identified as statistical outliers by Grubbs’ test (α = 0.05).

### Tail suspension test

The experiment was carried out according to the previous study^59^ with a minor modification. KM mice (male, 18-22 g) were tail vein injected with either saline, difelikefalin (2, 4 and 8.0 mg/kg, −20 min) or different doses of beta01 (2, 4 and 8.0 mg/kg, −20 min). Acoustically and visually isolated mice were suspended by their tail from a ledge with adhesive tape, 10 cm above the tabletop, for 6 min. The tape was placed approximately 1 cm from the tip of the tail. Immobility time, defined as the period during which each mouse was not struggling, with the time of immobility recorded during the last 5 min of the total suspended time by using a timer. Each experiment group has no less than six mice, and data points were excluded from the analysis only if they were identified as statistical outliers by Grubbs’ test (*α* = 0.05).

### Molecular dynamics simulations

A total of 6 simulation systems including KOR^WT^-difelikefalin, KOR^WT^-beta01, KOR^WT^-cmp7, KOR^WT^-cmp13, KOR^Y7.43A^-difelikefalin and KORY^7.43A^-beta01 were constructed. To address the concern regarding potential bias from different starting models, we developed the systems from the KOR-difelikefalin complex. The small molecule beta01 was aligned to the binding pocket of difelikefalin. Other ligands, including cmp7 and cmp13, were constructed on the difelikefalin-based structure to ensure consistent binding poses. The KOR Y^7.43^A mutant systems were generated by introducing the Y^7.43^A mutation into both the KOR-difelikefalin and KOR-beta01 complexes. The complexes were embedded into a 75 × 75 Å POPC lipid bilayer using the packmol-memgen software suite^60^, and the systems were subsequently surrounded by a 12 Å thick TIP3P water layer. Ionic strength was maintained at 0.15 mol/L NaCl, with additional counterions introduced to neutralize the system. The protonation assignments were determined using PROPKA 3.1^61^ at physiological pH (pH 7.0). All histidine residues were visually inspected, and their protonation states (HIS/HIE/HID/HIP) were assigned based on local hydrogen-bonding environments and known structural context within KOR. The final protonation states were validated before system minimization and equilibration. The FF19SB, Lipid21, and GAFF2 force fields were utilized for amino acids, lipids, and ligands, respectively^62–64^.

Each system underwent energy minimization followed by a heating and equilibration process, adhering to established protocols^65,66^. Three independent production runs, each 500 ns in duration, were conducted using pmemd.cuda in Amber24^67^. NVT ensemble at 300 K and 1 atm were applied. Long-range electrostatic interactions were treated with the Particle Mesh Ewald method and grid size was 1 Å, while a 10 Å cutoff was applied for short-range electrostatic and van der Waals interactions. The SHAKE algorithm and hydrogen mass repartitioning were utilized to constrain hydrogen-containing bonds, allowing a timestep of 4 fs^68^.

Analyses of the simulation trajectories were performed using CPPTRAJ, including calculations of root-mean-square deviation (RMSD), solvent-accessible surface area (SASA), the cluster of representative structure, and interatomic distances. Additionally, the conformational clusters of L333^7.56^ were characteriezd using time-lagged Independent Component Analysis (tICA)^69^.

### Expression and purification of KOR for NMR studies

The two constructs utilized in the NMR experiments were derived from the constructs previously employed for structural determination. Specifically, KOR^∆M^ was generated by mutating all methionine residues, with the exception of the initial methionine, to leucine in the original construct. KOR^∆M^-L333M was subsequently obtained by introducing a point mutation at L333 to methionine in the KOR^∆M^ construct via site-directed mutagenesis PCR. High titer baculoviruses encoding KOR^∆M^ or KOR^∆M^-L333M genes were used to infect Sf9 cells at a cell density of 2.0 × 10^6^ cells per mL in suspension in methionine deficient media (Expression System). After 12 h, ^13^C methyl labeled methionine (Cambridge Isotope) was added to the culture medium at a final concentration of 50 mg/L. The cells were then cultured for an additional 24 hours. Subsequently, the cells were harvested and stored at 80 °C for further use. The purification procedure was identical to that previously described. Throughout the purification process, the ligand was consistently added at a concentration of 10 μM.

### NMR experiments

NMR samples were prepared in > 99% D₂O containing 20 mM HEPES, 100 mM NaCl, 5 mM MgCl₂, 0.00075% LMNG, 0.00025% GDN, and 0.000075% CHS, with the pH adjusted to 7.5. The NMR experiments were conducted using an Avance III 600 MHz Bruker spectrometer equipped with a TX1 cryogenic probe. A volume of 500 μL of each sample was transferred into a 5 mm NMR tube. The protein concentration in the samples was approximately 70 μM. Two-dimensional ¹H-¹³C heteronuclear multiple quantum coherence (HMQC) spectra were acquired at a temperature of 298 K with a ^1^H spectral width of 5.5 ppm and a ^13^C spectral width of 14.0 ppm, and 1760 scans per spectrum. The 2D NMR spectra were subsequently processed utilizing the Topspin 4.4.0 software (Bruker).

### Statistical analysis

Statistical analyses were performed on at least three individual data sets analyzed by GraphPad prism 9.0. Data are means ± SEM from at least three independent experiments performed in technical triplicate. For NanoBiT G-protein dissociation assay, data was normalized to 100% of lowest concentration, and for NanoBiT β-arrestin2 recruitment assay, data was normalized to 100% of maximal concentration of WT stimulation. And the data was analyzed using nonlinear curve fitting for the log (agonist) versus response (three parameters) curves.

The signaling bias factor^13^ was analyzed through the $\triangle\triangle$logRAi parameter, which reflects the differences in both the efficacy and the potency of two different pathways, as show in the following equation.

1 $$\triangle \triangle \log {RAi}=\log \left(\left[\frac{E\max,P1\times {EC}50,P2}{{EC}50,P1\times E\max,P2}\right]{ligand} \right. \\ \left. \times \left[\frac{E\max,P2\times {EC}50,P1}{{EC}50,P2\times E\max,P1}\right]{reference}\right)$$

Where P1 is Gα_i_ protein signaling data; P2 is β-arrestin2 recruitment assay data; $\triangle\triangle$logRAi >0 is Gα_i_ signaling biased, and $\triangle\triangle$logRAi <0 is β-arrestin2 signaling biased.

### Reporting summary

Further information on research design is available in the Nature Portfolio Reporting Summary linked to this article.

## Supplementary information

Supplementary Information

Description of Additional Supplementary Files

Supplementary Video 1

Reporting Summary

Transparent Peer Review File

## Source data

Source Data

## Data Availability

The cryo-EM density maps and atomic coordinates have been deposited in the Electron Microscopy Data Bank (EMDB) and Protein Data Bank (PDB) under accession numbers EMD-68217 and 22ES for the difelikefalin-KOR-Gi complex, and EMD-68208 and 22EM for the beta01-KOR-Gi complex. All the raw data from our molecular dynamic simulations have been uploaded to the public repository Zenodo (https://zenodo.org/records/17984759). The relevant source data from each figure or table in the main manuscript and in the Supplementary Information are provided as Source Data file. Source data are provided with this paper.
