# Supplementary material for: Rational design of a Kappa opioid receptor peptide agonist with attenuated β-arrestin signaling
Source: Nat Commun. 2026 Apr 14;17:5439. doi: 10.1038/s41467-026-71455-3 (PMC13279772; doi:10.1038/s41467-026-71455-3)
Supplement: Supplementary file 1 — Supplementary Information [file 41467_2026_71455_MOESM1_ESM.pdf]

Supplementary Information for

Rational design of a kappa opioid receptor peptide agonist with attenuated β-arrestin signaling

Huanhuan Zhang[1,2,#], Ruolan Wang[3,4,#], Pan Shi[1,2,#,*], Gaoming Wang[12#] , Qingjun Zhu[5,#], Xinheng He[3,4,],Youwei Xu[3], Qingning Yuan[3,6,7], Wen Hu[3,6], Kai Wu[3,6], Yong Zheng[2], Li Zhou[2], Jun Liang[1,2], Pei Lv[8], Ziyan Xu[1,2], Fan Yang[1], Yingbin Liu[12], Youwen Zhuang[9], H. Eric Xu[3,4,7,*], Yue Wang[3,*], Changlin Tian[1,2,10,11*]

This PDF file includes:

Supplementary Figures 1 to 15

Supplementary Tables 1 to 5

Uncropped gels

Supplementary Notes (certificates of compound analysis)

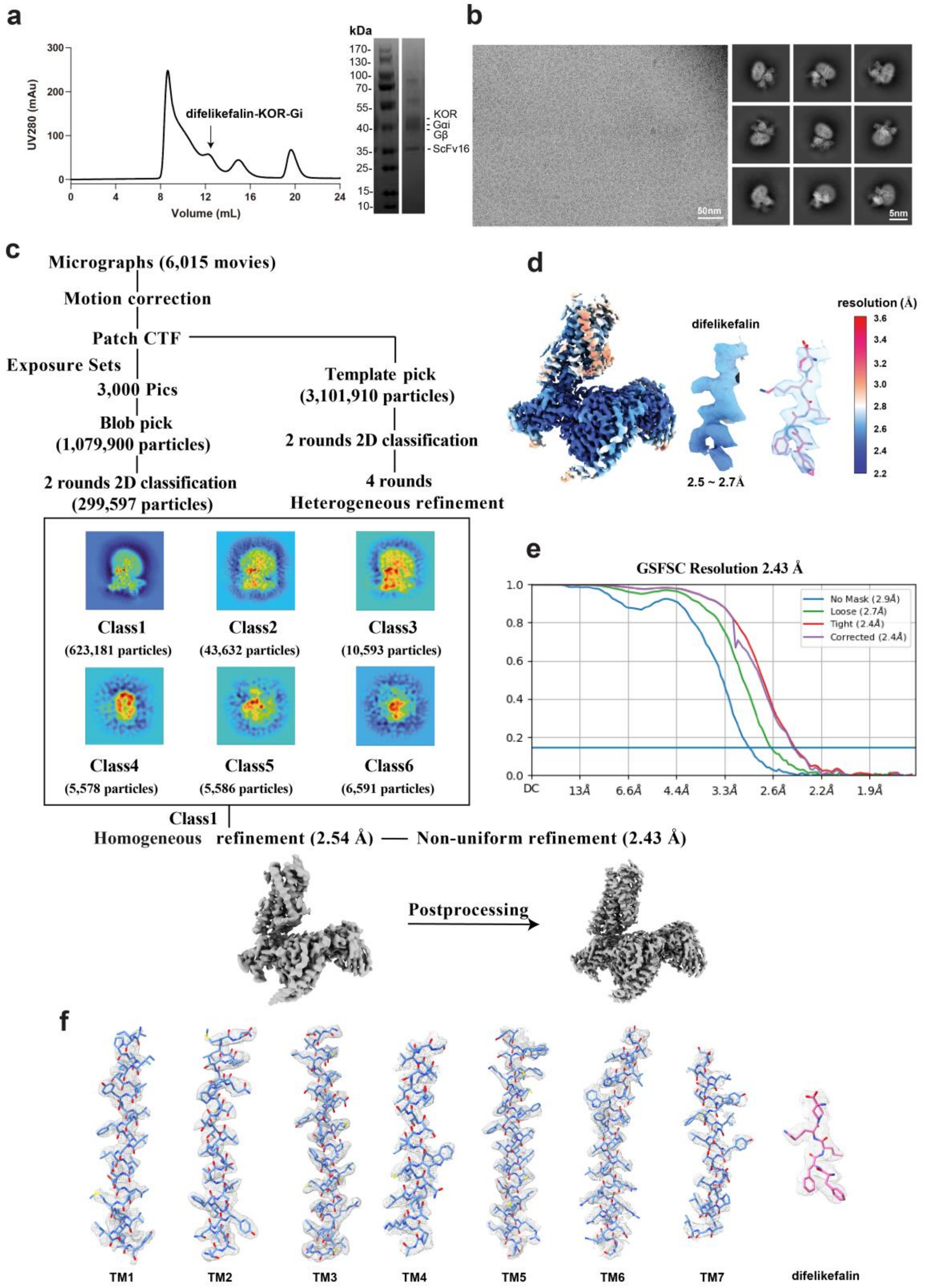

Supplementary Fig. 1 | Purification and cryo-EM data processing of difelikefalin bound KOR-Gi complex. a, Size-exclusion chromatography profile and SDS-PAGE analysis of difelikefalin-KOR-Gi complex. **b,** Representative micrograph after motion correction and dose weighting and 2D class averages of difelikefalin-KOR-Gi complex. **c,** Flow chart of cryo-EM data processing using cryoSPARC. **d,** Local resolution map to the surface of the difelikefalin-KOR-Gi complex and difelikefailn. **e,** The "Gold-standard" Fourier shell correlation (FSC) curve indicates that the

resolution of the global electron density map of difelikefalin-KOR-Gi complex is 2.43 Å. **f,** Cryo-EM density maps of difelikefalin and all transmembrane helices of KOR.

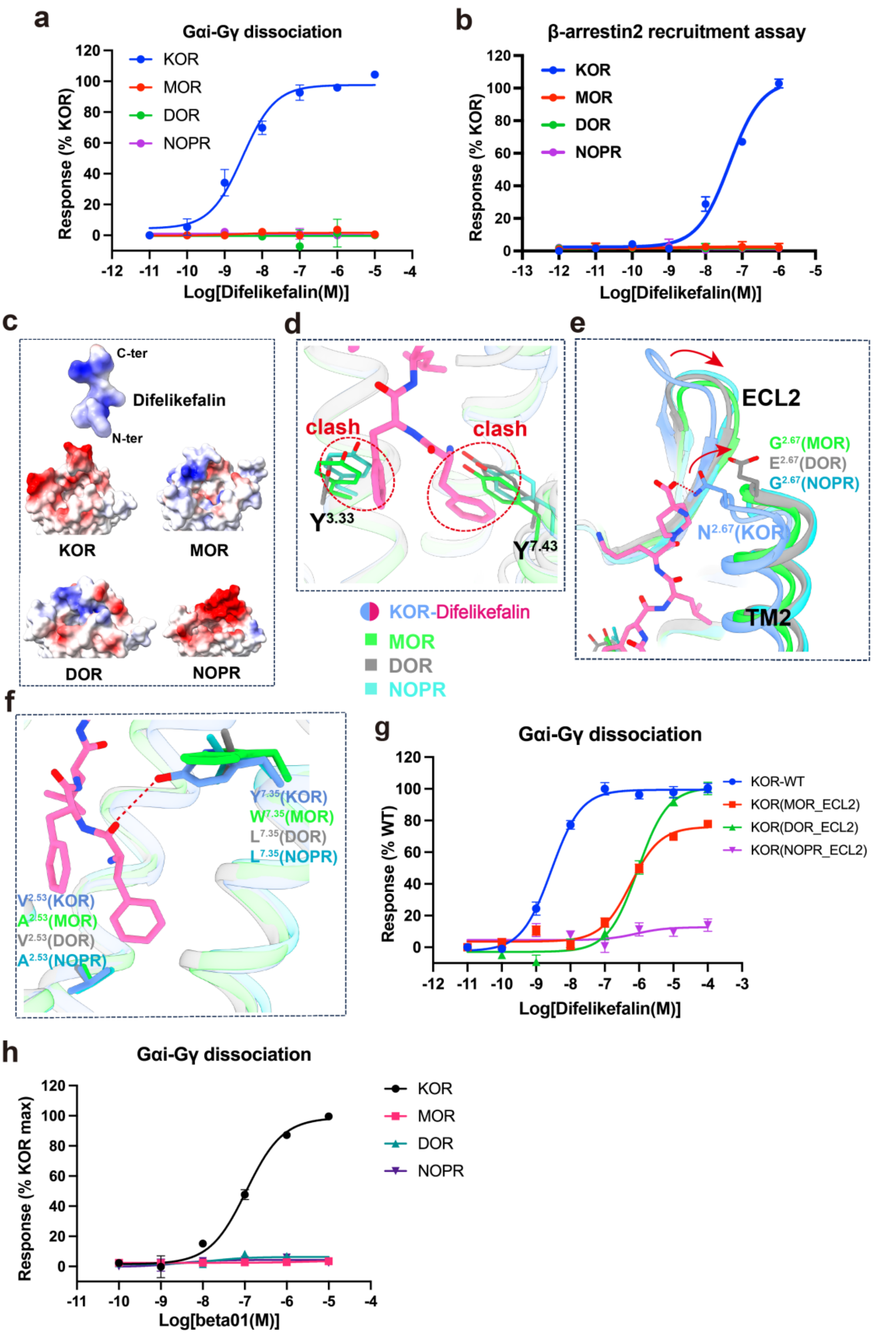

Supplementary Fig. 2 | Selective recognition of KOR by difelikefalin. a, $G\alpha_i$-Gγ dissociation of

four opioid receptors induced by difelikefalin. Data are expressed as mean ± S.E.M. of three independent experiments conducted in triplicate. Source data are provided as a Source Data file. **b,** β-arrestin2 recruitment of four opioid receptors induced by difelikefalin. Data are expressed as mean ± S.E.M. of three independent experiments conducted in triplicate. Source data are provided as a Source Data file. **c,** Electrostatic surface representation of difelikefalin, beta01 and extracellular parts of opioid receptors. **d,** Steric clash of $Y^{7.43}$ and $Y^{3.33}$ in MOR/DOR/NOPR with difelikefalin. Color usage: difelikefalin is colored in hot pink and orange; KOR in purple; MOR in lime; DOR in grey; NOPR in light green. **e,** The detailed interactions of difelikefalin with the extracellular segment of TM2 and ECL2 of the opioid receptors. **f,** Detailed interactions between difelikefalin and opioid receptors at the position of $TM^{7.35}$ and $TM^{2.53}$. Indicating that the conformation of difelikefalin is favorable for binding to KOR, but unfavorable for binding to other three opioid receptors. Color usage: difelikefalin is colored in hot pink, respectively; KOR in purple; MOR in lime; DOR in grey; NOPR in light green. **g,** Effects of KOR mutations with corresponding ECL2 of MOR/DOR/NOPR on G protein signaling induced by difelikefalin. Data are expressed as mean ± S.E.M. of three independent experiments conducted in triplicate. Source data are provided as a Source Data file. **h,** Dose-dependent response curves of four opioid receptors activated by beta01 in $G\alpha_i$-$G\gamma$ dissociation assay, indicating beta01 can selectively activate KOR but not MOR, DOR or NOPR. Data are expressed as mean ± S.E.M. of three independent experiments conducted in triplicate. Source data are provided as a Source Data file.

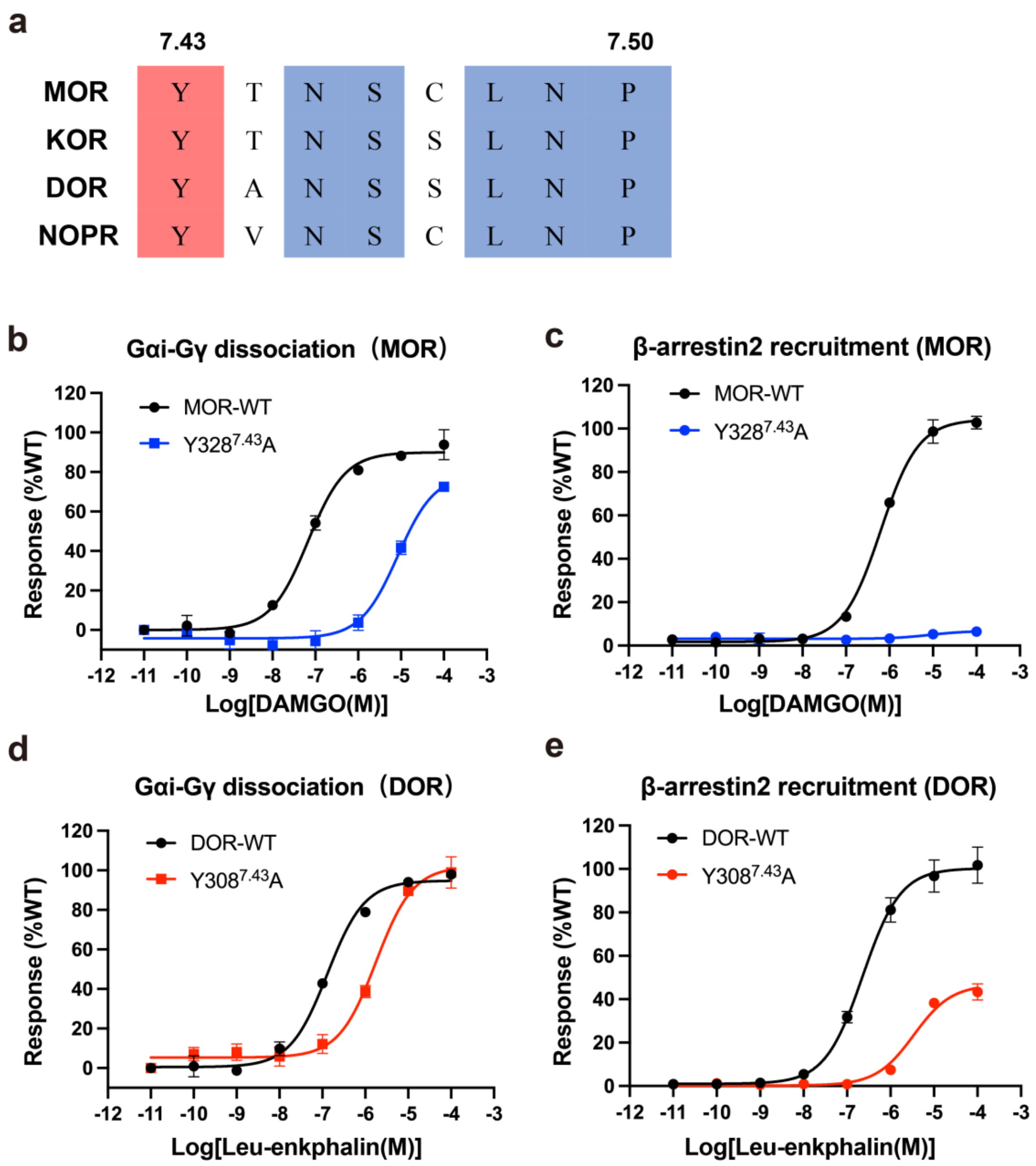

Supplementary Fig. 3 | Y$^{7.43}$ is highly conserved in the opioid family and is crucial for the signal transduction of β-arrestin. a, Sequence alignment of partial TM7 of opioid receptors. **b, c,** Effect of Y328$^{7.43}$A mutant in Gi activation (**b**) and β-arrestin2 recruitment (**c**) of MOR. Data are expressed as mean ± S.E.M. of three independent experiments conducted in triplicate. Source data are provided as a Source Data file. **d, e,** Effect of Y308$^{7.43}$A mutant in Gi activation (**d**) and β-arrestin2 recruitment (**e**) of DOR. Data are expressed as mean ± S.E.M. of three independent experiments conducted in triplicate. Source data are provided as a Source Data file.

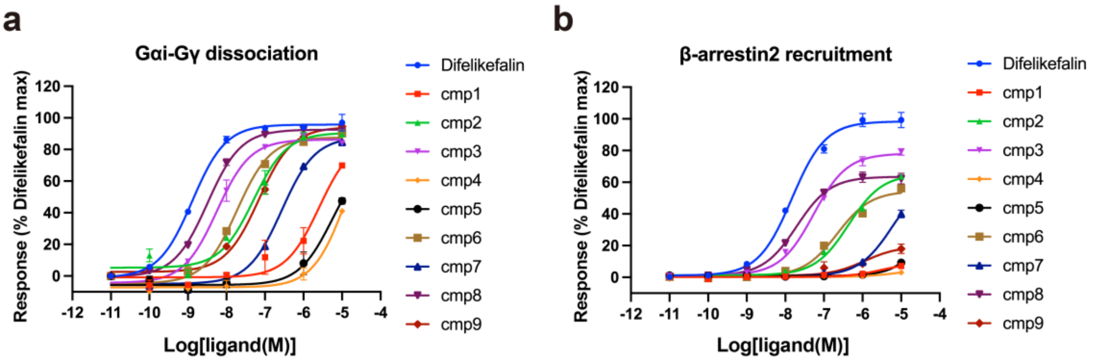

Supplementary Fig. 4 | Dose-response curves for Gα$_i$-Gγ dissociation (a) and β-arrestin2 recruitment (b) of KOR induced by difelikefalin derivatives modified at D-Phe1. Data are expressed as mean ± S.E.M. of three independent experiments conducted in triplicate. Source data are provided as a Source Data file.

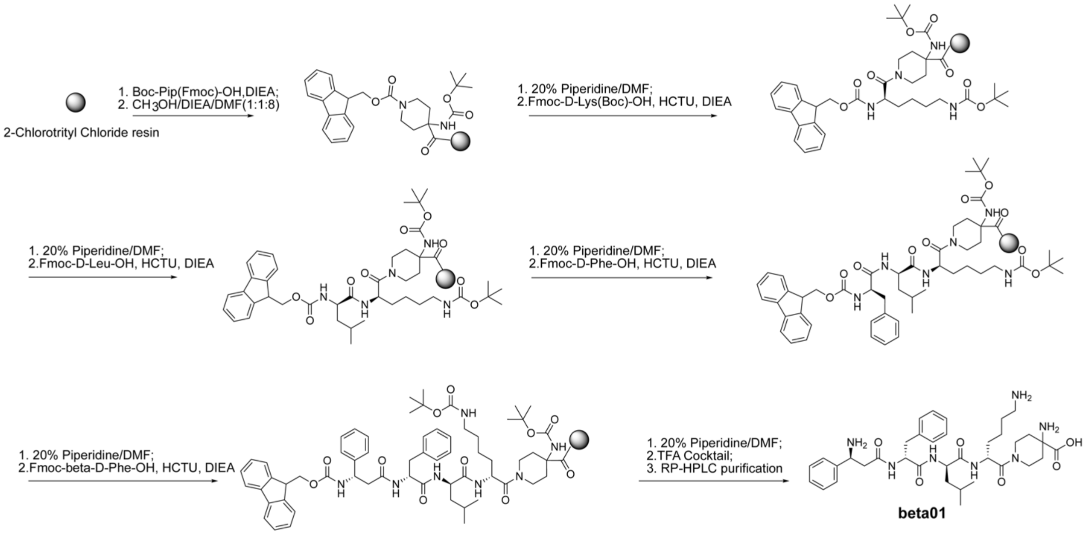

Supplementary Fig. 5 | Representative chemical synthesis of ligands. Bate01 used as an example.

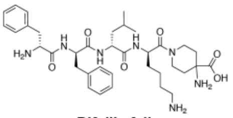
Difelikefalin
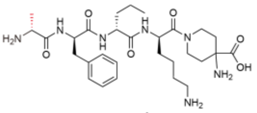
cmp1
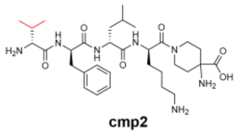
cmp2
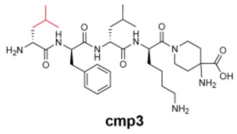
cmp3
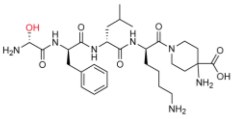
cmp4
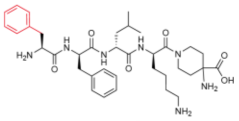
cmp5
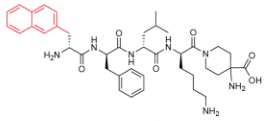
cmp6
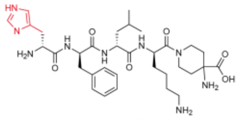
cmp7
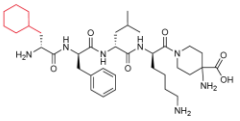
cmp8
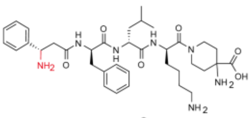
cmp9
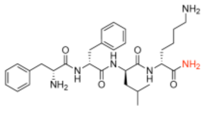
cmp10
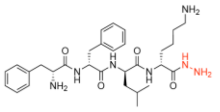
cmp11
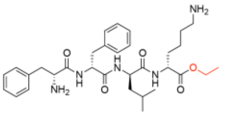
cmp12
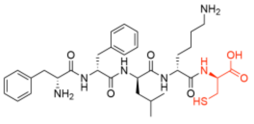
cmp13
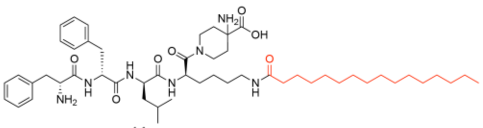
cmp14
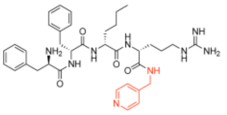
cmp15
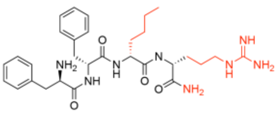
cmp16
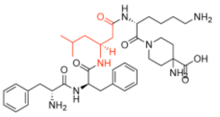
cmp17
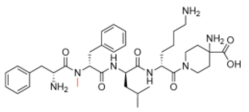
cmp18
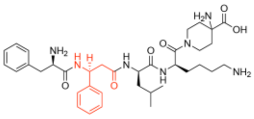
cmp19
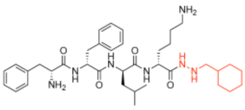
cmp20
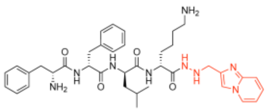
cmp21
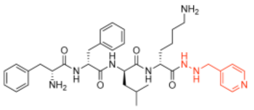
cmp22
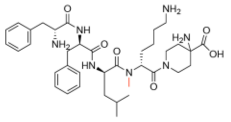
cmp23
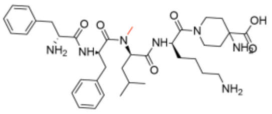
cmp24
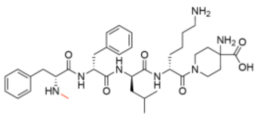
cmp25
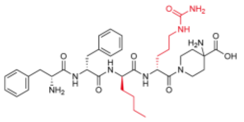
cmp27
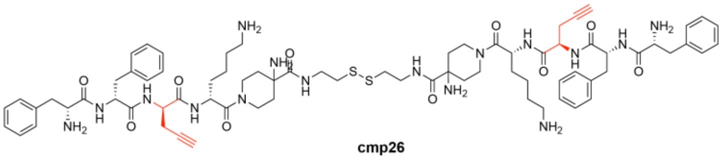
cmp26
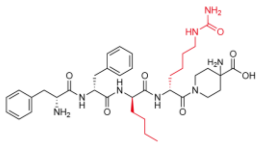
cmp28
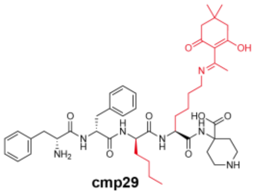
cmp29
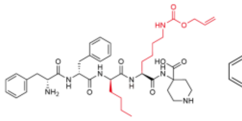
cmp30
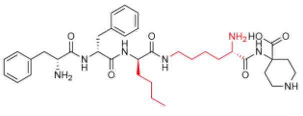
cmp31
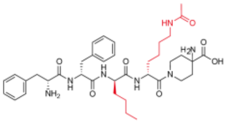
cmp32

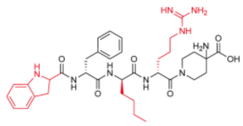
cmp33

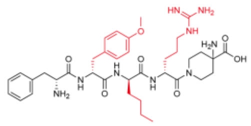
cmp34

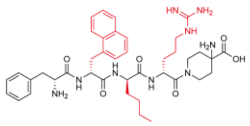
cmp35

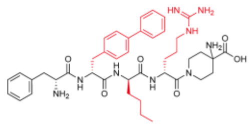
cmp36

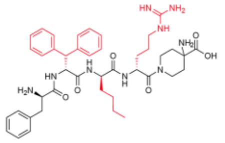
cmp37

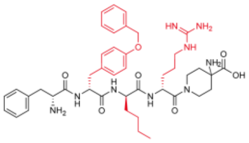
cmp38

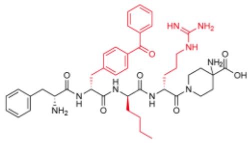
cmp39

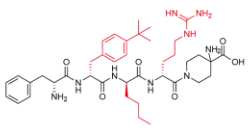
cmp40

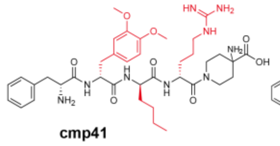
cmp41

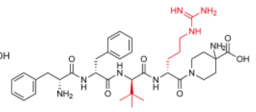
cmp42

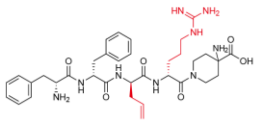
cmp43

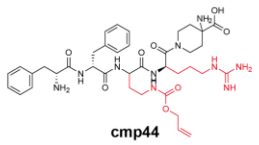
cmp44

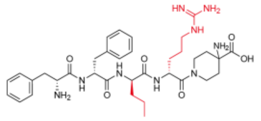
cmp45

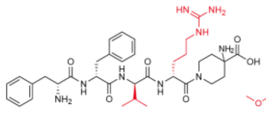
cmp46

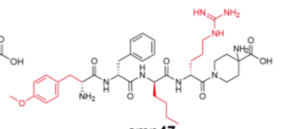
cmp47

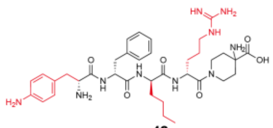
cmp48

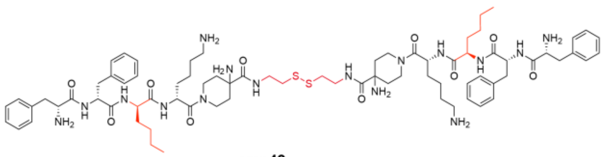
cmp49

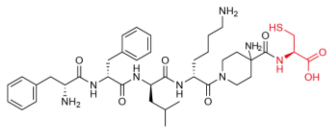
cmp50

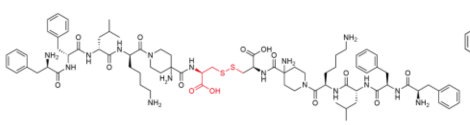
cmp51

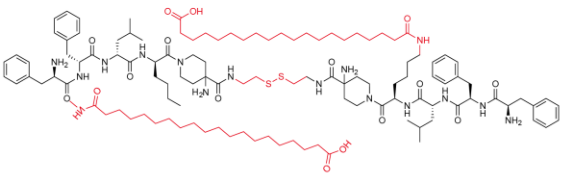
cmp52

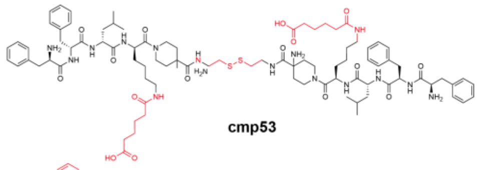
cmp53

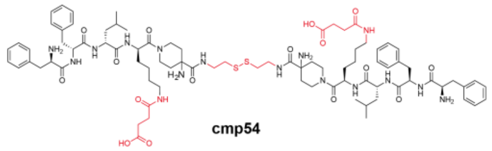
cmp54

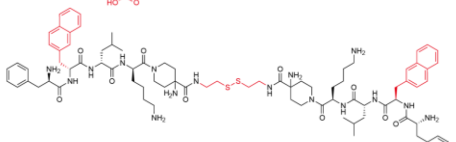
cmp55

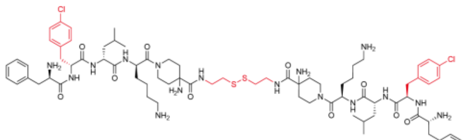
cmp56

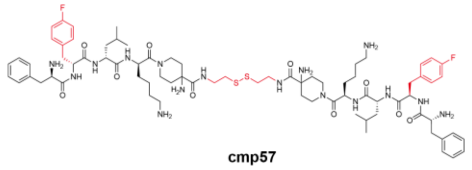
cmp57

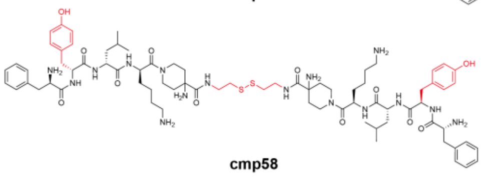
cmp58

Supplementary Fig. 6 | Chemical structures of difelikefalin derivatives.

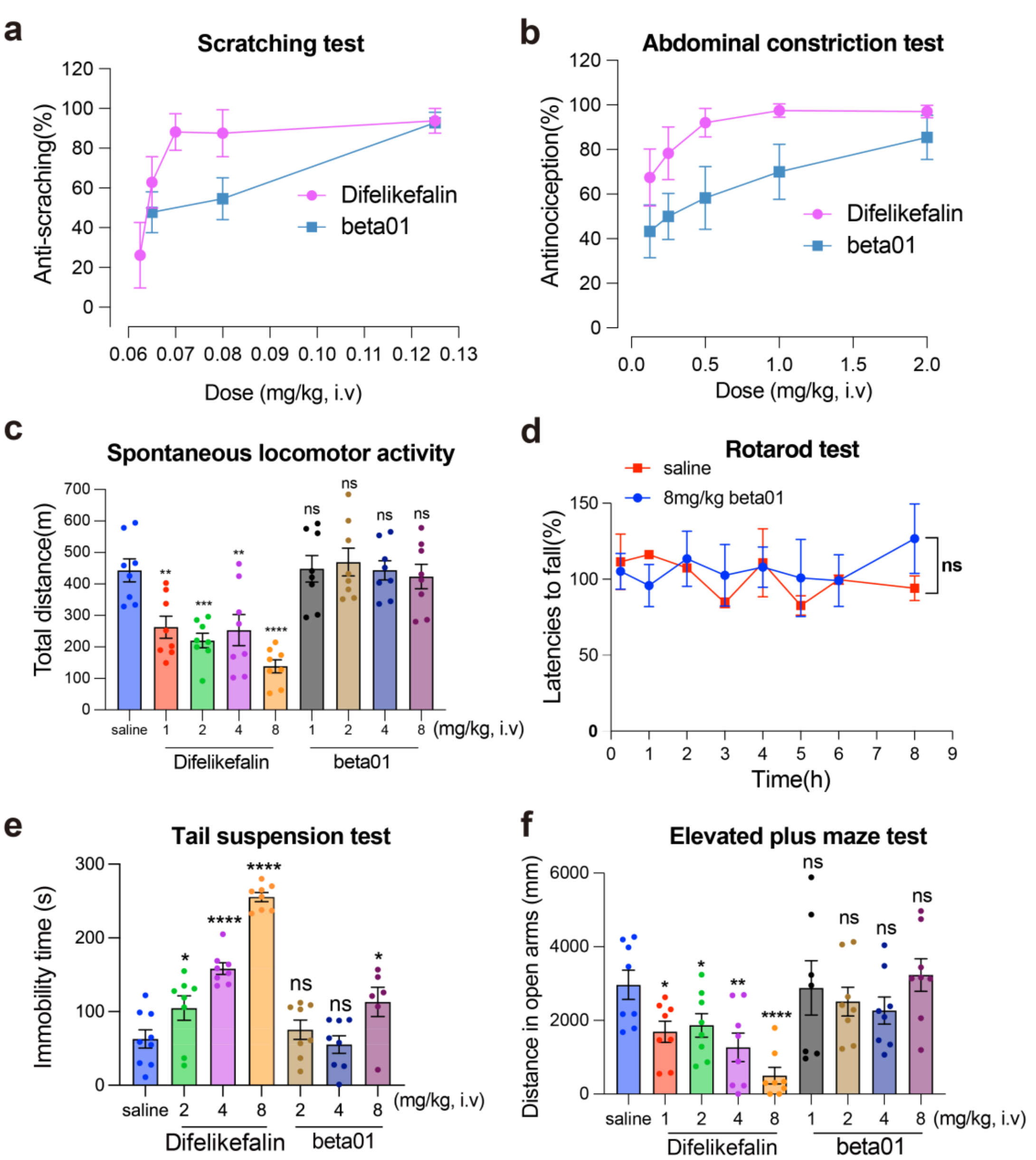

Supplementary Fig. 7 | Behavioral studies of difelikeflain and beta01 in mouse models.
a, Antipruritic effects in response to administration of chloroquine diphosphate. Data are presented as the mean ± S.E.M. from 8 mice/data point. Source data are provided as a Source Data file. **b,** Antinociceptive effects in the acid writhing test. Data are presented as the mean ± S.E.M. from 8 mice/data point. Source data are provided as a Source Data file. **c,** Effect of difelikefalin and beta01 on spontaneous activities in mice. Data are presented as the mean ± S.E.M. from 8 mice/data point. All statistical tests used were two-tailed t-tests. * $P < 0.05$, **$P < 0.01$, ***$P < 0.001$, ****$P < 0.0001$; ns, not significant $p > 0.05$. Source data and exact *p* value are provided as a Source Data file. **d,** Time course for the sedation effect of beta01 at the dose of 8mg/kg weight in the rotarod test. Each value represents the mean ± SEM from at least six mice. ns means no significant difference. All statistical tests used were two-tailed Student's t-tests. Source data are provided as a Source Data file. **e,** Immobility time statistics of mice treated with different doses of difelikeflain and beta01 in

the tail suspension test. Data are presented as the mean ± SEM from at least 6 mice/data point. （saline, n = 9; 2 mg/kg difelikefalin, n = 8; 4 mg/kg difelikefalin, n = 8; 8 mg/kg difelikefalin, n = 8; 2 mg/kg beta01, n = 8; 4 mg/kg beta01, n = 8; 8 mg/kg beta01, n = 6. All statistical tests used were two-tailed t-tests）. * $P < 0.05$, **$P < 0.01$, ***$P < 0.001$, ****$P < 0.0001$; ns, not significant $p > 0.05$. Source data and exact *p* value are provided as a Source Data file. **f,** The distance traveled within the open arms of mice during the elevated plus maze test. Data are presented as the mean ± S.E.M. from at least 6 mice/data point. （saline, n = 8; 1 mg/kg difelikefalin, n = 8; 2 mg/kg difelikefalin, n = 8; 4 mg/kg difelikefalin, n = 8; 8 mg/kg difelikefalin, n = 8; 1 mg/kg beta01, n = 7; 2 mg/kg beta01, n = 8; 4 mg/kg beta01, n = 8; 8 mg/kg beta01, n = 8. All statistical tests used were two-tailed t-tests）. * $P < 0.05$, **$P < 0.01$, ***$P < 0.001$, ****$P < 0.0001$; ns, not significant $p > 0.05$. Source data and exact *p* value are provided as a Source Data file.

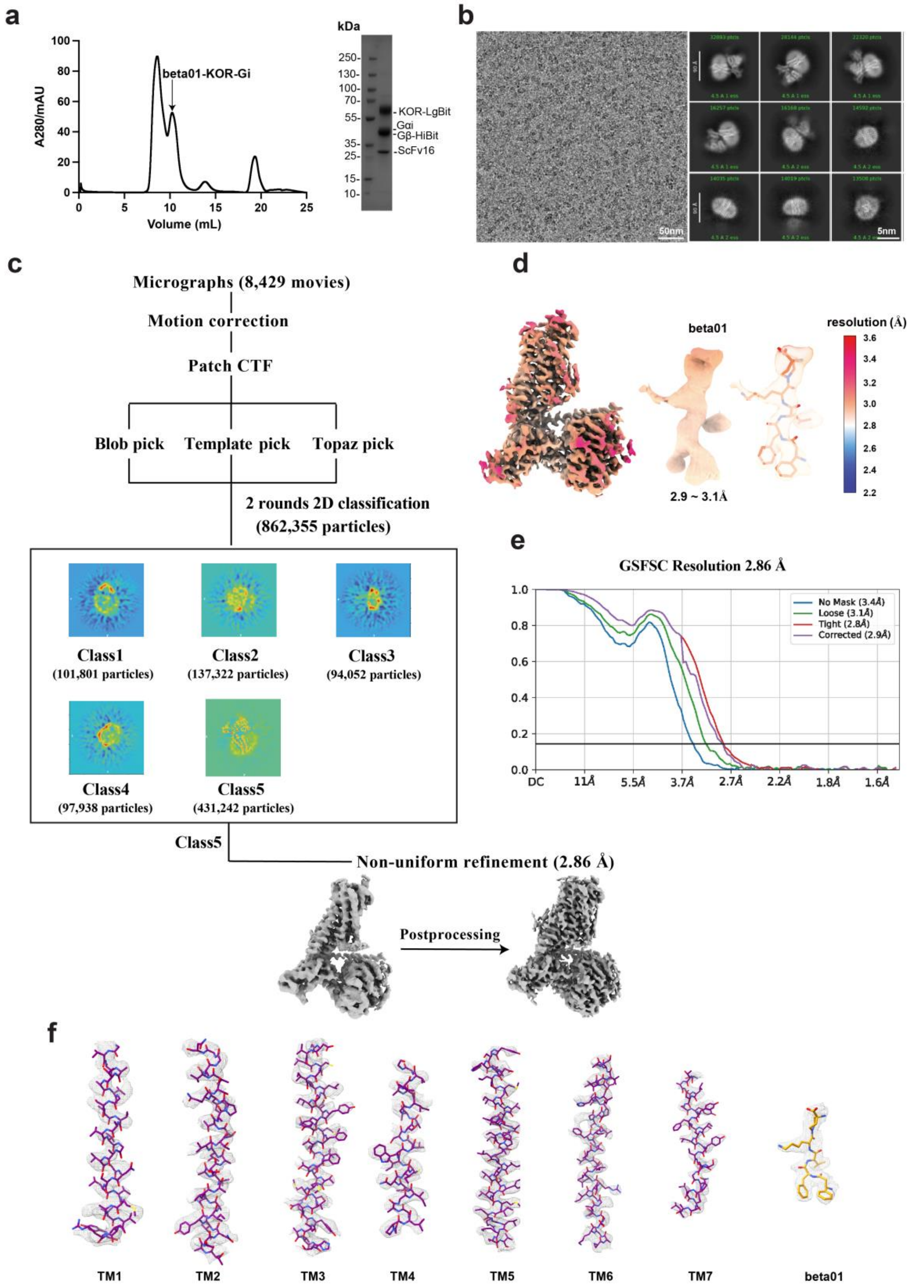

a
beta01-KOR-Gi
A280/mAU
100
80
60
40
20
0
0
5
10
15
20
25
Volume (mL)
kDa
250-
130-
100-
70-
55-
35-
25-
15-
10-
KOR-LgBit
Gαi
Gβ-HiBit
ScFv16
b
50nm
5nm
c
Micrographs (8,429 movies)
Motion correction
Patch CTF
Blob pick
Template pick
Topaz pick
2 rounds 2D classification
(862,355 particles)
Class1
(101,801 particles)
Class2
(137,322 particles)
Class3
(94,052 particles)
Class4
(97,938 particles)
Class5
(431,242 particles)
Class5
Non-uniform refinement (2.86 Å)
Postprocessing
d
beta01
resolution (Å)
3.6
3.4
3.2
3.0
2.8
2.6
2.4
2.2
2.9 ~ 3.1Å
e
GSFSC Resolution 2.86 Å
1.0
0.8
0.6
0.4
0.2
0.0
DC
11Å
5.5Å
3.7Å
2.7Å
2.2Å
1.8Å
1.6Å
No Mask (3.4Å)
Loose (3.1Å)
Tight (2.8Å)
Corrected (2.9Å)
f
TM1
TM2
TM3
TM4
TM5
TM6
TM7
beta01

Supplementary Fig. 8 | Purification and cryo-EM data processing of beta01 bound KOR-Gi complex. a, Size-exclusion chromatography profile and SDS-PAGE analysis of beta01-KOR-Gi complex. **b,** Representative micrograph after motion correction and dose weighting and 2D class averages of beta01-KOR-Gi complex. **c,** Flow chart of cryo-EM data processing using cryoSPARC. **d,** Local resolution map to the surface of the beta01-KOR-Gi complex and beta01. **e,** The "Gold-standard" Fourier shell correlation (FSC) curve indicates that the resolution of the global electron density map of beta01-KOR-Gi complex is 2.86 Å. **f** Cryo-EM density maps of beta01 and all transmembrane helices of KOR.

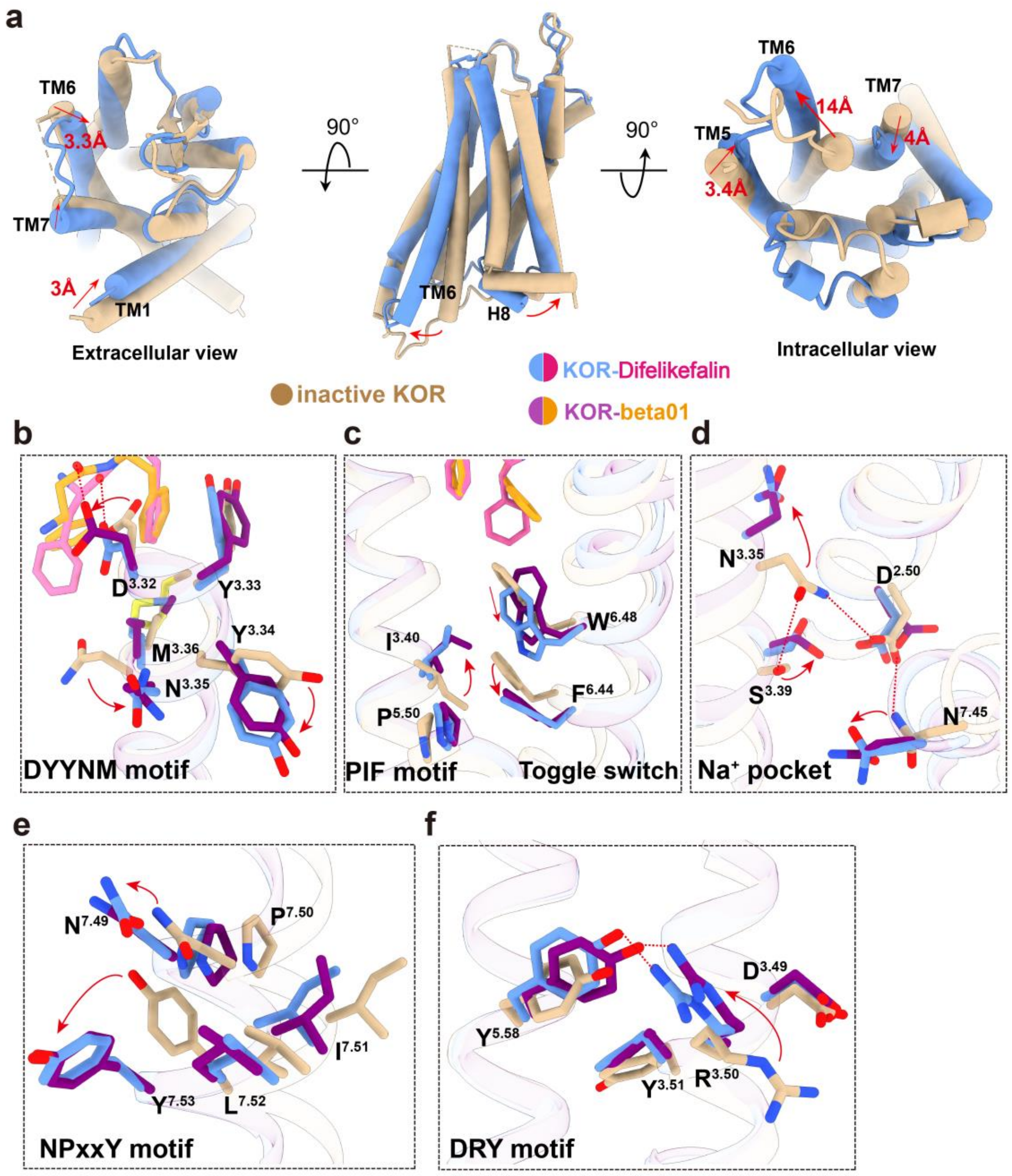

Supplementary Fig. 9 | The molecular mechanisms underlying the activation of the KOR by

difelikefalin and beta01. a, Structure alignment of difelikefalin activated KOR and antagonist (JDTic) bound inactive KOR. The conformational changes of difelikefalin activated KOR compared with inactive KOR are marked with red arrows. **b-f,** Conformational differences of important residues and motifs in KOR activation. Color usage: difelikefalin and beta01 are colored in hot pink and orange, respectively; Difelikefalin bound KOR, cornflower blue; beta01 bound KOR, purple; inactive KOR, tan.

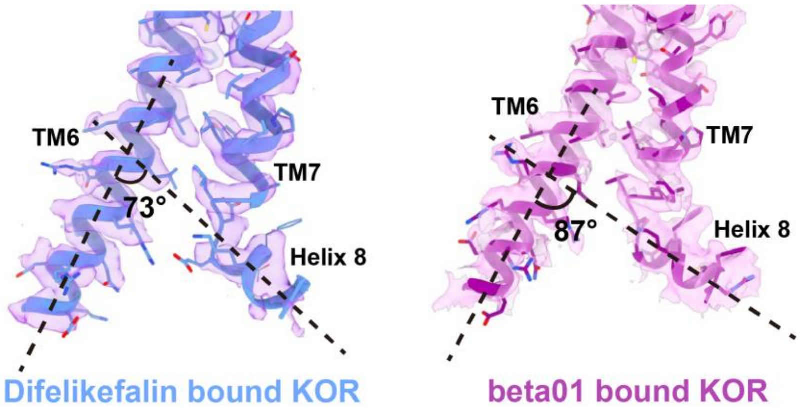

Supplementary Fig. 10 | Cryo-EM density maps of the intracellular portions of TM6, TM7, and Helix8 of KOR, in the presence of difelikefalin and beta01.

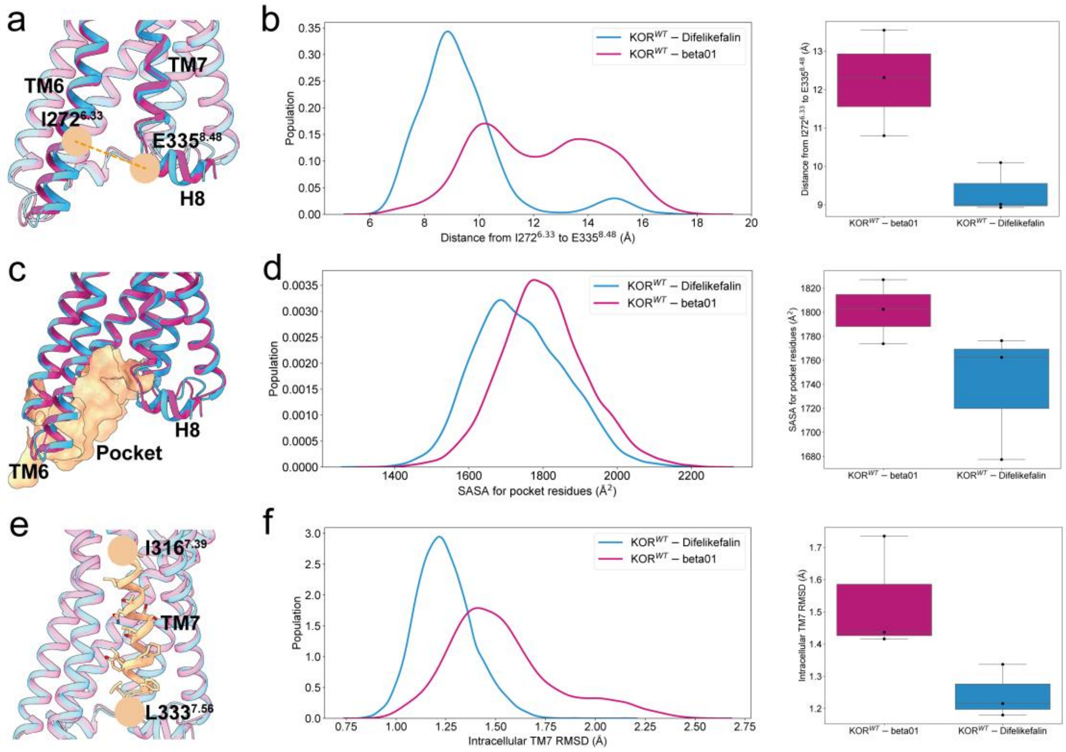

Supplementary Fig. 11 | Conformational alterations for the intracellular domain induced by

difelikefalin and beta01 in MD simulations. a, The TM6–H8 conformation in the cryo-EM structure, with the Cα atoms of I272$^{6.33}$ and E335$^{8.48}$ shown as wheat-colored spheres. **b,** The distribution of the Cα distance between I272$^{6.33}$ and E335$^{8.48}$ for the KOR–difelikefalin and KOR–beta01 systems in MD, illustrated using a KDE plot (left) to show the probability density and a boxplot (right) to summarize statistical variation between the two systems. **c,** The TM6–H8 conformation in the cryo-EM structure, with the wheat-colored surface indicating the solvent-accessible surface area (SASA) of G-protein binding pocket residues. **d,** The distribution of SASA values for pocket residues in the KOR–difelikefalin and KOR–beta01 systems in MD, illustrated using a KDE plot (left) and a boxplot (right). **e,** The TM6–H8 conformation in the cryo-EM structure, with the heavy atoms of residues I316$^{7.39}$–L333$^{7.56}$ of KOR highlighted in wheat. **f,** The distribution of heavy-atom RMSD for intracellular TM7 in the KOR–difelikefalin and KOR–beta01 systems in MD, illustrated using a KDE plot (left) and a boxplot (right).

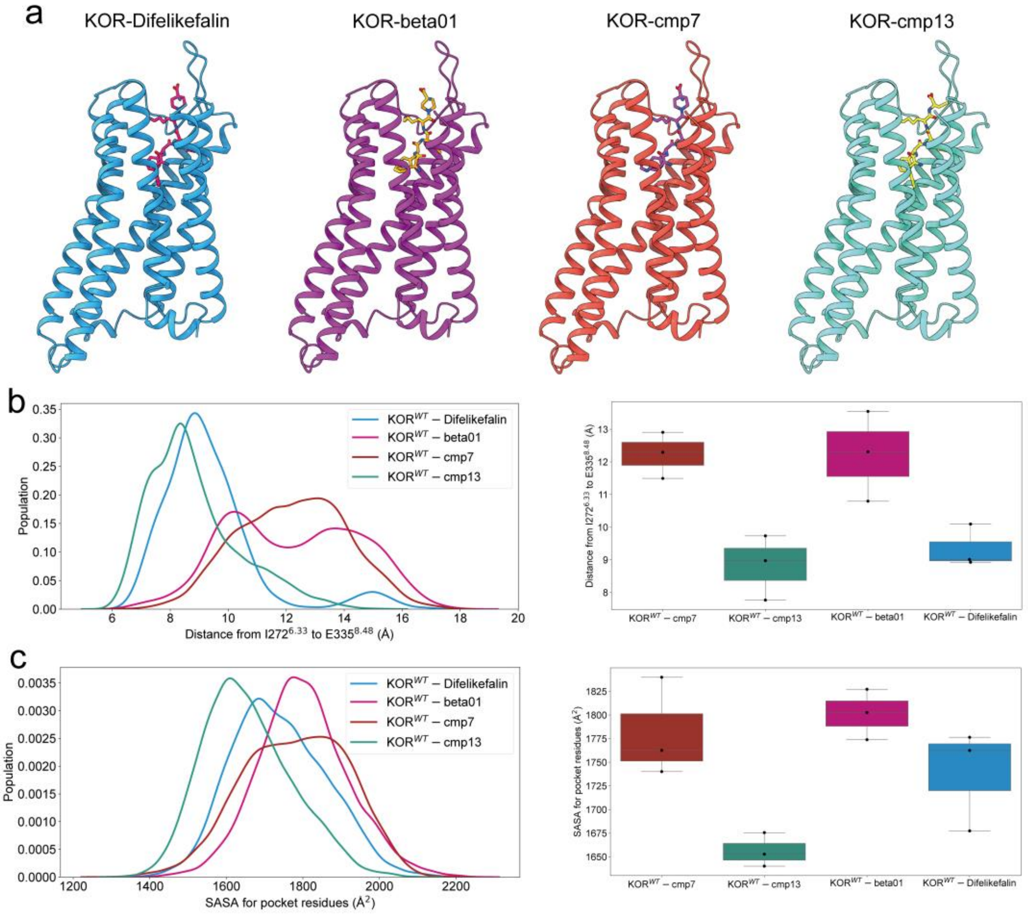

Supplementary Fig. 12 | Conformational alterations for the intracellular domain of KOR induced by difelikefalin, beta01, cmp7 and cmp13. a, The initial structures of simulation systems KOR-Difelikefalin, KOR-beta01, KOR-cmp7 and KOR-cmp13. **b,** The distribution of the Cα distance between I272$^{6.33}$ and E335$^{8.48}$ for the KOR–difelikefalin, KOR–beta01, KOR-cmp7 and KOR-cmp13 systems in MD, illustrated using a KDE plot (left) to show the probability density and a boxplot (right) to summarize statistical variation between the two systems. **c,** The distribution of SASA values for pocket residues in the KOR–difelikefalin, KOR–beta01, KOR-cmp7 and KOR-cmp13 systems in MD, illustrated using a KDE plot (left) and a boxplot (right).

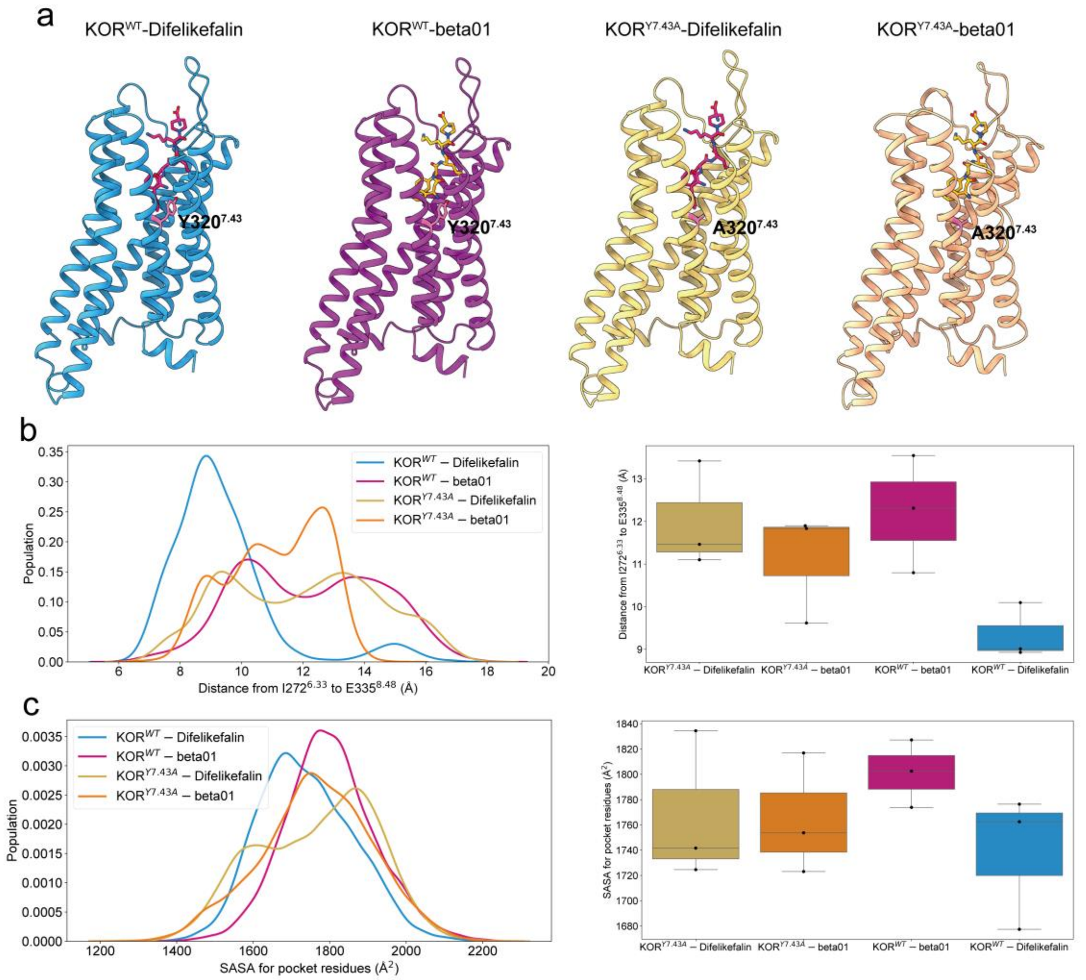

Supplementary Fig. 13 | Conformational alterations for the intracellular domain of KOR^{WT} and KOR$^{Y7.43A}$ induced by difelikefalin and beta01 in molecular dynamics. a, The initial structures of simulation systems KOR^{WT}-difelikefalin, KOR^{WT}-beta01, KOR$^{Y7.43A}$-difelikefalin and KOR$^{Y7.43A}$-beta01. **b,** The distribution of the Cα distance between I272$^{6.33}$ and E335$^{8.48}$ for the KOR^{WT}-difelikefalin, KOR^{WT}-beta01, KOR$^{Y7.43A}$-difelikefalin and KOR$^{Y7.43A}$-beta01 systems in MD, illustrated using a KDE plot (left) to show the probability density and a boxplot (right) to summarize statistical variation between the two systems. **c,** The distribution of SASA values for pocket residues in the KOR^{WT}-difelikefalin, KOR^{WT}-beta01, KOR$^{Y7.43A}$-difelikefalin and KOR$^{Y7.43A}$-beta01 systems in MD, illustrated using a KDE plot (left) and a boxplot (right).

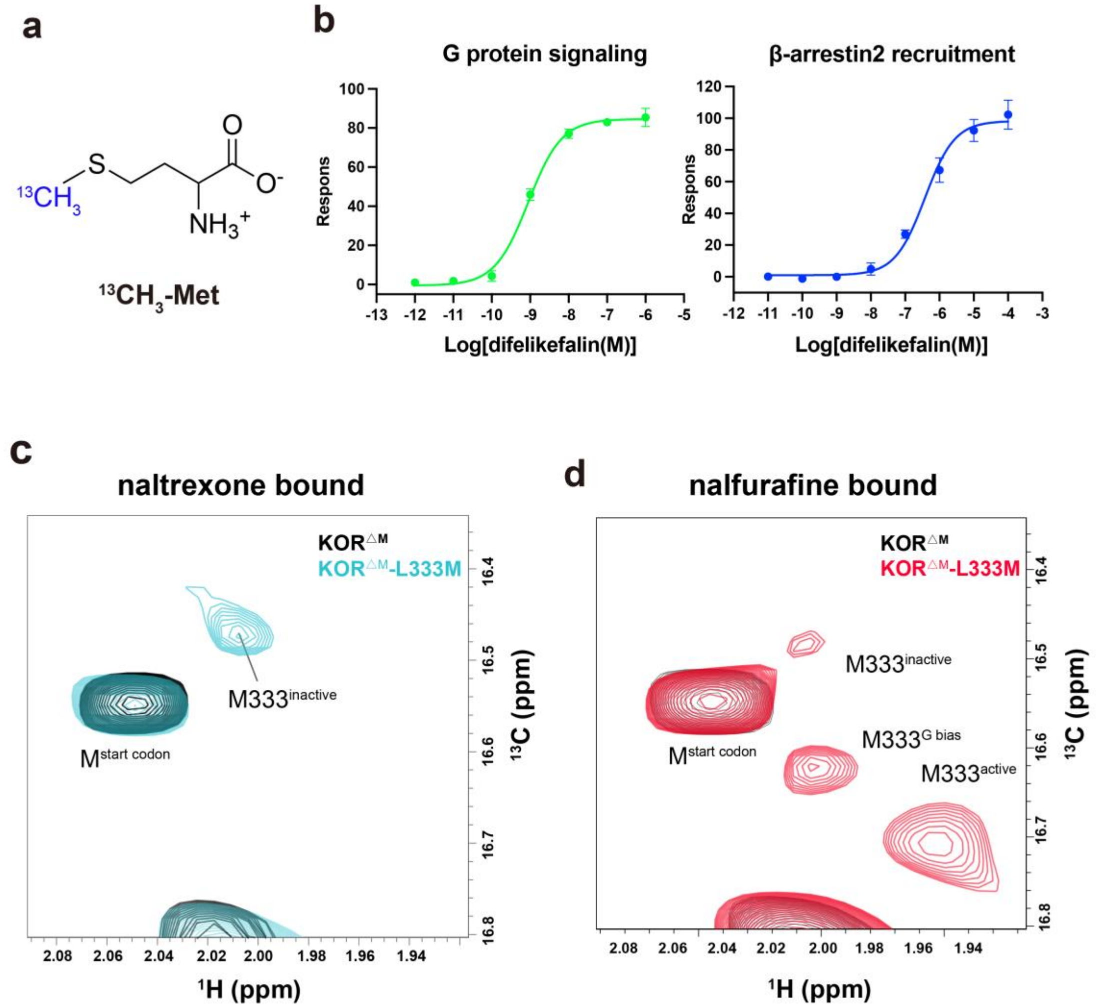

Supplementary Fig. 14 | ^{1}H-^{13}C SOFAST-HMQC spectra of [methyl-^{13}C-Met] KOR. a, The chemical structural formula of ^{13}CH3-methionine. **b,** Functional validation of the construct used in NMR experiments. Data shown are means ± S.E.M. from three independent experiments performed in technical duplicates. Source data are provided as a Source Data file. **c, d,** ^{1}H-^{13}C HMQC spectra of [methyl-^{13}C-Met] KOR-0M or 1M-L333M bound to naltrexone (**c**) or nalfurafine (**d**).

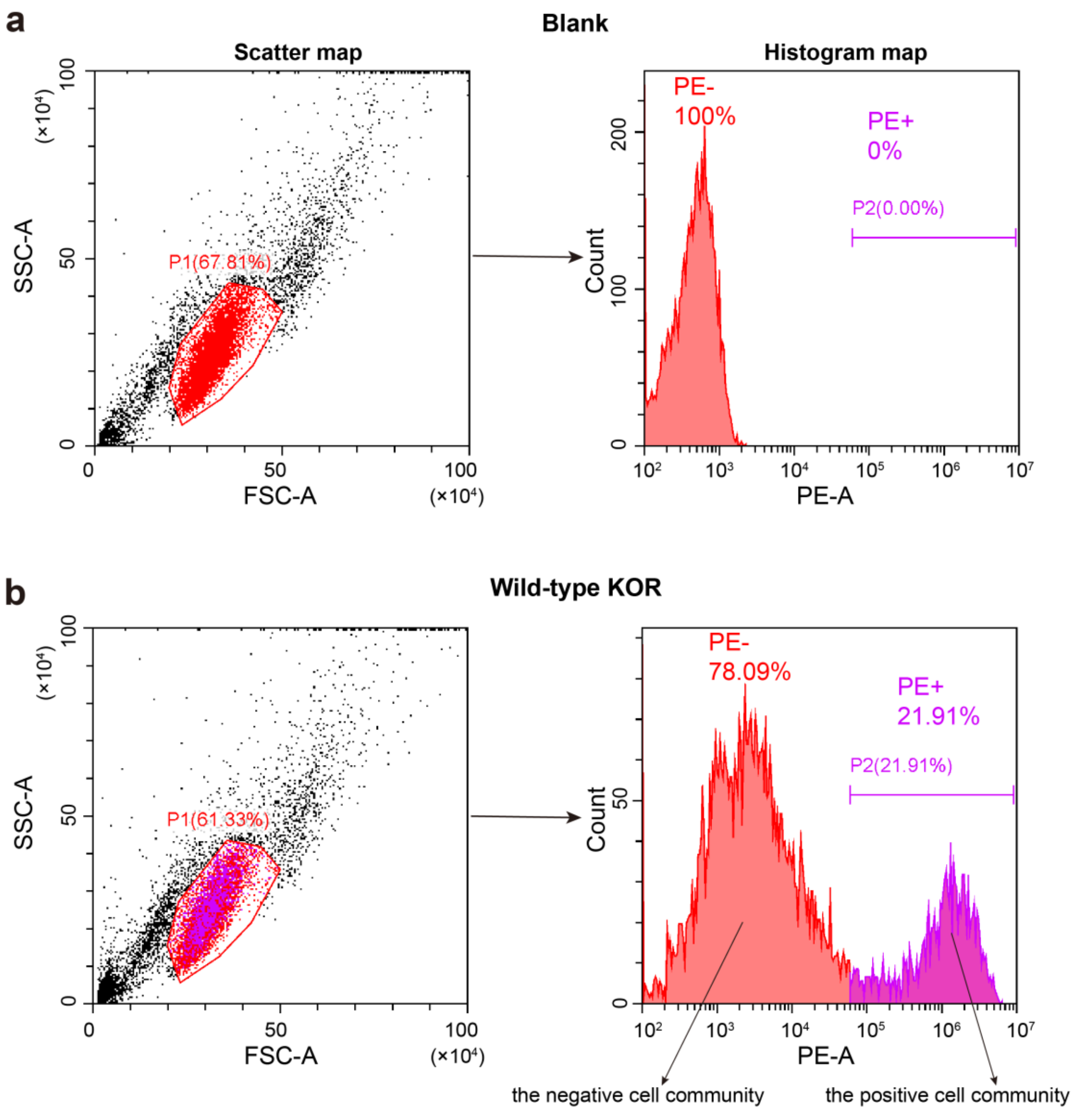

Supplementary Fig. 15 | Gating strategy of cell surface expression assay. Circling a gate P1 in the scatter map (black circle). The cells displayed in the histogram map are all those within gate P1 from the scatter map. Fluorescence signal intensity (PE) is presented by the histogram map. Using the Blank sample as the reference value for background fluorescence signal **(a)**, the fluorescence signal histogram map is divided into two regions. The left region represents the negative cell population (PE$^-$), while the right region represents the positive cell population (PE$^+$). The expression level of the cell surface wild-type (WT) KOR **(b)** can be calculated as follows: (M(PE$^+$)-M(PE$^-$)) × (PE$^+$% Parent). The expression level of the KOR mutant is calculated similarly to the WT receptor and then normalized to the WT to determine the relative expression value.

Supplementary Table 1 | Cryo-EM data collection, refinement and validation statistics

**Data Collection**		
**Protein**	difelikefalin-KOR-Gi	beta01-KOR-Gi
**Voltage (kV)**	300	300
**Detector**	K3	Falcon4
**Pixel size (Å)**	0.824	0.73
**Defocus range (μm)**	-1.0 ~ -3.0	-1.0 ~ -3.0
**Electron dose (e^-/Å^2)**	50	50
**Frames per image**	36	36
**Exposure time (s)**	2.5	2.5
**3D reconstruction**		
**Final Particle number**	623,181	431,242
**Symmetry**	C1	C1
**Overall resolution (Å)**	2.43	2.86
**Model refinement**		
**Model composition**		
**Chains**	7	6
**Ligands**	difelikefalin	beta01
**Non-hydrogen atoms**	9,067	8,926
**Protein residues**	1,137	1,135
**Bonds (RMSD)**		
**Length (Å)**	0.020	0.006
**Angles (°)**	1.131	0.991
**Ramachandran plot (%)**		
**Outliers**	0.00	0.00
**Allowed**	1.69	2.14
**Favored**	98.31	97.86
**Rotamer outliers (%)**	0.41	0.92
**MolProbity score**	1.25	1.43
**Clash score**	4.75	7.27

Supplementary Table 2 | Difelikefalin-induced G_i activation and β-arrestin2 recruitment of opioid receptors or mutants

	G protein signaling		β-arrestin2 recruitment		Expression (% WT)
	pEC50	Emax (% KOR-	pEC50	Emax (% KOR-	
KOR-WT	8.58±0.01	100	7.52±0.01	100	100
V108$^{2.53}$A	6.59±0.33	95.78±5.87	6.53±0.17	53.81±1.64	90.42±1.93
T111$^{2.56}$A	8.10±0.01	83.69±3.01	6.97±0.01	58.13±0.11	86.40±5.26
Q115$^{2.60}$A	6.16±0.76	61.77±5.18	N.D	N.D	84.99±8.34
V134$^{3.28}$A	7.17±0.15	84.95±8.37	7.06±0.11	67.57±4.31	90.30±7.64
I135$^{3.29}$A	5.42±0.35	64.79±2.40	6.08±0.19	36.00±1.59	74.55±3.27
D138$^{3.32}$A	N.D	N.D	N.D	N.D	95.89±6.23
Y139$^{3.33}$A	N.D	N.D	6.34±0.15	58.12±3.54	49.86±3.32
M142$^{3.36}$A	8.37±0.36	90.75±12.45	6.96±0.09	94.76±0.81	91.88±11.38
E209^{ECL2}A	8.72±0.11	103.26±2.85	7.76±0.17	107.40±2.08	87.75±2.66
S211^{ECL2}A	8.56±0.09	98.32±5.62	7.82±0.16	103.35±1.53	89.81±7.29
D223$^{5.35}$A	8.09±0.07	100.80±0.13	7.08±0.13	109.50±2.37	60.05±2.23
K227$^{5.39}$A	8.73±0.11	97.45±7.05	7.89±3.41	64.73±4.01	88.87±7.86
V230$^{5.42}$A	8.49±0.20	107.59±11.13	6.54±0.07	72.60±6.47	79.36±5.05
W287$^{6.48}$A	6.69±0.26	63.12±8.44	6.77±0.19	41.43±0.51	72.29±7.40
I294$^{6.55}$A	8.43±0.11	90.25±1.97	7.32±0.02	104.09±1.62	76.71±9.95
I316$^{7.39}$A	9.36±0.19	90.64±3.62	7.27±0.11	95.98±1.46	93.51±8.24
Y320$^{7.43}$A	7.37±0.48	84.64±2.57	4.63±0.17	67.28±5.00	72.78±9.42
KOR(MOR_ECL2)	6.39±0.01	79.58±3.41	/	/	66.40±5.93
KOR(DOR_ECL2)	5.95±0.02	102.58±0.22	/	/	82.38±6.17
KOR(NOPR_ECL2)	N.D	N.D	/	/	72.13±7.50
MOR-WT	N.D	N.D	N.D	N.D	100
DOR-WT	N.D	N.D	N.D	N.D	100
NOPR-WT	N.D	N.D	N.D	N.D	100
KOR$^{\triangle M}$	8.50±0.10	94.9±1.92	/	/	89.14±4.26
KOR$^{\triangle M}$-L333M	8.98±0.12	88.1±2.07	/	/	93.45±2.60

'N.D' means Not Detectable or cannot be established over the tested concentration range.

' /' means no measurement.

Supplementary Table 3 | Ligands-induced G_i activation and β-arrestin2 recruitment of opioid receptors or mutants

Ligands	Receptors	G protein signaling		β-arrestin2 recruitment		Expression (%WT)
		pEC50	Emax (% WT)	pEC50	Emax (% WT)	
beta01	KOR-WT	7.10±0.01	100	5.89±0.06	100	100
	MOR-WT	N.D	N.D	/	/	100
	DOR-WT	N.D	N.D	/	/	100
	NOPR-WT	N.D	N.D	/	/	100
DAMGO	MOR-WT	7.17±0.05	100	6.21±0.53	100	100
	MOR-Y328$^{7.43}$A	5.08±0.42	88.47±4.39	N.D	N.D	104.83±6.82
Leu-enkphalin	DOR-WT	6.88±0.16	100	6.64±0.13	100	100
	DOR-Y308$^{7.43}$A	5.75±0.53	93.67±3.18	5.46±0.37	53.67±3.25	92.68±6.65

'N.D' means not detected or cannot be established over the tested concentration range.

' /' means no measurement.

Supplementary Table 4 | Functional test results of Difelikefalin derivatives

Ligands	G protein signaling		β-arrestin2 recruitment		Bias factor*
	pEC50	Emax (% difelikefalin)	pEC50	Emax (% difelikefalin)	
difelikefalin	8.87±0.01	100	7.82±0.01	100	0
cmp1	5.56±0.03	93.12±1.51	N.D	N.D	N.D
cmp2	7.24±0.03	97.05±0.73	6.36±0.03	70.05±0.61	-0.03±0.03
cmp3	8.24±0.01	89.53±0.16	7.27±0.01	81.19±0.99	-0.03±0.02
cmp4	5.05±0.01	84.72±0.97	N.D	N.D	N.D
cmp5	5.29±0.02	75.89±1.85	N.D	N.D	N.D
cmp6	7.77±0.01	87.69±0.47	6.66±0.01	60.81±0.60	0.15±0.68
cmp7	6.52±0.01	94.00±0.36	5.22±0.03	68.99±2.16	0.38±0.03
cmp8	8.49±0.01	95.83±0.42	7.70±0.01	68.62±1.21	-0.12±0.01
cmp9	7.11±0.03	100±0.55	5.89±0.06	31.86±1.56	0.66±0.08
cmp10	7.98±0.05	91.79±0.19	7.28±0.01	97.47±0.70	-0.23±0.04
cmp11	7.74±0.01	102.68±1.33	7.16±0.01	92.88±1.61	-0.27±0.02
cmp12	7.99±0.05	98.38±1.80	7.35±0.01	89.51±0.66	-0.22±0.04
cmp13	5.70±0.03	117.00±2.04	5.31±0.02	94.66±1.15	-0.42±0.02
cmp14	5.73±0.02	94.46±1.10	4.87±0.03	93.33±0.32	-0.03±0.03
cmp15	7.80±0.04	71.78±0.72	7.00±0.01	96.49±1.11	-0.22±0.03
cmp16	7.68±0.04	76.51±1.62	6.41±0.02	97.77±0.92	0.27±0.03
cmp17	N.D	N.D	N.D	N.D	N.D
cmp18	5.69±0.06	56.07±0.48	5.06±0.01	61.60±0.83	-0.32±0.05
cmp19	N.D	N.D	N.D	N.D	N.D
cmp20	6.98±0.01	102.01±0.02	6.16±0.03	92.86±1.78	-0.06±0.05
cmp21	5.96±0.05	96.79±0.20	4.75±0.02	91.15±0.87	0.34±0.05
cmp22	5.07±0.02	96.91±0.49	4.72±0.03	53.62±0.68	-0.29±0.02
cmp23	N.D	N.D	N.D	N.D	N.D
cmp24	6.18±0.01	98.93±0.69	5.31±0.03	79.75±1.04	0.07±0.03
cmp25	N.D	N.D	N.D	N.D	N.D
cmp26	7.64±0.02	98.07±0.78	6.90±0.03	81.95±1.47	-0.08±0.03
cmp27	7.47±0.10	106.07±2.57	6.14±0.14	99.18±2.89	-0.00±0.08
cmp28	7.72±0.10	97.35±1.11	6.21±0.18	99.04±2.98	-0.15±0.09
cmp29	8.78±0.13	84.14±2.58	7.53±0.14	97.78±3.13	-0.17±0.03
cmp30	8.31±0.14	94.43±2.30	6.77±0.13	98.51±2.29	-0.16±0.02
cmp31	N.D	N.D	N.D	N.D	N.D
cmp32	7.80±0.20	91.70±2.32	6.19±0.13	98.39±3.63	-0.22±0.05

Continued

Ligands	G protein signaling		β-arrestin2 recruitment		Bias factor*
	pEC50	Emax (% difelikefalin)	pEC50	Emax (% difelikefalin)	
cmp33	N.D	N.D	N.D	N.D	N.D
cmp34	9.21±0.24	84.31±4.28	7.63±0.25	112.56±6.51	-0.09±0.04
cmp35	8.58±0.17	94.15±4.20	7.44±0.21	97.41±2.81	-0.23±0.08
cmp36	8.74±0.07	95.39±4.90	7.29±0.03	100.80±3.35	0.06±0.04
cmp37	7.29±0.53	88.93±8.01	6.36±0.06	72.81±16.0	0.24±0.08
cmp38	8.65±0.17	90.77±2.65	6.94±0.17	97.12±1.68	0.32±0.07
cmp39	8.87±0.01	94.07±2.63	7.23±0.03	96.14±0.81	0.26±0.04
cmp40	9.13±0.25	100.23±0.30	7.77±0.19	109.73±3.42	-0.04±0.08
cmp41	8.44±0.19	101.70±3.21	6.94±0.20	105.64±1.04	0.11±0.06
cmp42	7.75±0.15	102.88±5.77	5.99±0.16	103.63±2.97	0.39±0.03
cmp43	8.55±0.26	101.76±0.79	6.87±0.22	95.83±1.93	0.35±0.04
cmp44	6.69±0.09	99.96±4.00	5.30±0.02	63.24±3.11	0.34±0.03
cmp45	5.41±0.14	90.68±9.24	4.81±0.16	35.80±4.43	-0.21±0.03
cmp46	8.32±0.10	97.04±2.89	6.91±0.17	95.02±1.48	0.07±0.08
cmp47	8.50±0.24	91.91±2.39	6.51±0.27	91.34±2.50	0.26±0.05
cmp48	7.43±0.21	101.45±3.81	5.76±0.23	92.89±2.08	-0.02±0.04
cmp49	8.98±0.15	97.41±0.98	7.36±0.14	106.44±0.80	0.22±0.04
cmp50	9.00±0.02	98.20±8.02	7.83±0.03	103.92±1.30	-0.21±0.01
cmp51	9.16±0.04	86.56±4.41	7.81±0.01	105.58±2.86	-0.10±0.05
cmp52	5.57±0.10	107.97±9.97	5.06±0.10	92.14±3.53	-0.78±0.05
cmp53	8.49±0.06	88.19±7.78	7.11±0.05	101.02±2.31	-0.04±0.04
cmp54	8.34±0.08	95.32±4.20	6.70±0.07	103.79±0.50	0.24±0.05
cmp55	8.74±0.07	112.20±4.78	7.36±0.05	86.94±4.02	0.13±0.08
cmp56	8.33±0.10	97.64±8.66	7.23±0.05	112.08±3.77	-0.32±0.04
cmp57	8.46±0.13	91.98±5.88	7.28±0.19	104.18±2.47	-0.25±0.06
cmp58	8.44±0.04	101.94±4.73	7.33±0.09	101.30±0.67	-0.25±0.04

N.D, not detectable or cannot be established over the tested concentration rang.

* bias factor >0 means Gi-biased; <0 means β-arrestin-biased.

Supplementary Table 5 | List of primers sequences for site-direct mutagenesis studies.

Residue	Primers	
KOR-V108$^{2.53}$A	Forward	CTGATGCCCTGGCTACAACAACCATG
	Reverse	CATGGTTGTTGTAGCCAGGGCATCAG
KOR-T111$^{2.56}$A	Forward	CCTGGTTACAACAGCCATGCCTTTCCAGAG
	Reverse	CTCTGGAAAGGCATGGCTGTTGTAACCAGG
KOR-Q115$^{2.60}$A	Forward	CCATGCCTTTCGCGAGCACAGTGTAC
	Reverse	CCATGCCTTTCGCGAGCACAGTGTAC
KOR-V134$^{3.28}$A	Forward	GCTAATCACGATCTTGGCCAGCACATCGCCG
	Reverse	GTCAATGCTAATCGCGATCTTGCACAGC
KOR-I135$^{3.29}$A	Forward	GTGCAAGATCGTGGCTAGCATTGACTAC
	Reverse	GTAGTCAATGCTAGCCACGATCTTGCAC
KOR-D138$^{3.32}$A	Forward	GTGATTAGCATTGCCTACTACAACATG
	Reverse	CATGTTGTAGTAGCCAATGCTAATCAC
KOR-Y139$^{3.33}$A	Forward	GATTAGCATTGACGCCTACAACATGTTC
	Reverse	GAACATGTTGTAGGCGTCAATGCTAATC
KOR-M142$^{3.36}$A	Forward	GACTACTACAACGCGTTCACCAGCATC
	Reverse	GATGCTGGTGAACGCGTTGTAGTAGTC
KOR-E209^{ECL2}A	Forward	GTGGACGTGATCGCGTGCAGCCTGCAG
	Reverse	CTGCAGGCTGCACGCGATCACGTCCAC
KOR-S211^{ECL2}A	Forward	GTGATCGAGTGCGCCCTGCAGTTCCCAG
	Reverse	GATCGAGTGCAGCGCGCAGTTCCCAGACGAC
KOR-D223$^{5.35}$A	Forward	GATTACAGCTGGTGGGCCCTGTTCATGAAAATATG
	Reverse	CATATTTTCATGAACAGGGCCCACCAGCTGTAATC
KOR-K227$^{5.39}$A	Forward	GGACCTGTTCATGGCAATATGTGTGTTCATC
	Reverse	GATGAACACACATATTGCCATGAACAGGTCC
KOR-V230$^{5.42}$A	Forward	CATGAAAATATGTGCGTTCATCTTTGCC
	Reverse	GGCAAAGATGAACGCACATATTTTCATG
KOR-W287$^{6.48}$A	Forward	GTTCGTGGTGTGCGCGACCCCTATTCATATC
	Reverse	GATATGAATAGGGGTCGCGCACACCACGAAC
KOR-I294$^{6.55}$A	Forward	CTATTCATATCTTCGCCCTCGTGGAAGCCCTG
	Reverse	CTATTCATATCTTCGCCCTCGTGGAAGCCCTG
KOR-I316$^{7.39}$A	Forward	CTTATTACTTCTGCGCCGCCCTGGGCTACACC
	Reverse	GGTGTAGCCCAGGGCGGCGCAGAAGTAATAAG
KOR-Y320$^{7.43}$A	Forward	CATCGCCCTGGGCGCCACCAACTCATCTC
	Reverse	GTTCAGAGATGAGTTGGTGGCGCCCAGGGCGATGCAG
KOR$^{\triangle M}$-L333M	Forward	CTATCCTGTACGCCTTCATGGACGAGAACTTCAAGC
	Reverse	GCTTGAAGTTCTCGTCCATGAAGGCGTACAGGATAG
MOR-Y328$^{7.43}$A	Forward	GTATCGCTCTGGGCGCCACCAATAGCTGCC
	Reverse	GGCAGCTATTGGTGGCGCCCAGAGCGATAC
DOR-Y308$^{7.43}$A	Forward	CATCGCCCTGGGCGCCGCCAACAGCAGCCTG
	Reverse	CAGGCTGCTGTTGGCGGCGCCCAGGGCGATG

a

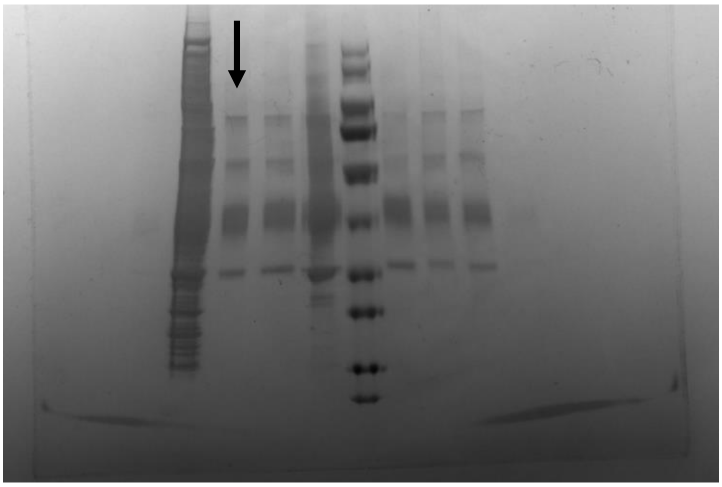

b

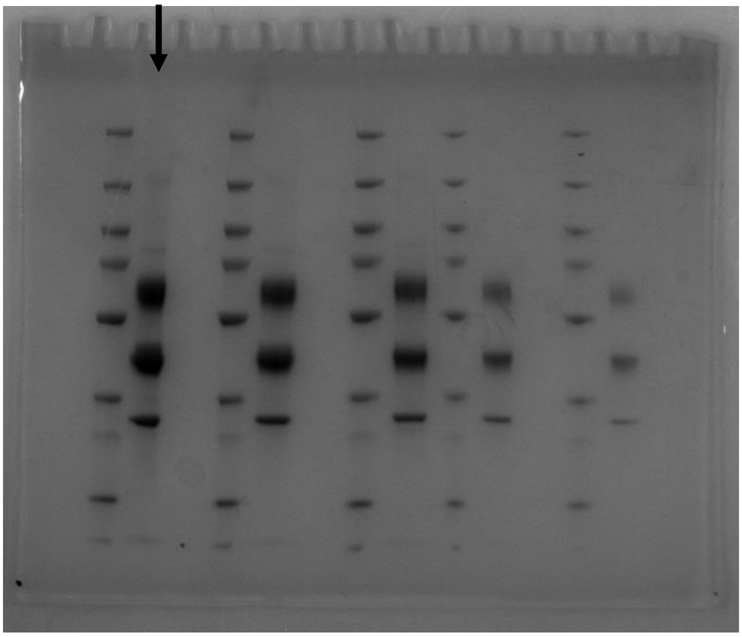

Supplementary information. Uncropped gels.

Uncropped gels for the KOR-Gi bound to (a) difelikefalin and (b) beta01.
Arrows show the lines used in the Supplementary Fig. 1a and Supplementary Fig. 8a.

Supplementary Notes Certificates of Compound Analysis

KS-V PEPTIDE
The Peptide Experts

Certificate of Analysis

Product Information

Customer Code:	beta01	KSVP Code:	KP3578
Lot No.:	20240702	Formula:	
M. W.:	679.93	Quantity:	50.0mg

Sequence:

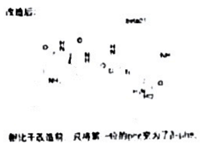

Store at: -20℃

Analytical Results

Tests	Specifications	Results
Appearance	Report Result	white powder
Purity(HPLC)	≥95%	99.88%
MS(ESI)	Calc.679.93	Obs.679.50

Prepared By: 吴玲玲 Checked by: 纪焕 Date: 2024.07.08

KS-V PEPTIDE
The Peptide Experts

HPLC Report

Sample Information

Customer Code:	beta01	KSVP Code:	KP3578
Lot No.:	20240702		
Phase A:	0.1% TFA in deionized water		
Phase B:	0.08% TFA in acetonitrile		
Flow rate:	1.0mL/min		
Wavelength:	214nm		
Analytical column type:	YMC-Pack ODS-A、120A、5um、4.6*250mm		
Dissolution method:	100%H2O		
Inj. Volume:	4uL		
Date Acquired:	20240705,20:24:32		

	Time（min）	Phase A	Phase B
Gradient:	0.01	95%	5%
	2.00	95%	5%
	22.00	1%	99%
	30.00	Stop	

Chromatogram

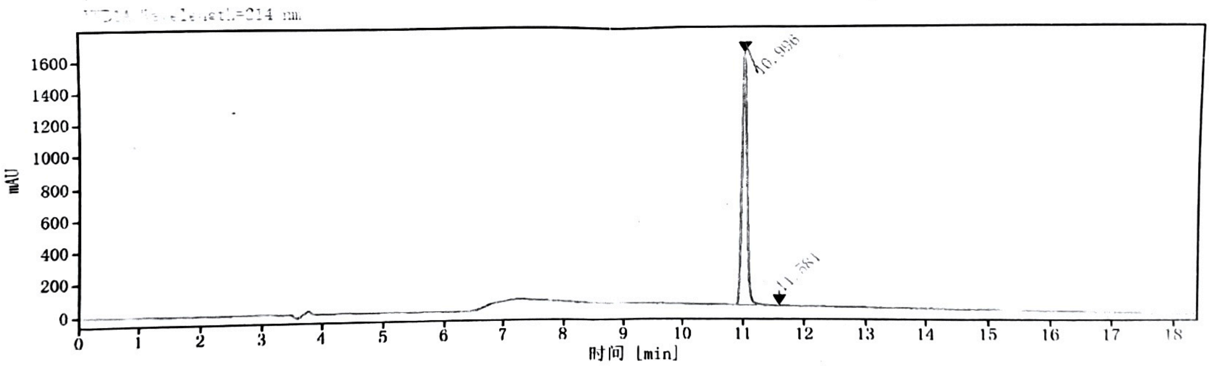

Peak	Ret time	Area	Area%	Height	Height%
1	10.996	9413.7902	99.88	1601.631	99.94
2	11.581	11.5339	0.12	0.946	0.06

Mass Spectrometry Report

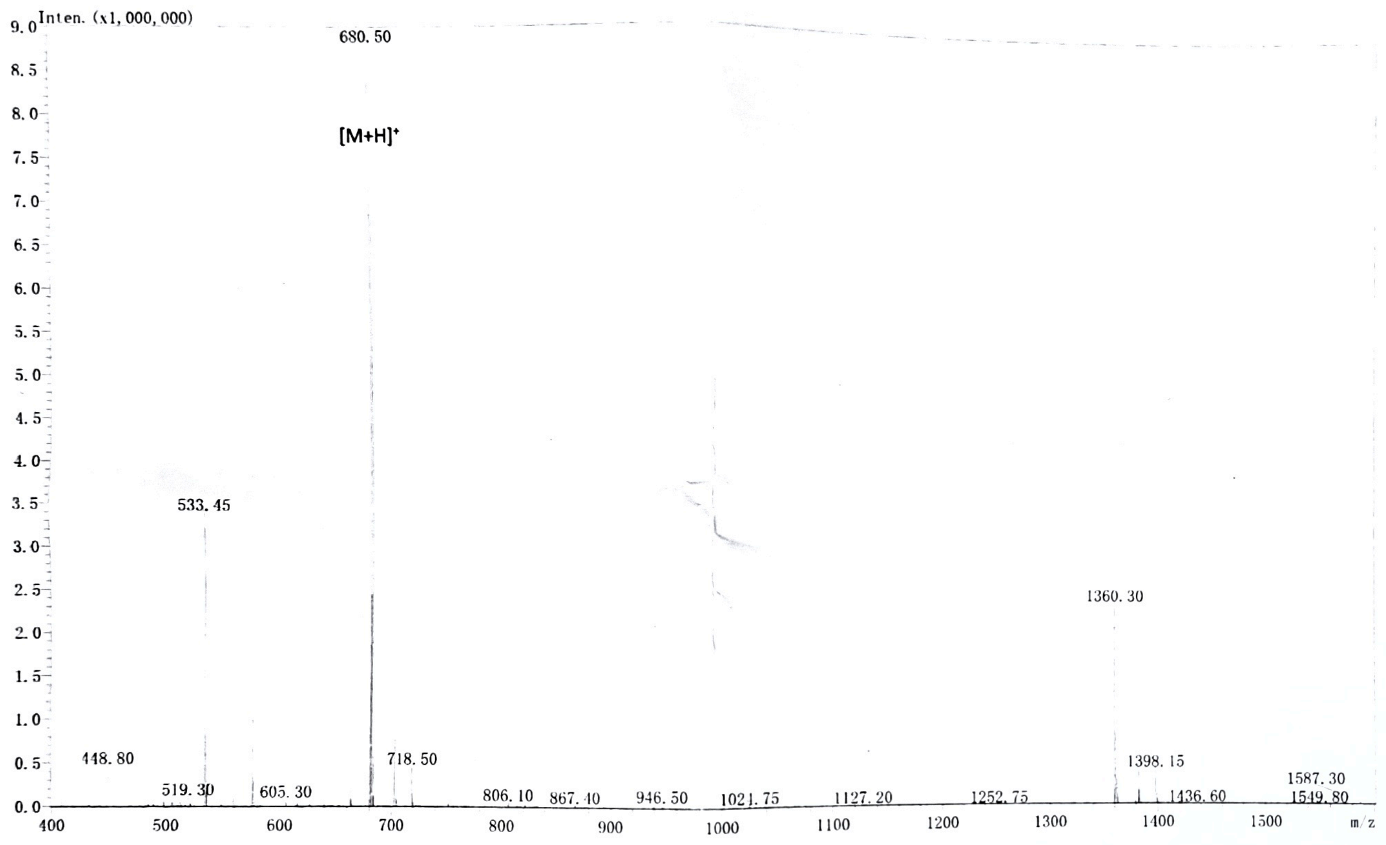

Sample Description		Instrument	SHIMADZU LCMS-2020		
Analyzed date:	20240705,19:22	Probe:	ESI	Probe Bias:	+4.5kv
Analyst:	WCG	Nebulizer Gas Flow:	1.5L/min	Detector:	1.5kv
Sample:	beta01;KP3578	CDL:	-20.0v	T. Flow:	0.2ml/min
M.W.:	679.93	CDL Temp.:	250 °C	B. Conc.:	30%H2O/30%ACN/40%MEOH
Lot. No.:	20240702	Block Temp.:	200 °C		

KS-V PEPTIDE
The Peptide Experts

Certificate of Analysis

Product Information

Customer Code:	cmp1	KSVP Code:	KP3610-1
Lot No.:	20240723	Formula:	
M. W.:	603.17	Quantity:	5.0mg

Sequence:

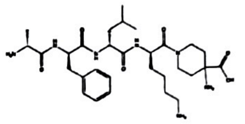

cmp1

Store at: -20℃

Analytical Results

Tests	Specifications	Results
Appearance	Report Result	white powder
Purity(HPLC)	≥95%	99.89%
MS(ESI)	Calc.603.17	Obs.603.65

Prepared By: 吴玲玲　　Checked by: [illegible]　　Date: 2024.07.25

KS-V PEPTIDE
The Peptide Experts

HPLC Report

Sample Information

Customer Code:	cmp1	KSVP Code:	KP3610-1
Lot No.:	20240723		
Phase A:	0.1% TFA in deionized water		
Phase B:	0.08% TFA in acetonitrile		
Flow rate:	1.0mL/min		
Wavelength:	214nm		
Analytical column type:	YMC-Pack ODS-A、120A、5um、4.6*250mm		
Dissolution method:	100%H_2O		
Inj. Volume:	10uL		
Date Acquired:	20240725,12:14:25		

	Time（min）	Phase A	Phase B
	0.01	95%	5%
Gradient:	2.00	95%	5%
	22.00	1%	99%
	30.00	Stop	

Chromatogram

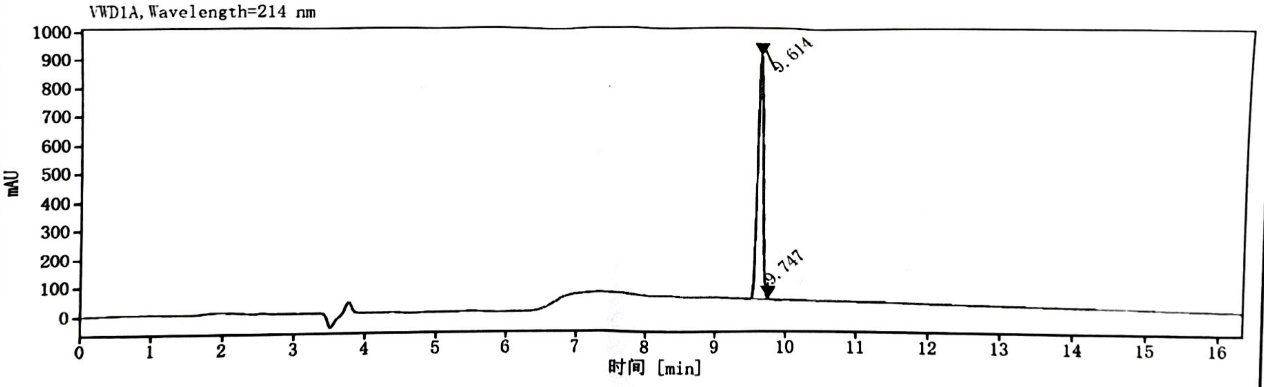

Peak	Ret time	Area	Area%	Height	Height%
1	9.614	4544.6326	99.89	866.434	99.51
2	9.747	5.0513	0.11	4.295	0.49

KS-V PEPTIDE
The Peptide Experts

Mass Spectrometry Report

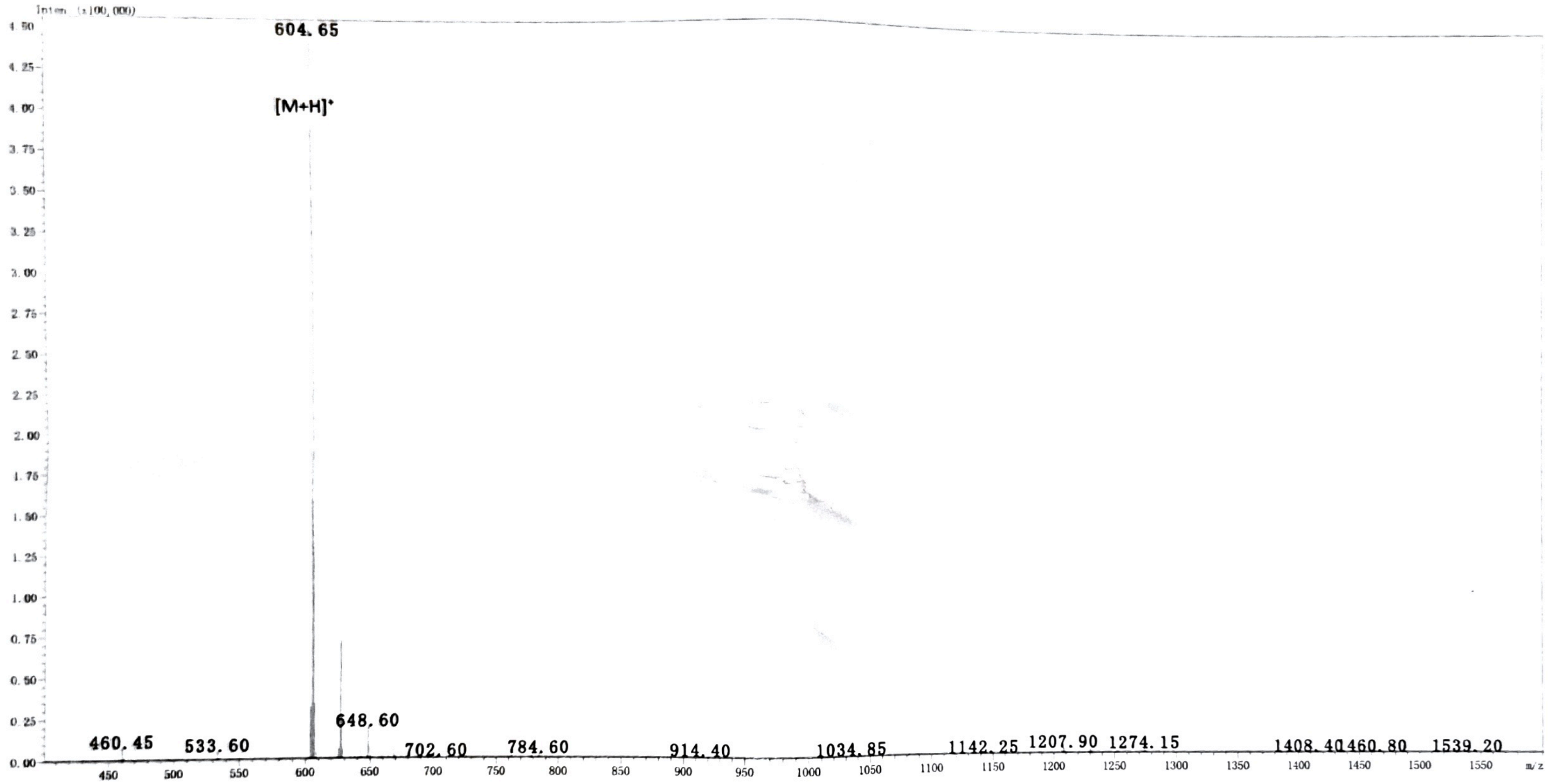

Sample Description

Analyzed date: 20240725,14:59

Analyst: WCG

Sample: cmp1;KP3610-1

M.W.: 603.17

Lot. No.: 20240723

Instrument SHIMADZU LCMS-2020

Probe: ESI

Nebulizer Gas Flow: 1.5L/min

CDL: -20.0v

CDL Temp.: 250°C

Block Temp.: 200°C

Probe Bias: +4.5kv

Detector: 1.5kv

T. Flow: 0.2ml/min

B. Conc.: 0.05%FA in 30%H_2O/30%ACN/40%MeOH

KS-V PEPTIDE
The Peptide Experts

Certificate of Analysis

Product Information

Customer Code:	cmp2	KSVP Code:	KP3610-2
Lot No.:	20240723	Formula:	
M. W.:	631.89	Quantity:	5.0mg

Sequence:

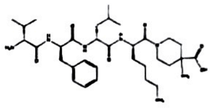

cmp2

Store at: -20℃

Analytical Results

Tests	Specifications	Results
Appearance	Report Result	white powder
Purity(HPLC)	≥95%	99.82%
MS(ESI)	Calc.631.89	Obs.631.65

Prepared By: 吴玲玲 Checked by: 纪焕 Date: 2024.07.25

KS-V PEPTIDE
The Peptide Experts

HPLC Report

Sample Information

Customer Code:	Cmp2	KSVP Code:	KP3610-2
Lot No.:	20240723		
Phase A:	0.1% TFA in deionized water		
Phase B:	0.08% TFA in acetonitrile		
Flow rate:	1.0mL/min		
Wavelength:	214nm		
Analytical column type:	YMC-Pack ODS-A、120A、5um、4.6*250mm		
Dissolution method:	100%H_2O		
Inj. Volume:	10uL		
Date Acquired:	20240725,12:58:35		

	Time（min）	Phase A	Phase B
Gradient:	0.01	95%	5%
	2.00	95%	5%
	22.00	1%	99%
	30.00	Stop	

Chromatogram

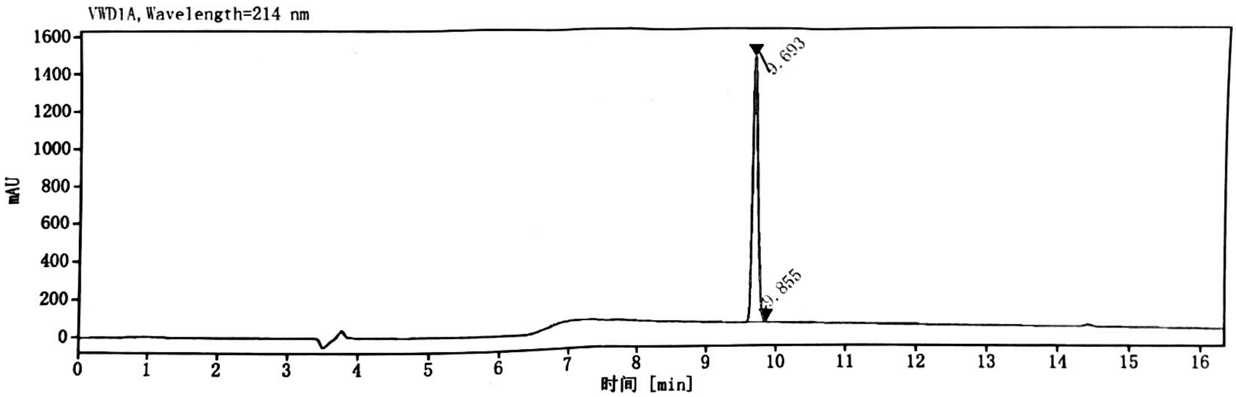

Peak	Ret time	Area	Area%	Height	Height%
1	9.693	7794.4487	99.82	1435.126	99.76
2	9.855	13.8693	0.18	3.497	0.24

KS-V PEPTIDE
The Peptide Experts

Mass Spectrometry Report

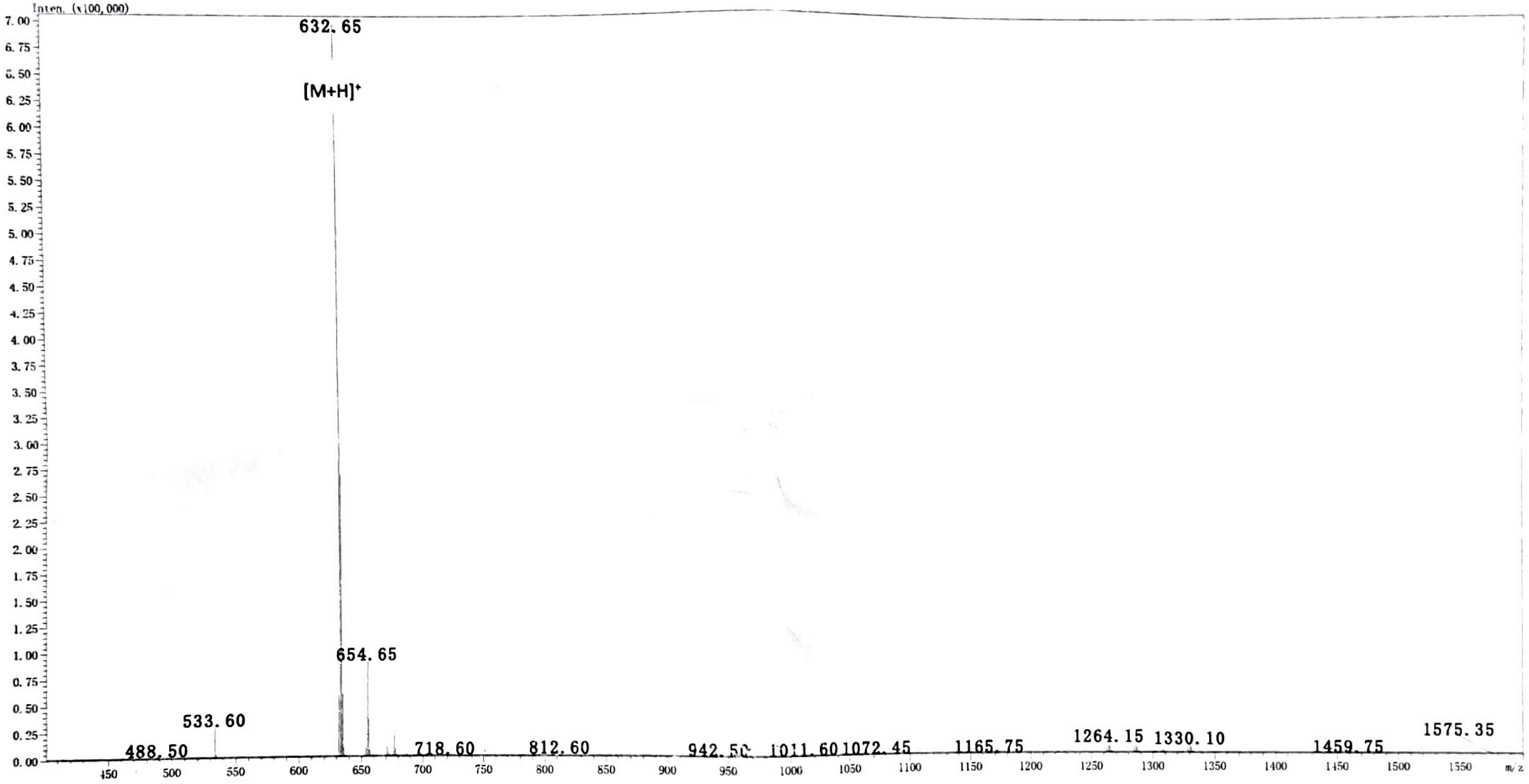

Sample Description		Instrument	SHIMADZU LCMS-2020		
Analyzed date:	20240725,15:09	Probe:	ESI	Probe Bias:	+4.5kv
Analyst:	WCG	Nebulizer Gas Flow:	1.5L/min	Detector:	1.5kv
Sample:	cmp2;KP3610-2	CDL:	-20.0v	T. Flow:	0.2ml/min
M.W.:	631.89	CDL Temp.:	250°C	B. Conc.:	0.05%FA in 30%H_2O/30%ACN/40%MeOH
Lot. No.:	20240723	Block Temp.:	200°C		

KS-V PEPTIDE
The Peptide Experts

Certificate of Analysis

Product Information

Customer Code:	cmp3	KSVP Code:	KP3610-3
Lot No.:	20240723	Formula:	
M. W.:	645.92	Quantity:	5.0mg

Sequence:

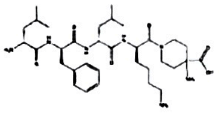

cmp3

Store at: -20℃

Analytical Results

Tests	Specifications	Results
Appearance	Report Result	white powder
Purity(HPLC)	≥95%	99.95%
MS(ESI)	Calc.645.92	Obs.645.65

Prepared By: 吴玲玲

Checked by: 纪 [illegible]

Date: 2024.07.29

KS-V PEPTIDE
The Peptide Experts

HPLC Report

Sample Information

Customer Code:	cmp3	KSVP Code:	KP3610-3
Lot No.:	20240723		
Phase A:	0.1% TFA in deionized water		
Phase B:	0.08% TFA in acetonitrile		
Flow rate:	1.0mL/min		
Wavelength:	214nm		
Analytical column type:	YMC-Pack ODS-A、120A、5um、4.6*250mm		
Dissolution method:	100%H_2O		
Inj. Volume:	10uL		
Date Acquired:	20240726,11:35:43		

	Time（min）	Phase A	Phase B
Gradient:	0.01	95%	5%
	2.00	95%	5%
	22.00	1%	99%
	30.00	Stop	

Chromatogram

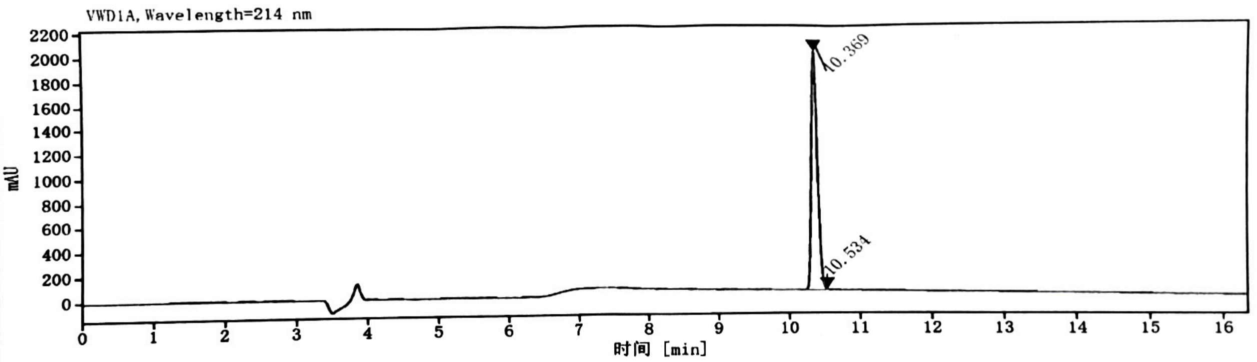

Peak	Ret time	Area	Area%	Height	Height%
1	10.369	12036.2168	99.95	1985.829	99.76
2	10.534	6.0536	0.05	4.747	0.24

Mass Spectrometry Report

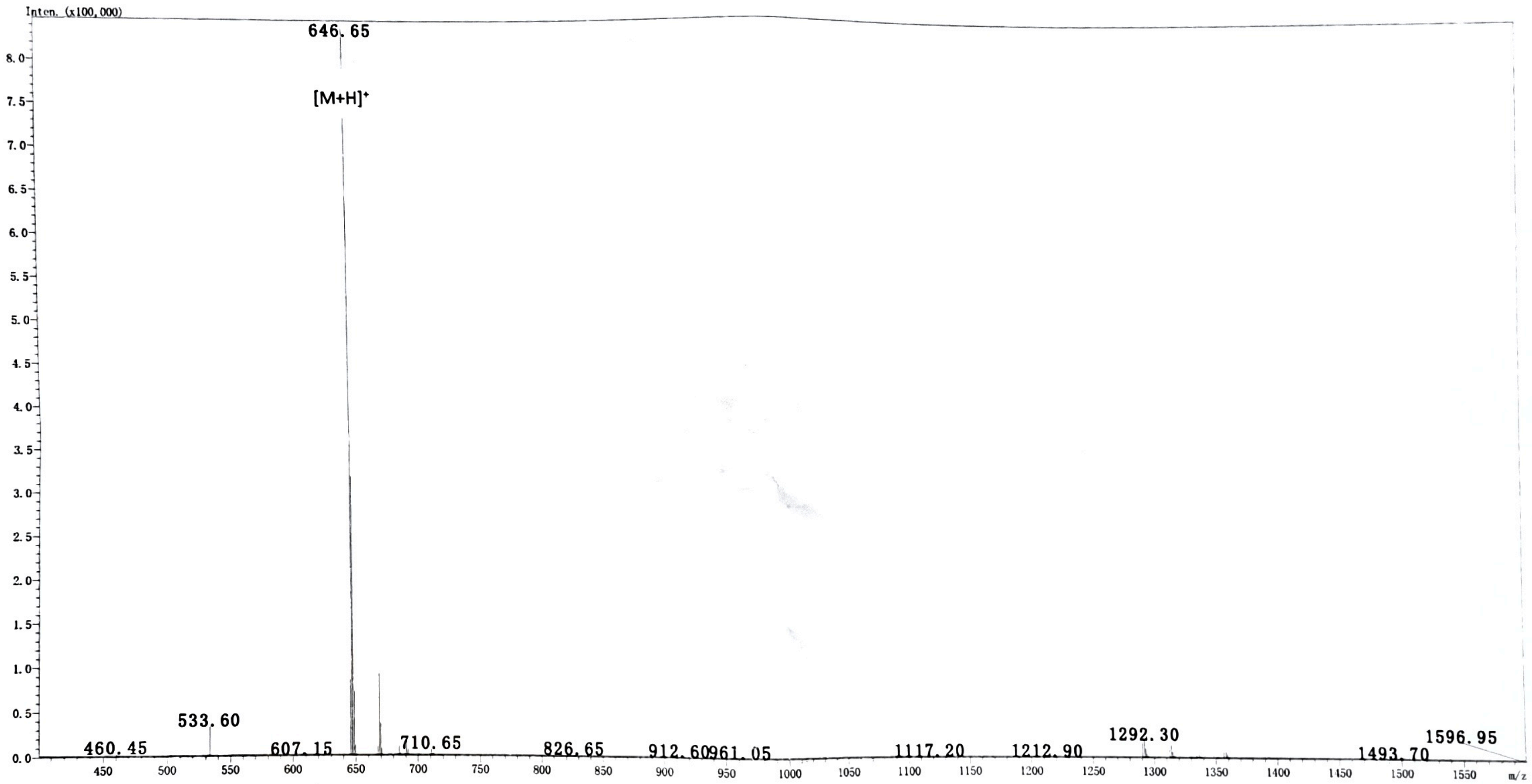

Sample Description

Analyzed date: 20240725,15:16

Analyst: WCG

Sample: cmp3;KP3610-3

M.W.: 645.92

Lot. No.: 20240723

Instrument SHIMADZU LCMS-2020

Probe: ESI

Nebulizer Gas Flow: 1.5L/min

CDL: -20.0v

CDL Temp.: 250℃

Block Temp.: 200℃

Probe Bias: +4.5kv

Detector: 1.5kv

T. Flow: 0.2ml/min

B. Conc.: 0.05%FA in 30%H_2O/30%ACN/40%MeOH

KS-V PEPTIDE
The Peptide Experts

Certificate of Analysis

Product Information

Customer Code: cmp4

KSVP Code: KP3610-4

Lot No.: 20240723

Formula:

M. W.: 619.84

Quantity: 5.0mg

Sequence:

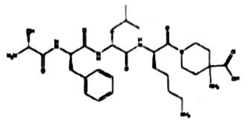

cmp4

Store at: -20℃

Analytical Results

Tests	Specifications	Results
Appearance	Report Result	white powder
Purity(HPLC)	≥95%	99.53%
MS(ESI)	Calc.619.84	Obs.619.35

质检专用章

Prepared By: 吴玲玲

Checked by: 纪娥

Date: 2024.07.29

KS-V PEPTIDE
The Peptide Experts

HPLC Report

Sample Information

Customer Code:	cmp4	KSVP Code:	KP3610-4
Lot No.:	20240723		
Phase A:	0.1% TFA in deionized water		
Phase B:	0.08% TFA in acetonitrile		
Flow rate:	1.0mL/min		
Wavelength:	214nm		
Analytical column type:	YMC-Pack ODS-A、120A、5um、4.6*250mm		
Dissolution method:	100%H_2O		
Inj. Volume:	10uL		
Date Acquired:	20240726,12:24:16		

	Time（min）	Phase A	Phase B
Gradient:	0.01	95%	5%
	2.00	95%	5%
	22.00	1%	99%
	30.00	Stop	

Chromatogram

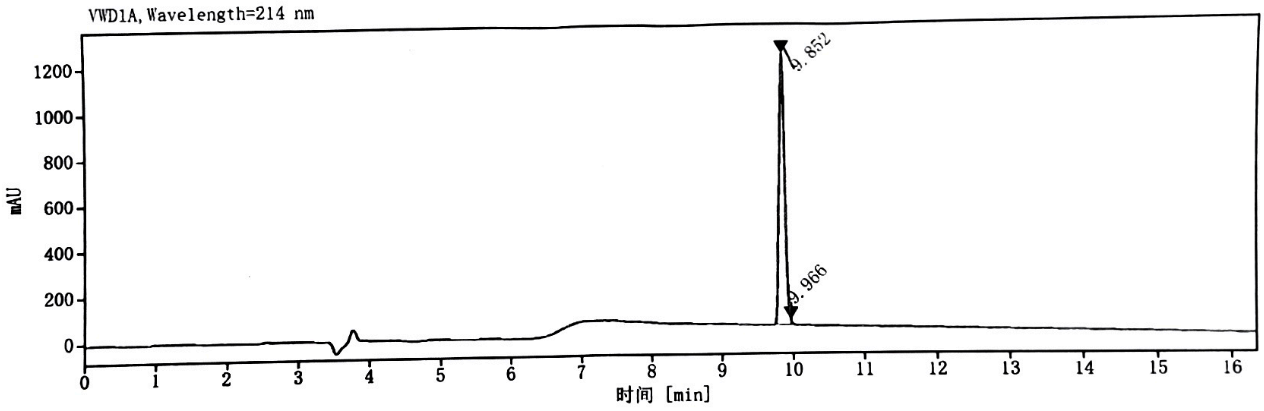

Peak	Ret time	Area	Area%	Height	Height%
1	9.852	6512.5052	99.53	1194.142	98.15
2	9.966	30.6292	0.47	22.521	1.85

Mass Spectrometry Report

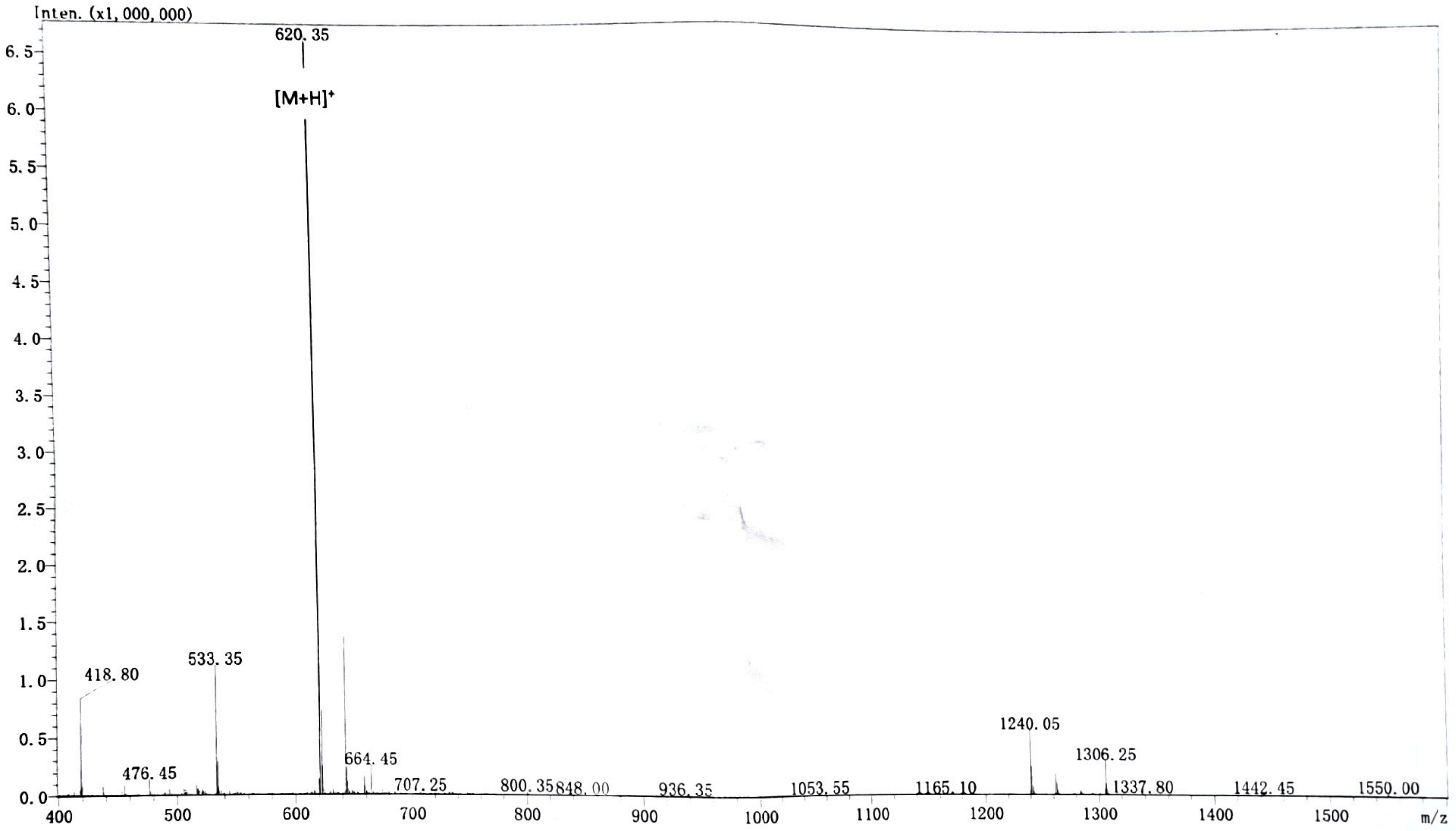

Sample Description

Analyzed date: 20240726,14:16

Analyst: WCG

Sample: cmp4;KP3610-4

M.W.: 619.84

Lot. No.: 20240723

Instrument SHIMADZU LCMS-2020

Probe: ESI

Nebulizer Gas Flow: 1.5L/min

CDL: -20.0v

CDL Temp.: 250℃

Block Temp.: 200℃

Probe Bias: +4.5kv

Detector: 1.5kv

T. Flow: 0.2ml/min

B. Conc.: 0.05%FA in 30%H_2O/30%ACN/40%MeOH

KS-V PEPTIDE
The Peptide Experts

Certificate of Analysis

Product Information

Customer Code:	cmp5	KSVP Code:	KP3610-5
Lot No.:	20240723	Formula:	
M. W.:	678.88	Quantity:	5.0mg
Sequence:	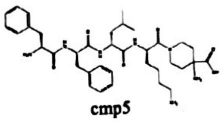		
Store at:	-20℃		

Analytical Results

Tests	Specifications	Results
Appearance	Report Result	white powder
Purity(HPLC)	≥95%	99.89%
MS(ESI)	Calc.678.88	Obs.679.45

Prepared By: 吴玲玲 Checked by: [illegible] Date: 2024.07.29

KS-V PEPTIDE
The Peptide Experts

HPLC Report

Sample Information

Customer Code:	cmp5	KSVP Code:	KP3610-5
Lot No.:	20240723		
Phase A:	0.1% TFA in deionized water		
Phase B:	0.08% TFA in acetonitrile		
Flow rate:	1.0mL/min		
Wavelength:	214nm		
Analytical column type:	YMC-Pack ODS-A、120A、5um、4.6*250mm		
Dissolution method:	100%H_2O		
Inj. Volume:	10uL		
Date Acquired:	20240726,12:59:16		

	Time（min）	Phase A	Phase B
Gradient:	0.01	95%	5%
	2.00	95%	5%
	22.00	1%	99%
	30.00	Stop	

Chromatogram

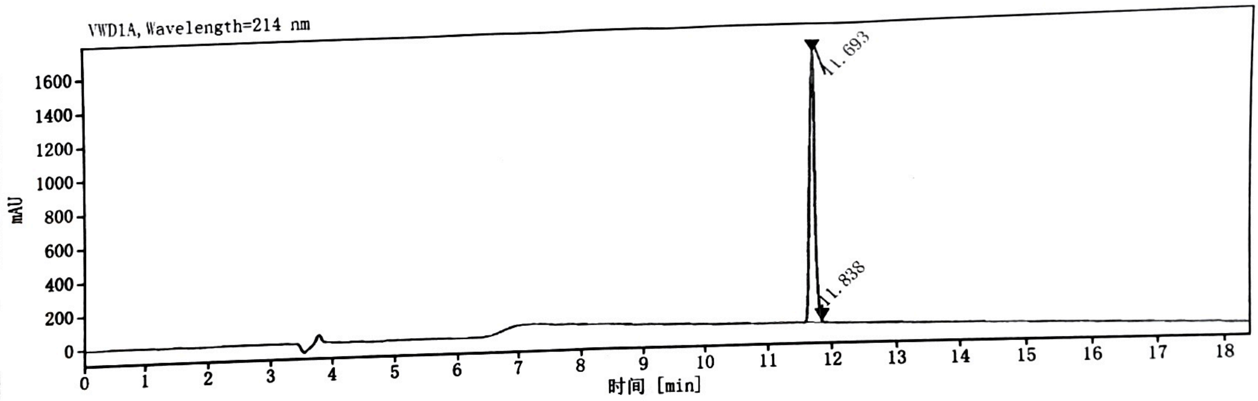

Peak	Ret time	Area	Area%	Height	Height%
1	11.693	9200.2321	99.89	1591.093	99.49
2	11.838	10.1537	0.11	8.109	0.51

KS-V PEPTIDE
The Peptide Experts

Mass Spectrometry Report

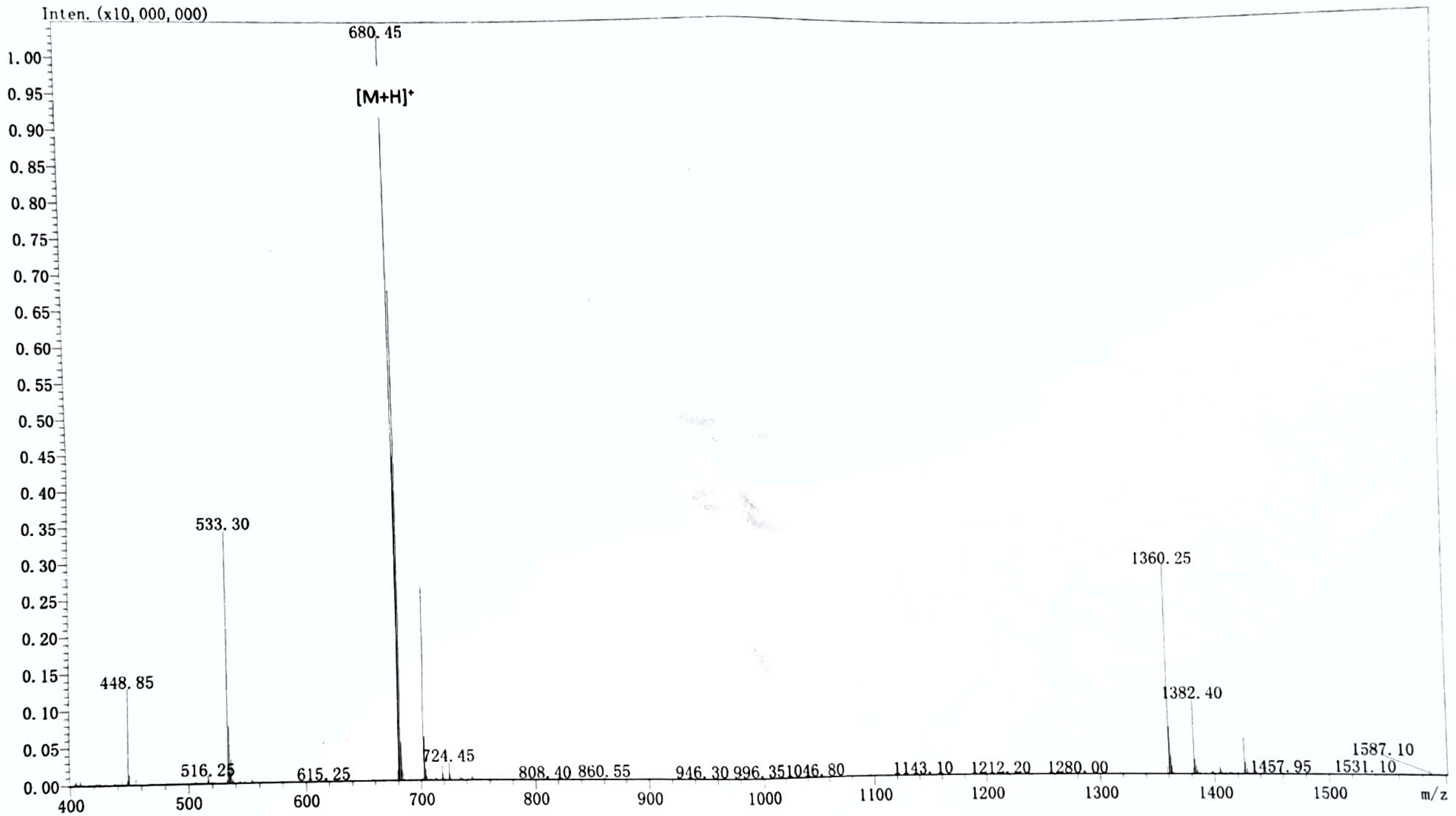

Sample Description

Analyzed date: 20240725,14:35

Analyst: WCG

Sample: cmp5;KP3610-5

M.W. : 678.88

Lot. No. : 20240723

Instrument SHIMADZU LCMS-2020

Probe: ESI

Nebulizer Gas Flow: 1.5L/min

CDL: -20.0v

CDL Temp. : 250°C

Block Temp. : 200°C

Probe Bias: +4.5kv

Detector: 1.5kv

T. Flow: 0.2ml/min

B. Conc. : 0.05%FA in 30%H_2O/30%ACN/40%MeOH

KS-V PEPTIDE
The Peptide Experts

Certificate of Analysis

Product Information

Customer Code:	cmp6	KSVP Code:	KP3610-6
Lot No.:	20240720	Formula:	
M. W.:	729.92	Quantity:	5.0mg

Sequence:

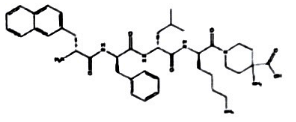

cmp6

Store at: -20℃

Analytical Results

Tests	Specifications	Results
Appearance	Report Result	white powder
Purity(HPLC)	≥95%	99.88%
MS(ESI)	Calc.729.92	Obs.729.40

质检专用章

Prepared By: 吴玲玲 Checked by: [illegible] Date: 2024. 07.23

HPLC Report

Sample Information

Customer Code:	cmp6	KSVP Code:	KP3610-6
Lot No.:	20240720		
Phase A:	0.1% TFA in deionized water		
Phase B:	0.08% TFA in acetonitrile		
Flow rate:	1.0mL/min		
Wavelength:	214nm		
Analytical column type:	YMC-Pack ODS-A、120A、5um、4.6*250mm		
Dissolution method:	100%H_2O		
Inj. Volume:	10uL		
Date Acquired:	20240723,10:16:00		

Gradient:	Time（min）	Phase A	Phase B
	0.01	95%	5%
	2.00	95%	5%
	22.00	1%	99%
	30.00	Stop	

Chromatogram

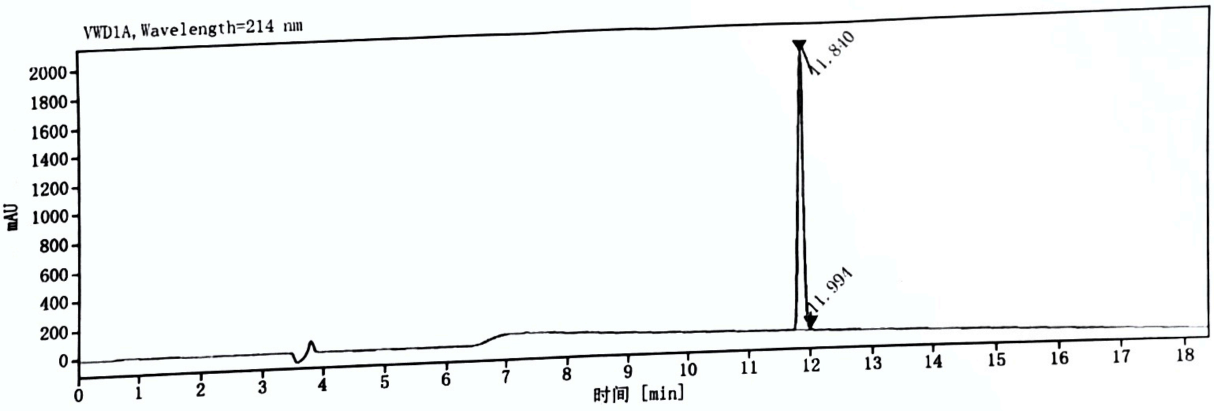

Peak	Ret time	Area	Area%	Height	Height%
1	11.840	11174.087	99.88	1934.411	99.65
2	11.994	13.0848	0.12	6.717	0.35

Mass Spectrometry Report

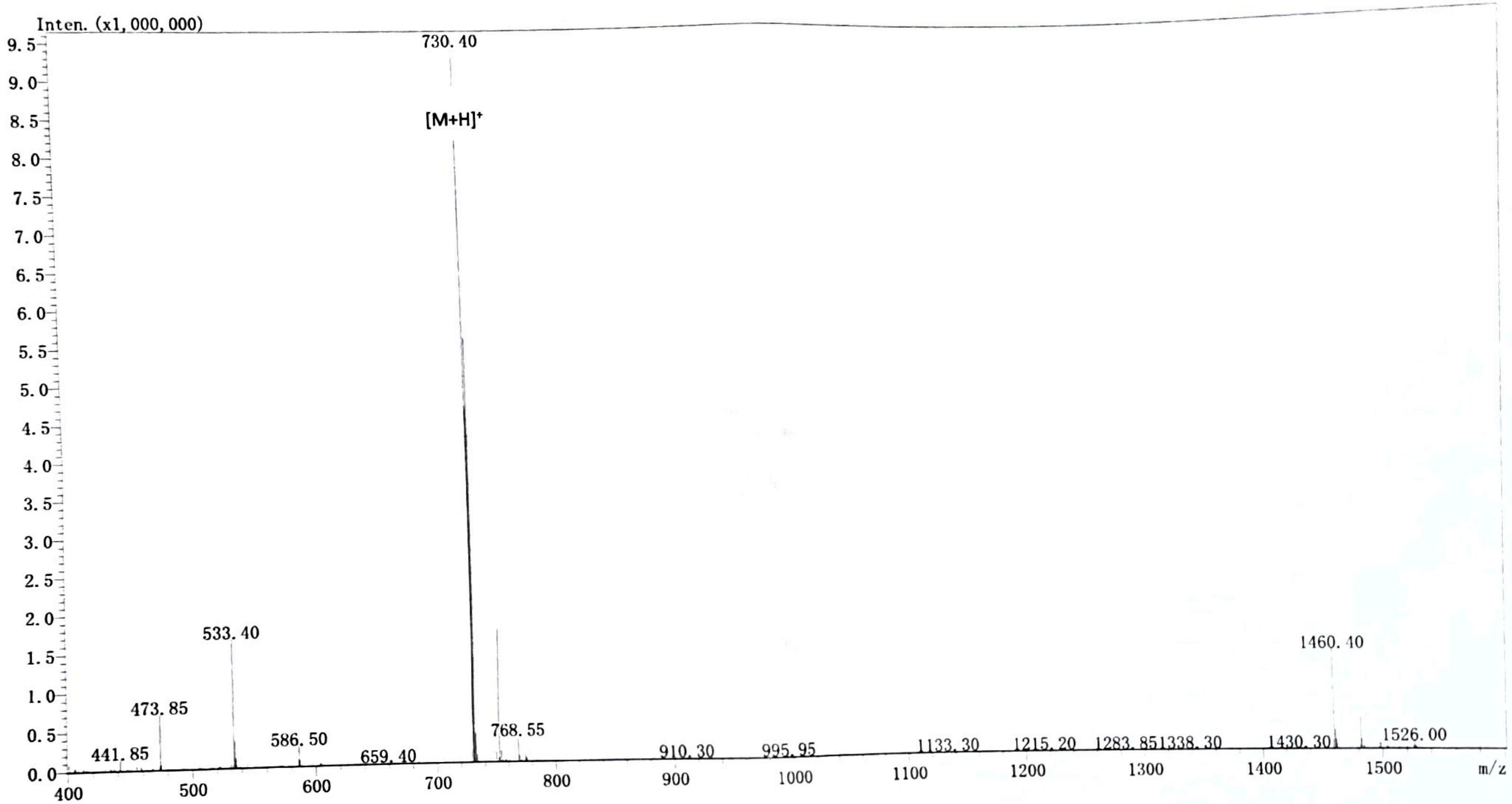

Sample Description

Analyzed date: 20240722,17:13

Analyst: WCG

Sample: cmp6;KP3610-6

M.W.: 729.92

Lot. No.: 20240720

Instrument SHIMADZU LCMS-2020

Probe: ESI

Nebulizer Gas Flow: 1.5L/min

CDL: -20.0v

CDL Temp.: 250°C

Block Temp.: 200°C

Probe Bias: +4.5kv

Detector: 1.5kv

T. Flow: 0.2ml/min

B. Conc.: 0.05%FA in 30%H_2O/30%ACN/40%MeOH

KS-V PEPTIDE
The Peptide Experts

Certificate of Analysis

Product Information

Customer Code: cmp7

Lot No.: 20240723

M. W.: 669.90

Sequence:

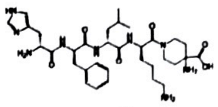

cmp7

Store at: -20°C

KSVP Code: KP3610-7

Formula:

Quantity: 5.0mg

Analytical Results

Tests	Specifications	Results
Appearance	Report Result	white powder
Purity(HPLC)	≥95%	99.79%
MS(ESI)	Calc.669.90	Obs.669.55

质检专用章

Prepared By: 吴玲玲

Checked by: 纪 焕

Date: 2024.07.29

KS-V PEPTIDE
The Peptide Experts

HPLC Report

Sample Information

Customer Code: cmp7

KSVP Code: KP3610-7

Lot No.: 20240723

Phase A: 0.1% TFA in deionized water

Phase B: 0.08% TFA in acetonitrile

Flow rate: 1.0mL/min

Wavelength: 214nm

Analytical column type: YMC-Pack ODS-A、120A、5um、4.6*250mm

Dissolution method: 100%H_2O

Inj. Volume: 10uL

Date Acquired: 20240726,13:39:02

	Time（min）	Phase A	Phase B
Gradient:	0.01	95%	5%
	2.00	95%	5%
	22.00	1%	99%
	30.00	Stop	

Chromatogram

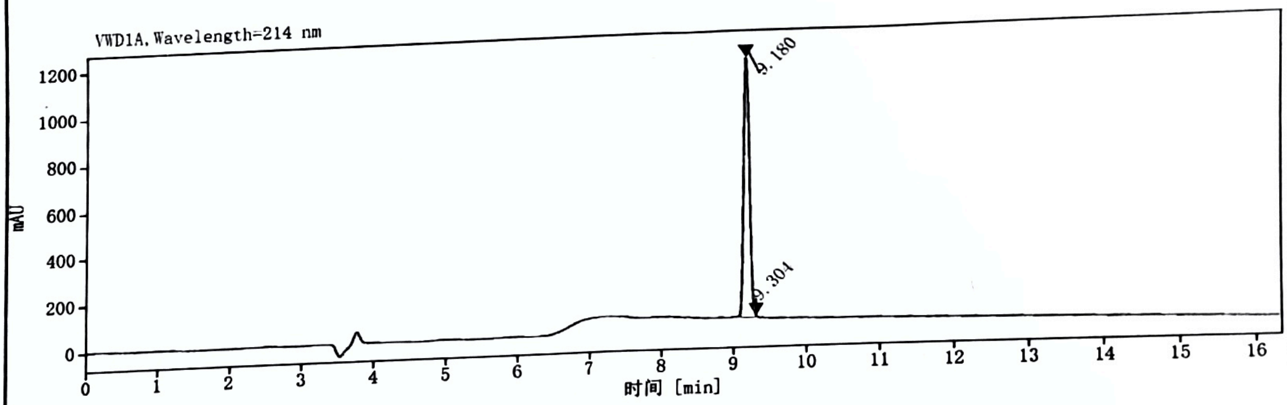

Peak	Ret time	Area	Area%	Height	Height%
1	9.180	5979.0304	99.79	1111.128	99.08
2	9.304	12.5725	0.21	10.273	0.92

Mass Spectrometry Report

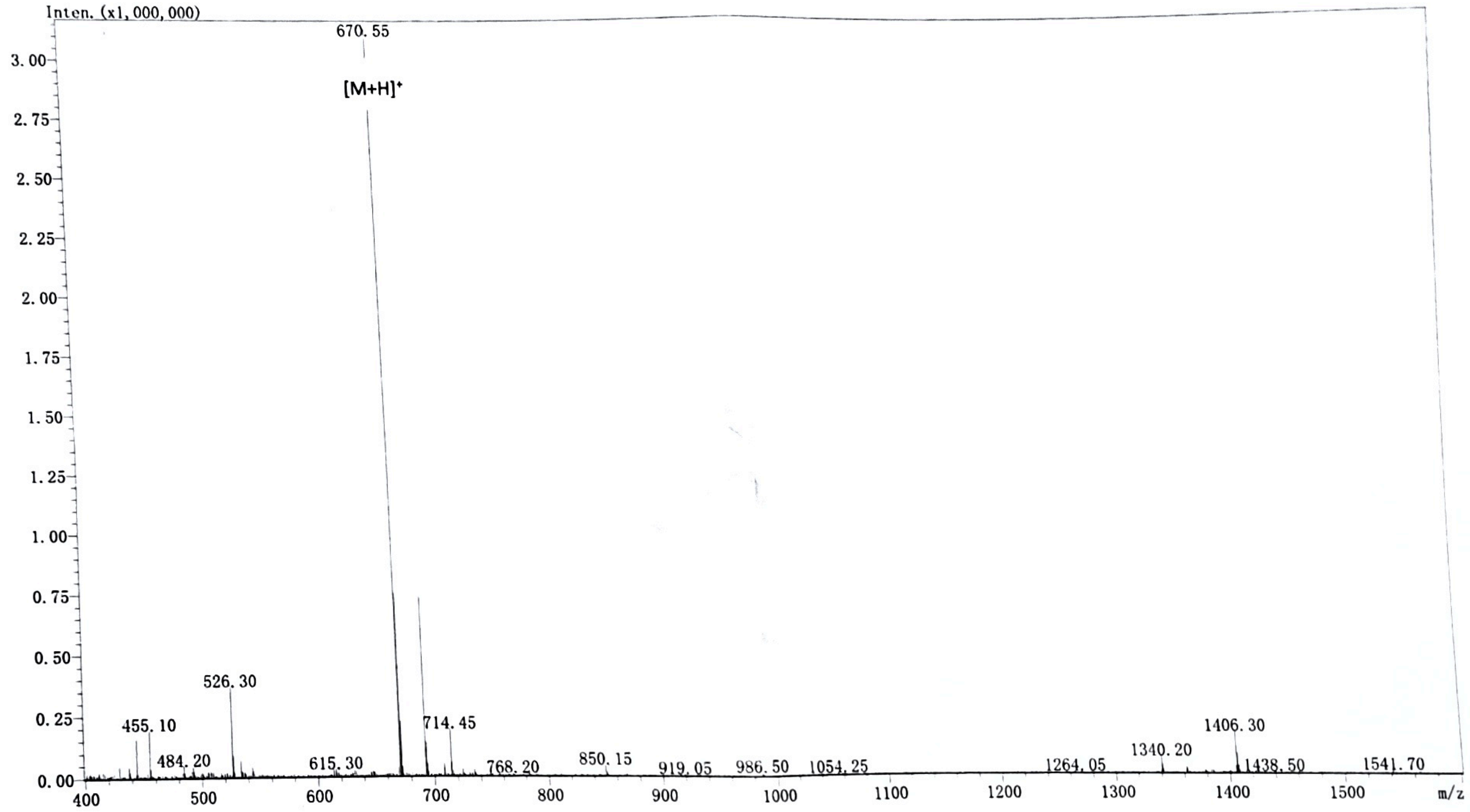

Sample Description

Analyzed date: 20240725,14:37

Analyst: WCG

Sample: cmp7;KP3610-7

M.W.: 669.90

Lot. No.: 20240723

Instrument SHIMADZU LCMS-2020

Probe: ESI

Nebulizer Gas Flow: 1.5L/min

CDL: -20.0v

CDL Temp.: 250℃

Block Temp.: 200℃

Probe Bias: +4.5kv

Detector: 1.5kv

T. Flow: 0.2ml/min

B. Conc.: 0.05%FA in 30%H_2O/30%ACN/40%MeOH

KS-V PEPTIDE
The Peptide Experts

Certificate of Analysis

Product Information

Customer Code:	cmp8	KSVP Code:	KP3610-8
Lot No.:	20240720	Formula:	
M. W.:	685.91	Quantity:	5.0mg

Sequence:

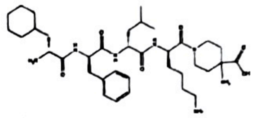

cmp8

Store at: -20℃

Analytical Results

Tests	Specifications	Results
Appearance	Report Result	white powder
Purity(HPLC)	≥95%	99.75%
MS(ESI)	Calc.685.91	Obs.685.85

Prepared By: 吴玲玲

Checked by: [illegible]

Date: 2024.07.22

KS-V PEPTIDE
The Peptide Experts

HPLC Report

Sample Information

KSVP Code: KP3610-8

Customer Code: cmp8
Lot No.: 20240720
Phase A: 0.1% TFA in deionized water
Phase B: 0.08% TFA in acetonitrile
Flow rate: 1.0mL/min
Wavelength: 214nm
Analytical column type: YMC-Pack ODS-A、120A、5um、4.6*250mm
Dissolution method: 100%H_2O
Inj. Volume: 10uL
Date Acquired: 20240723,15:44:03

Gradient:

Time（min）	Phase A	Phase B
0.01	95%	5%
2.00	95%	5%
22.00	1%	99%
30.00	Stop	

Chromatogram

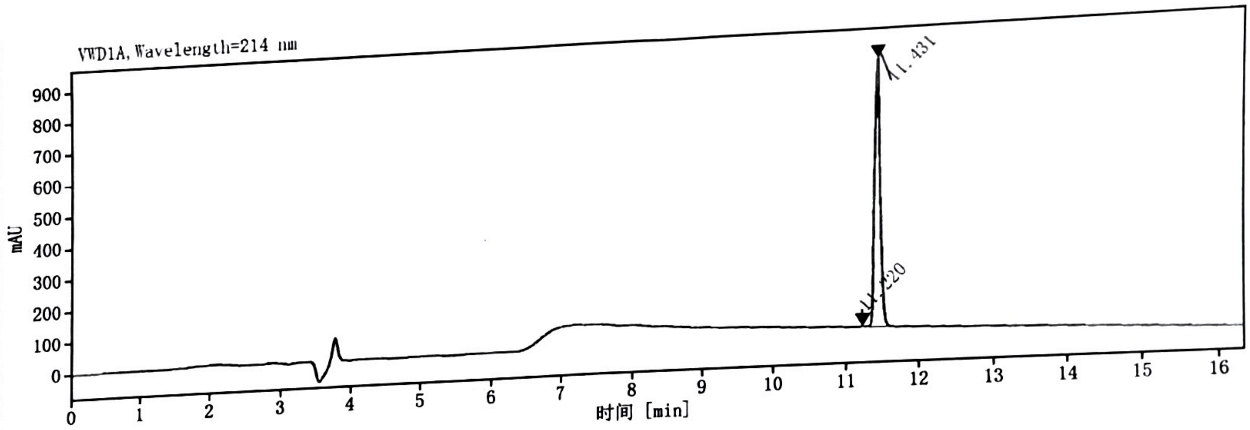

Peak	Ret time	Area	Area%	Height	Height%
1	11.220	11.1785	0.25	1.702	0.20
2	11.431	4531.2815	99.75	845.095	99.80

Mass Spectrometry Report

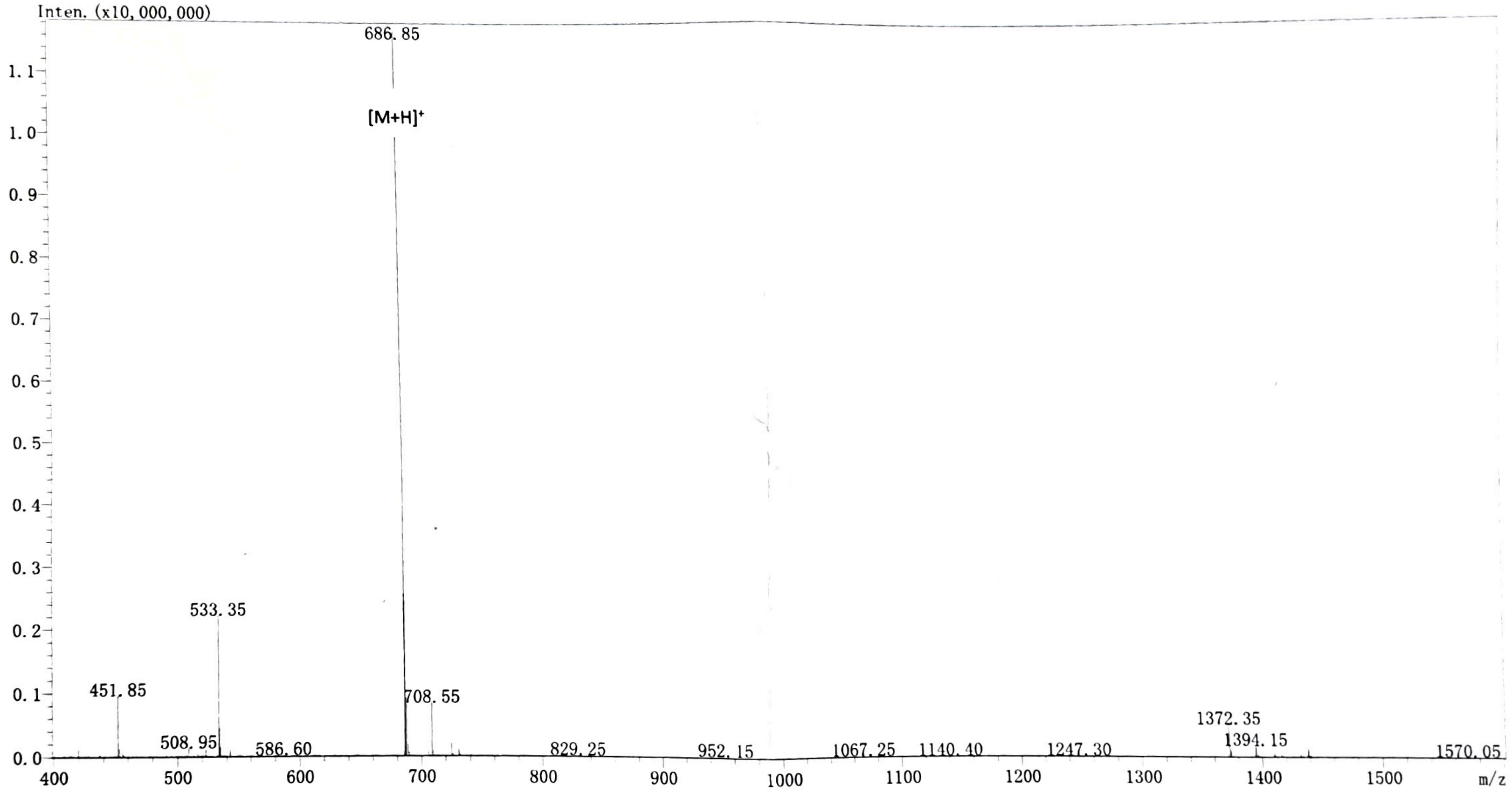

Sample Description

Analyzed date: 20240722,17:45
Analyst: WCG
Sample: cmp8;KP3610-8
M.W.: 685.91
Lot. No.: 20240720

Instrument SHIMADZU LCMS-2020
Probe: ESI
Nebulizer Gas Flow: 1.5L/min
CDL: -20.0v
CDL Temp.: 250°C
Block Temp.: 200°C

Probe Bias: +4.5kv
Detector: 1.5kv
T. Flow: 0.2ml/min
B. Conc.: 0.05%FA in 30%H_2O/30%ACN/40%MeOH
