# Supplementary material for: Rational design of a Kappa opioid receptor peptide agonist with attenuated β-arrestin signaling
Source: Nat Commun. 2026 Apr 14;17:5439. doi: 10.1038/s41467-026-71455-3 (PMC13279772; doi:10.1038/s41467-026-71455-3)
Supplement: Supplementary file 2 — Description of Additional Supplementary Files [file 41467_2026_71455_MOESM2_ESM.pdf]

Description of Additional Supplementary Files

Supplementary Video 1: Dynamic comparison of KOR-difelikefalin (cyan) and KOR-beta01 (magenta) during MD simulations. TM7 is highlighted using bold ribbons and darker coloring to emphasize its conformational movements.
