# Supplementary material for: Rational design of a Kappa opioid receptor peptide agonist with attenuated β-arrestin signaling
Source: Nat Commun. 2026 Apr 14;17:5439. doi: 10.1038/s41467-026-71455-3 (PMC13279772; doi:10.1038/s41467-026-71455-3)
Supplement: Supplementary file 4 — Reporting Summary [file 41467_2026_71455_MOESM4_ESM.pdf]

nature portfolio

Corresponding author(s): Changlin Tian
Yue Wang
Pan Shi
H.Eric Xu

Last updated by author(s): February 25, 2026

Reporting Summary

Nature Portfolio wishes to improve the reproducibility of the work that we publish. This form provides structure for consistency and transparency in reporting. For further information on Nature Portfolio policies, see our Editorial Policies and the Editorial Policy Checklist.

Statistics

For all statistical analyses, confirm that the following items are present in the figure legend, table legend, main text, or Methods section.

n/a	Confirmed	
☐	☒	The exact sample size (n) for each experimental group/condition, given as a discrete number and unit of measurement
☐	☒	A statement on whether measurements were taken from distinct samples or whether the same sample was measured repeatedly
☐	☒	The statistical test(s) used AND whether they are one- or two-sided<br>*Only common tests should be described solely by name; describe more complex techniques in the Methods section.*
☒	☐	A description of all covariates tested
☒	☐	A description of any assumptions or corrections, such as tests of normality and adjustment for multiple comparisons
☐	☒	A full description of the statistical parameters including central tendency (e.g. means) or other basic estimates (e.g. regression coefficient) AND variation (e.g. standard deviation) or associated estimates of uncertainty (e.g. confidence intervals)
☒	☐	For null hypothesis testing, the test statistic (e.g. F, t, r) with confidence intervals, effect sizes, degrees of freedom and P value noted<br>*Give P values as exact values whenever suitable.*
☒	☐	For Bayesian analysis, information on the choice of priors and Markov chain Monte Carlo settings
☒	☐	For hierarchical and complex designs, identification of the appropriate level for tests and full reporting of outcomes
☒	☐	Estimates of effect sizes (e.g. Cohen's d, Pearson's r), indicating how they were calculated

Our web collection on statistics for biologists contains articles on many of the points above.

Software and code

Policy information about availability of computer code

Data collection	Cryo-EM data collection on the Titan Krios using software of EPU software (FEI Eindhoven, Netherlands); NMR data collection on the Avance III 600 MHz Bruker using software of Topspin 4.4.0.
Data analysis	MotionCor2, crySPARC V4.5.3, RELION-3, Phenix 1.20,WinCoot 0.9.8.1, Pymol 2.0, ISOLDE-1.2, UCSF Chimera 1.17.1, UCSF ChimerX 1.3, Graphad Prism 9.0, TopSpin 4.4.0

For manuscripts utilizing custom algorithms or software that are central to the research but not yet described in published literature, software must be made available to editors and reviewers. We strongly encourage code deposition in a community repository (e.g. GitHub). See the Nature Portfolio guidelines for submitting code & software for further information.

Data

Policy information about availability of data

All manuscripts must include a data availability statement. This statement should provide the following information, where applicable:

- Accession codes, unique identifiers, or web links for publicly available datasets
- A description of any restrictions on data availability
- For clinical datasets or third party data, please ensure that the statement adheres to our policy

Source data are provided with this paper. The cryo-EM density maps and atomic coordinates have been deposited in the Electron Microscopy Data Bank (EMDB) and Protein Data Bank (PDB) under accession numbers EMD-68217 [https://www.ebi.ac.uk/pdbe/entry/emdb/EMD-68217] and 22ES [https://doi.org/10.2210/pdb22ES/pdb] for the difelikefalin-KOR-Gi complex, and EMD-68208 [https://www.ebi.ac.uk/pdbe/entry/emdb/EMD-68208] and 22EM [https://doi.org/10.2210/

pdb22EM/pdb] for the beta01-KOR-Gi complex. All the raw data from our molecular dynamic simulations have been uploaded to the public repository Zenodo (https://zenodo.org/records/17984759). The relevant raw data from each figure or table in the main manuscript and in the Supplementary Information are provided as a Source Data file.

Research involving human participants, their data, or biological material

Policy information about studies with human participants or human data. See also policy information about sex, gender (identity/presentation), and sexual orientation and race, ethnicity and racism.

Reporting on sex and gender	n/a
Reporting on race, ethnicity, or other socially relevant groupings	n/a
Population characteristics	n/a
Recruitment	n/a
Ethics oversight	n/a

Note that full information on the approval of the study protocol must also be provided in the manuscript.

Field-specific reporting

Please select the one below that is the best fit for your research. If you are not sure, read the appropriate sections before making your selection.

- [x] Life sciences
- [ ] Behavioural & social sciences
- [ ] Ecological, evolutionary & environmental sciences

For a reference copy of the document with all sections, see nature.com/documents/nr-reporting-summary-flat.pdf

Life sciences study design

All studies must disclose on these points even when the disclosure is negative.

Sample size	For cryo-EM data, images were collected until the resolution and structural reconstruction converges. For all the cellular functional assay, three independent experiments were conducted. For animal experiments, each experimental group should consist of no fewer than 6 mice. The sample size is sufficient for statistical analysis.
Data exclusions	During cryoEM data processing, only the particles that give rise to homogeneous density were selected and used in the final reconstruction.
Replication	The functional assay were performed at least three independent experiments with technical repeats. The purifications of protein complexes at replication were successful. The number of replicates is noted in the figure legends or methods section.
Randomization	All animals were numbered in advance and grouped randomly.
Blinding	Blinding was not applicable except for the animal behavior tests. For these tests, the researcher performing the behavioral measurements was blinded to group allocation. Another investigator, who was not involved in the assessments, was responsible for weighing, dispensing, and dosing, and the animals were identified only by numbers.

Reporting for specific materials, systems and methods

We require information from authors about some types of materials, experimental systems and methods used in many studies. Here, indicate whether each material, system or method listed is relevant to your study. If you are not sure if a list item applies to your research, read the appropriate section before selecting a response.

Materials & experimental systems

n/a	Involved in the study	
[ ]	[x]	Antibodies
[ ]	[x]	Eukaryotic cell lines
[x]	[ ]	Palaeontology and archaeology
[ ]	[x]	Animals and other organisms
[x]	[ ]	Clinical data
[x]	[ ]	Dual use research of concern
[x]	[ ]	Plants

Methods

n/a	Involved in the study	
[x]	[ ]	ChIP-seq
[ ]	[x]	Flow cytometry
[x]	[ ]	MRI-based neuroimaging

Antibodies

Antibodies used	rat PE anti-Flag tag antibody (1:200, Biolegend, 637309)
Validation	The antibodies were well characterized and applied according to data sheet information details. https://www.biolegend.com/en-us/products/pe-anti-dykddddk-tag-antibody-9383

Eukaryotic cell lines

Policy information about cell lines and Sex and Gender in Research

Cell line source(s)	Sf9 cell line was purchased from Life Technologies; HEK293T(ATCC, #CRL-3216).
Authentication	Details on cell line authentication are not provided.
Mycoplasma contamination	No mycoplasma contamination were detected.
Commonly misidentified lines (See ICLAC register)	No commonly misidentified lines were used.

Animals and other research organisms

Policy information about studies involving animals; ARRIVE guidelines recommended for reporting animal research, and Sex and Gender in Research

Laboratory animals	Male KM mice (6-8 weeks old) were obtained from Jiangsu Huachuang Xinnuo Pharmaceutical Technology Co., Ltd. Male C57BL/6J mice (6-8 weeks old) were obtained from Hangzhou Ziyuan Experimental Animal Technology Co., Ltd. Male ICR mice (6-8 weeks old) were obtained from Hangzhou Ziyuan Experimental Animal Technology Co., Ltd. The mice were housed at the Hefei Institutes of Physical Science, Chinese Academy of Sciences facility under standard laboratory conditions. These conditions included a 12-hour light/dark cycle, a temperature of 20-22°C, and humidity ranging from 40% to 70%. The mice had ad libitum access to water and food throughout the study. In this study, all experiments were specifically designed to minimize the number of animals used and were approved by the Animal Ethics Committee of the Hefei Institutes of Physical Science, Chinese Academy of Sciences and were conducted in accordance with the National Institutes of Health Guide for the Care and Use of Laboratory Animals (DWLL (E)-2024-06).
Wild animals	The study did not involve wild animals.
Reporting on sex	According to previous experimental method, the study used male animals in the behavioral tests.
Field-collected samples	No field-collected samples were used.
Ethics oversight	The experiments were approved by the Animal Ethics Committee of the Hefei Institutes of Physical Science, Chinese Academy of Sciences and were conducted in accordance with the National Institutes of Health Guide for the Care and Use of Laboratory Animals (DWLL (E)-2024-06).

Note that full information on the approval of the study protocol must also be provided in the manuscript.

Plants

Seed stocks	n/a
Novel plant genotypes	n/a
Authentication	n/a

Flow Cytometry

Plots

Confirm that:

- [ ] The axis labels state the marker and fluorochrome used (e.g. CD4-FITC).
- [ ] The axis scales are clearly visible. Include numbers along axes only for bottom left plot of group (a 'group' is an analysis of identical markers).
- [ ] All plots are contour plots with outliers or pseudocolor plots.
- [ ] A numerical value for number of cells or percentage (with statistics) is provided.

Methodology

Sample preparation	Sample preparation listed in Methods.
Instrument	BD Accuri C6 (BD Biosciences)
Software	BD Accuri C6 software 1.0.264.21
Cell population abundance	Approximately 10,000 cellular events were collected and the total fluorescence intensity of positive expression cellpopulation was calculated.
Gating strategy	Gating was determined by the PE fluorescence intensity to differentiate positive cells and all other cells

- [x] Tick this box to confirm that a figure exemplifying the gating strategy is provided in the Supplementary Information.
