# Supplementary material for: Rational design of a Kappa opioid receptor peptide agonist with attenuated β-arrestin signaling
Source: Nat Commun. 2026 Apr 14;17:5439. doi: 10.1038/s41467-026-71455-3 (PMC13279772; doi:10.1038/s41467-026-71455-3)
Supplement: Supplementary file 5 — Transparent Peer Review File [file 41467_2026_71455_MOESM5_ESM.pdf]

nature portfolio

Peer Review File

Rational design of a kappa opioid receptor peptide agonist with attenuated β-arrestin signaling

Corresponding Author: Professor Changlin Tian

This file contains all reviewer reports in order by version, followed by all author rebuttals in order by version.

Version 0:

Reviewer comments:

Reviewer #1

(Remarks to the Author)
In this manuscript, Zhang et al explore the binding mode of difelikefalin an FDA-approved unbiased KOR agonist. Using analysis of a cryo-EM structure of difelikefalin-bound KOR, the authors, via point mutagenesis identify Y320 as a residue important in producing the signaling preference of the peptide. The authors, then synthesize 50 peptides or peptidomimetics, ultimately identifying beta01, which has significant G-protein preference relative to difelikefalin. Tested in a range of efficacy and on-target side effect animal models, the authors show beta01 to retain antinociceptive and antipuritic efficacy while showing limited adverse effects within the tested dose range. Cryo-EM, NMR spectroscopy and MD simulations of beta01-bound KOR suggest that the biased profile of beta01 may arise by stabilizing a conformation where the intracellular opening that facilitates interactions with the G-protein and beta-arrestin has increased space, which may reduce the affinity for this site to engage with arrestins.
The article was well written and the hypothesis was clearly presented and tested. I have some minor issues that could be addressed.

Why did the authors alternate between using Kunming and C57Bl/6 mice?

Why did the authors opt to use an a priori strategy of eliminating the highest and lowest values from statistical analysis? Given the relative small cohort sizes, this may remove non-outliers. Additionally, it appears that for the rotarod this strategy is not used. Could the authors show what the data looks like if these values are retained, or if outliers are only removed for example with a Grubb’s test or similar test.

Can the authors comment if some of the other compounds that display G-protein bias (Figure 2B) have a similar modelled MoA as beta01? Related, have the authors modeled compound 13 (or multiple b-arrestin biased agonist) to see if this narrows the intracellular pocket in line with their beta01 hypothesis

Reviewer #2

(Remarks to the Author)
Summary
A high-resolution structure of the difelikefalin-KOR-Gi complex was determined and key ligand interacting residues were screened for their role in Gi or arrestin signaling. Y320 was identified as a key residue involved in arrestin signaling. Analogs of difelikefalin were synthesized with subsitutions of the Y320 interacting residue. One ligand showed a strong bias for Gi activation.
Subsequently a high-resolution structure of this ligand-KOR-Gi complex was solved and a model of the molecular mechanism for the biased signaling was deducted. In addition, the ligand was studied in mouse behavioral assays, which showed reduced side effects.

I recommend the manuscript with minor revisions for publication in Nature Communications.

Significance
It is a significant work in the field of GPCR research. Especially the subtle differences in the structure of the biased ligand

and resulting changes in the Gi binding pocket will help to further understand GPCR and especially KOR signaling.
It is not the first work describing the molecular mechanism of biased signaling at the kappa opioid receptor. But the structures and the new ligand with mouse characterization are novel.

Data and methodology
The manuscript is clearly written the figures are well designed and easy to understand and the methods include everything to reproduce the experiments.

Validity
The structure determination, the biochemical assays, the ligand synthesis, the mouse studies and the MD simulation look valid, and the deducted conclusions seem reasonable to me.

My only concern is the interpretation of the NMR data. Usually 13C-Met labeling results in a receptor where all Met are labeled. With these multiple probes a receptor wide dynamic can be observed. Assignments are usually obtained by point mutations. The construct used in this study has 12 Met. But for the NMR measurements all Met were mutated to Leu and an additional L333M mutation. With this system they do not have problems with assignments and peak overlap, but they investigate a highly mutated receptor. For example M142 is in the ligand binding pocket and M112 is directly next to the important Y320. Met and Leu are very similar in volume, but they have a different hydrophobicity. In Ext. Data Fig 11 b they show that this receptor still activates Gi in a wt like way. But they do not show if this mutant can also recruit arrestin. They clearly show that naltrexone shows only an “inactive state”. And with difelikefalin they mainly see an “active state”. But should difelikefalin not show a Gi and an arrestin state? In the beta01 bound state they see 3 different peaks where they assign the additional one to a “G bias state”. To confirm this hypothesis, they could record a spectra in a Gi or arrestin bound state to see which peak is really involved in Gi or arrestin binding. For me it is unclear if this peak is a real feature of an intermediate biased state or just an artifact from the mutated receptor.
Additionally, they compare these 3 different peaks of KORΔM-M333 to 3 different rotameric states of Chi2 of KOR-L333 in the MD simulation. Not only are these 2 completely different proteins but also 2 different time scales. A slow exchange in NMR can be observed if an additional peak for one atom is observed, while in fast exchange the dynamics are averaged and only one peak can be observed. While sidechain dynamics are usually in the ps-ns range, slow exchange in NMR is in the µs-s range. Because of this I do not think one can make a correlation.
From the MD simulation it can be observed that the S2 rotameric state has a higher population and that the RMSD of L333 is greater in the beta01 state. This fits with the result from the structure that beta01 has a bigger cavity between helix 6 and 7 and that this leads to biased signaling. I am not sure if the NMR data really supports this hypothesis.

Suggested improvements
In line 318 they should clearly state that they used the KORΔM mutant. And they should show how this mutant can recruit arrestin.

The difference in the dynamic range of the MD and NMR results needs to be reanalyzed.

I think a more elegant way to use NMR would be to use the normal KOR and not the KORΔM receptor with 13C-Met labeling. A complete assignment would be difficult, but maybe just a difelikefalin, a beta01 and a naltrexone bound spectra could give insight into a different ratio of inactive, active and intermediate states.

I am a great fan of NMR but somehow I feel without these ambiguous NMR results the overall statement of the paper would be more convincing.

Reviewer #3

(Remarks to the Author)
Zhang et al. combine in vitro assays, cryo-EM structures, and molecular simulations to investigate the structural foundations of G-protein bias at the κ-opioid receptor (KOR). Given that, despite some controversies, G-protein bias has been associated with favorable therapeutic outcomes for opioid receptor–targeting drugs, this study is timely, novel, and of clear scientific relevance.

However, before publication, I recommend that the authors address the following points:

1.)The authors present an alanine mutation at position Y7.43, emphasizing its role in bias modulation. To strengthen this conclusion, I recommend also generating Y7.43F and possibly Y7.43L mutants, as these substitutions maintain similar physicochemical properties and would help disentangle steric from polar effects.

2)The MD trajectories are not currently available in any repository. I would like to review these data before final acceptance. Moreover, the authors should deposit the MD simulations in a public repository (e.g., Zenodo, OSF, or GPCRmd) to ensure reproducibility and transparency. How was the protonation of tiratable residues assigned for MD simulations?

3)I recommend performing MD simulations of the Y7.43A mutant in complex with both ligands, followed by the same analyses used for the wild-type receptor—particularly monitoring the TM7–TM6 distance and other molecular features highlighted in the manuscript.

4)In Figure 4, I suggest including a supplementary panel showing conformational clustering of the L338 residue. This would help visualize the local conformational diversity and support the interpretations drawn from the main text.

5)Did the authors attempt to back-calculate NMR spectra from their MD simulations? Comparing simulated and experimental spectra (if available) could provide valuable validation of the proposed conformational states.

Reviewer #4

(Remarks to the Author)
Overall, this is clean and well written manuscript that details peripherally restricted KOR agonist Korsuva used in the treatment of pruritus. The authors interrogate mutations surrounding the orthosteric binding pocket and identify a modified Korsuva molecule (b01) that exhibits G protein over bArrestin bias. Structures with this new molecule show atomic details of enhanced binding. The authors follow up on this new compound in mice models showing “adverse” effects with this new biased molecule are mitigated, presumably through its greater G-protein bias. Additionally, NRM studies find along with MD studies that suggest this new compound appears to stabilizes an intermediate intracellular receptor conformation, that perhaps appears to modify the intracellular cavity. To improve transparency, the authors are encouraged to acknowledge data limitations, provide additional panels demonstrating map quality in critical regions, and supplement histogram plots with box and whisker overlays.

Major:
1. The comparison between b01 and difelikefalin is awkward due to different construct preps (LgBit fusion for b01). The authors must explicitly address this in the main text especially when discussing intracellular cavity differences.
2. The larger intracellular cavity (fig 3h and page 11, line 280) is small, especially (1) given the often-poorer density of TM7/TM8 and(2) the beta01 bound complex employed a LgBit (presumably to this H8) while the difelikefalin did not. I would again strongly advise specifically addressing this in the results, temper the observations with this caveat and state the rationale to perform additional MD and NMR to investigate these observations using orthogonal methodologies.
3. The MD RMSD fluctuations appear within error and are complicated by the different construct preps. That is, starting models could bias MD observations. Perhaps, if the authors wish to be more thorough starting with essentially the same atomic model with the two different ligands bound would help make this data more compelling (or less).
4. In regards to above please clarify methods for MD simulations to address this.
5. The RMSD fluctuations page 12 line 302-307 seem to be within error; perhaps a movie would be more beneficial. Fig 4g and Fig 10 (and all other MD histograms) must be co-plotted with the current histogram in addition to an overlay of a box and whisker plot.
6. Much of the difference between the two models appears to be around the TM7/8 region with respect to the larger cavity size. If the authors wish to demonstrate these differences an additional main figure panel showing density of TM7/TM8 in a side-by-side view for each KOR-ligand complex with transparent local resolution mapped to the surface of these two maps/models is critical for reader evaluation. This would be helpful to illustrate key structural differences and local resolution and map quality for this region.
7. Comparing the two models derived from the cryo-EM maps the intracellular cavity difference is subtle the numbers on page 12 line 296-297 show a small difference within error. Given the difference in the two preparations this does cause some concern. Although, the NMR experiments help assuage these concerns
8. Please address the extra density between beta01’s N-terminal phenylalanine and Y320. Are multiple binding poses congruent with the ensemble map density. How much different would the beta01 pose vs difelikefalin be line with this alternative pose? How would that alter Y320? Is the Y320 in beta01 strong enough to make claims about relative changes in side chain position, the density seem poor for this residue for the beta01 map.
9. How do mutations around beta01 differential pocket affect binding. For example Y320A? Are these results consistent with two binding poses for beta01, given the unaccounted for density?

Minor:
Figure 5 “sodium binding site is odd” since this is in part between 2.50 and other residues in the inactive state. 4N6H of DOR shows interaction of sodium with 3.35,3.39,6.48 in their 1.8A inactive state structure. Perhaps putative sodium coordinating residue would be more appropriate language or omit.

Local resolution map to the surface of the ligand in Extended Data figure 1 and 8 would be helpful

The heat map key for local resolution lacks units and coloring between 1 and 8 is different, which is odd given the high quality and uniformity in the rest of the manuscript.

The “dust” could be omitted from 1d to aid in visualization of pertinent density.

Statistics lacking on data tables.

Version 1:

Reviewer comments:

Reviewer #1

(Remarks to the Author)
The authors, adequately responded to my questions, as well as those of the other reviewers and adjusted their statements and interpretations appropriately.

Reviewer #2

(Remarks to the Author)
The changes in the manuscript regarding my comments are acceptable.

Reviewer #3

(Remarks to the Author)
The authors have succesfully adressed all of my comments. I congratulate them on the ammount of work and detail when adressing issues hilighted by me

Reviewer #4

(Remarks to the Author)
The authors have addressed my concerns.

Open Access This Peer Review File is licensed under a Creative Commons Attribution 4.0 International License, which permits use, sharing, adaptation, distribution and reproduction in any medium or format, as long as you give appropriate credit to the original author(s) and the source, provide a link to the Creative Commons license, and indicate if changes were made.
In cases where reviewers are anonymous, credit should be given to 'Anonymous Referee' and the source.
The images or other third party material in this Peer Review File are included in the article's Creative Commons license, unless indicated otherwise in a credit line to the material. If material is not included in the article's Creative Commons license and your intended use is not permitted by statutory regulation or exceeds the permitted use, you will need to obtain permission directly from the copyright holder.
To view a copy of this license, visit https://creativecommons.org/licenses/by/4.0/

Manuscript ID: NCOMMS-25-71769-T

Title: Rational design of a kappa opioid receptor (KOR) peptide agonist with attenuated β-arrestin signaling

We thank the reviewers for the tremendous time and effort they devoted to evaluating our manuscript. We are especially grateful for their positive assessment of the quality and significance of this work, as well as for the thoughtful, constructive suggestions that have helped us substantially improve the clarity, rigor, and presentation of the study. In response to the reviewers' comments, we have revised the manuscript extensively and added new analyses and supporting data, including strengthened validation and clarification of the constructs used for NMR/structural studies, improved local-resolution visualization over ligand densities, rebuilt and expanded molecular dynamics simulations with matched starting conformations and replicate trajectories, and additional presentation/statistical details to enhance transparency and reproducibility. We hope these revisions address all concerns and further strengthen the overall conclusions. Please find our point-by-point responses below. Reviewer comments are provided in **black** font, and our responses are in **blue** font.

Reviewer #1 (Remarks to the Author):

In this manuscript, Zhang et al explore the binding mode of difelikefalin an FDA-approved unbiased KOR agonist. Using analysis of a cryo-EM structure of difelikefalin-bound KOR, the authors, via point mutagenesis identify Y320 as a residue important in producing the signaling preference of the peptide. The authors, then synthesize 50 peptides or peptidomimetics, ultimately identifying beta01, which has significant G-protein preference relative to difelikefalin. Tested in a range of efficacy and on-target side effect animal models, the authors show beta01 to retain antinociceptive and antipuritic efficacy while showing limited adverse effects within the tested dose range. Cryo-EM, NMR spectroscopy and MD simulations of beta01-bound KOR suggest that the biased profile of beta01 may arise by stabilizing a conformation where the intracellular opening that facilitates interactions with the G-protein and beta-arrestin has increased space, which may reduce the affinity for this site to engage with arrestins.
The article was well written and the hypothesis was clearly presented and tested. I have some minor issues that could be addressed.

Response:
We appreciate the reviewer's positive comments and have tried our best to provide responses to address the reviewer's questions or concerns.

Point 1: Why did the authors alternate between using Kunming and C57Bl/6 mice?

Response:

We thank the reviewer for this insightful question.

We selected Kunming Mice as the primary strain for behavioral assessments (including antinociception, spontaneous locomotion, elevated plus maze, and tail suspension tests), because Kunming mice are widely used in preliminary pharmacodynamic and safety evaluations. They are cost-effective, robust, highly fecund, and have demonstrated stable and reliable responses across a variety of pharmacological behavioral models (e.g., Xu, Kangtai et al., Neuropharmacology, 2024, 261:110157. Kong, Linghui et al., J Med Chem, 2022, 65(15):10377-10392).

For the chloroquine-induced scratching test, we initially attempted to establish the model in Kunming mice. However, during pilot studies, Kunming mice exhibited a weak scratching response to chloroquine. Therefore, the C57BL/6J strain, which is well-established and highly sensitive in this model, was employed for the pruritus assay (Wei, Yuan-Yuan et al., Acta pharmacologica Sinica, 2022, 43(6): 1372-1382).

Notably, all intergroup comparisons were strictly performed within the same mouse strain. Therefore, our primary conclusions regarding the superior efficacy and safety profile of beta01 are not much affected by the two strains of mice.

Point 2: Why did the authors opt to use an a priori strategy of eliminating the highest and lowest values from statistical analysis? Given the relative small cohort sizes, this may remove non-outliers. Additionally, it appears that for the rotarod this strategy is not used. Could the authors show what the data looks like if these values are retained, or if outliers are only removed for example with a Grubb's test or similar test.

Response:

We thank the reviewer for raising this insightful point.

To address this concern, we have re-analyzed the full dataset for all behavioral assays (scratching, writhing, open field, rotarod, elevated plus maze, and tail suspension tests) using a unified, statistically rigorous method. We used Grubbs' test ($\alpha = 0.05$) as our pre-defined criterion for identifying outliers. All individual data points are provided in the Source Data.

It is crucial to emphasize that after applying this objective outlier method, the primary statistical conclusions and significances for all our behavioral experiments remain unchanged.

In the revised manuscript, we have updated Fig.2d and supplementary Fig.7, and added the following sentence in the methods section.

(P.27, Lines 776,789 and 799; P.28, Lines 811 and 823; P.29, Line 835).

"*Data points were excluded from the analysis only if they were identified as statistical outliers by Grubbs' test ($\alpha = 0.05$).*"

We thank the reviewer again for helping us improve the statistical rigor of our work.

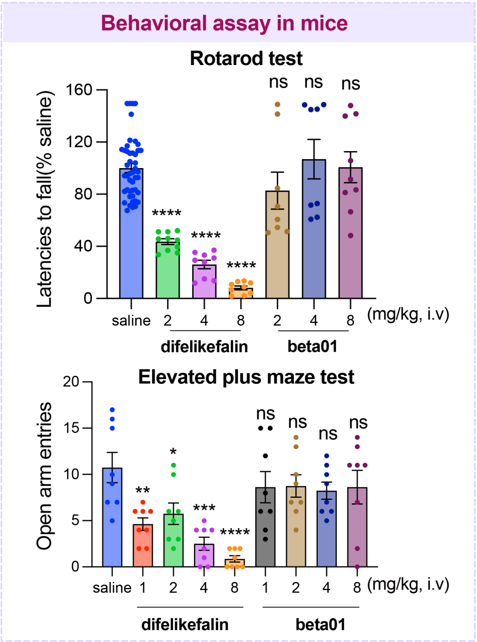

Fig. R1 (Fig. 2d in revised manuscript) | Sedation and anxiogenic effect was tested on the rotarod test and the elevated plus maze respectively.

Data are presented as the mean ± S.E.M. from at least 6 mice/data point. Data points were excluded from the analysis only if they were identified as statistical outliers by Grubbs' test (α = 0.05). * $P < 0.05$, ** $P < 0.01$, *** $P < 0.001$, **** $P < 0.0001$.

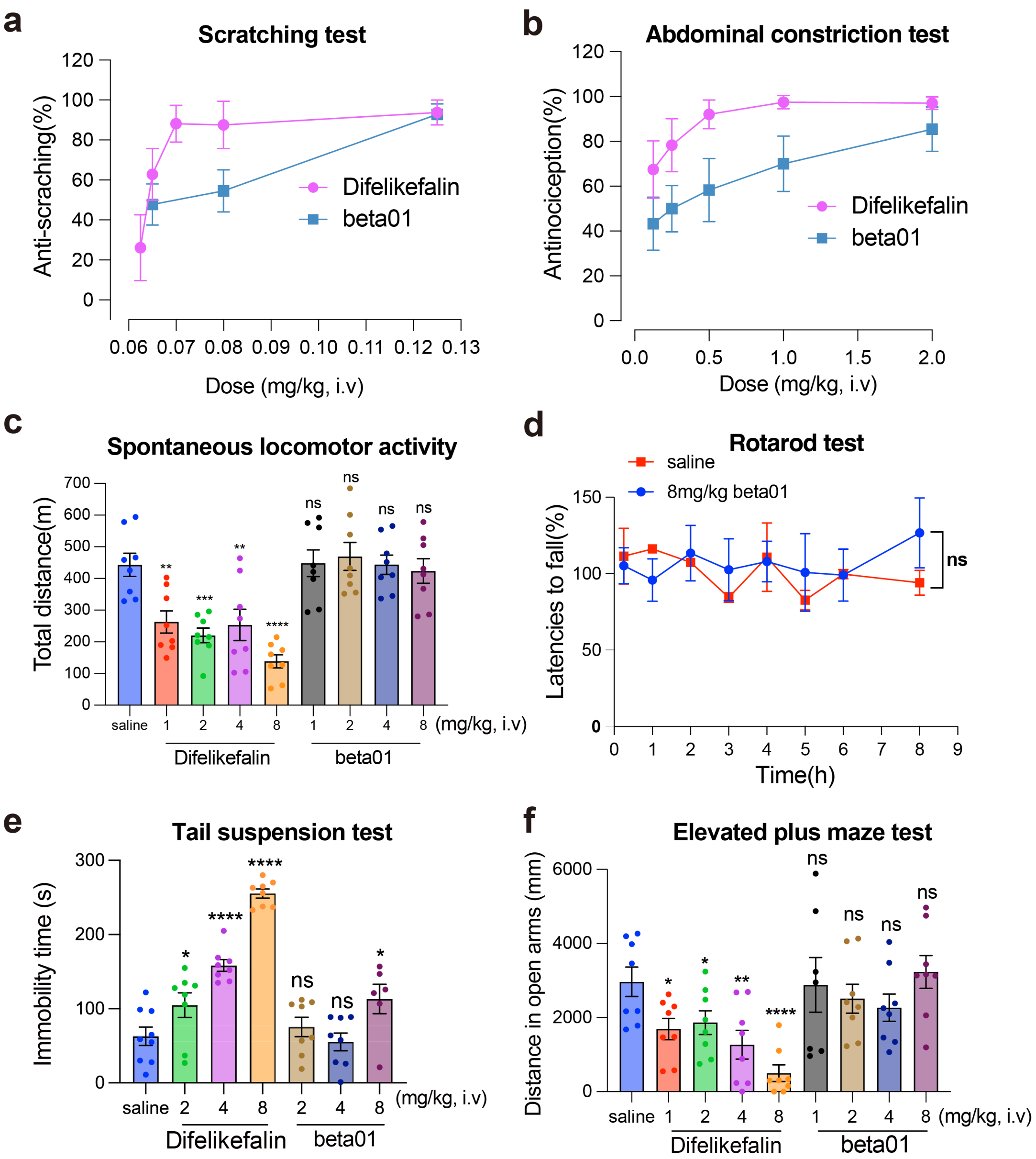

Fig. R2 (Supplementary Fig. 7 in revised manuscript) | Behavioral studies of difelikefalin and beta01 in animal models.

a, The antipruritic effects of difelikefalin and beta01 in response to administration of chloroquine diphosphate. **b,** Antinociceptive effects of difelikefalin and beta01 were evaluated in the acid writhing test. **c,** Effect of difelikefalin and beta01 on spontaneous activities in mice. **d,** Time course for the sedation effect of beta01 at the dose of 8mg/kg weight in the rotarod test. Each value represents the mean ± SEM from at least six mice. ns means no significant difference. **e,** Immobility time statistics of mice treated with different doses of difelikeflain and beta01 in the tail suspension test. **f,** The distance traveled within the open arms of mice during the elevated plus maze test. Data are presented as the mean ± S.E.M. from at least 6 mice/data point. Data points were excluded from the analysis only if they were identified as statistical outliers by Grubbs' test (α = 0.05). * $P < 0.05$, ** $P < 0.01$, *** $P < 0.001$, **** $P < 0.0001$.

Point 3: Can the authors comment if some of the other compounds that display G-protein bias (Fig 2B) have a similar modelled MoA as beta01? Related, have the authors modeled compound 13 (or multiple b-arrestin biased agonist) to see if this narrows the intracellular pocket in line with their beta01 hypothesis.

Response:

We thank the reviewer for this insightful suggestion.

To quick evaluate the G proteins bias or KOR1 by different compounds, MD simulations were conducted for the KOR1 structural responses upon binding of different compounds.

In addition to difelikefalin and beta01, we selected cmp7 (G protein biased) and cmp13 (β-arrestin biased) for MD simulations. To ensure a consistent starting conformation of KOR across all systems, we adopted the recommendation of another reviewer and used the same initial KOR structure for each simulation (**Fig. R3**). The ligand binding poses were maintained in a consistent manner via editing on site.

As shown in the **Fig. R3**, compared with the difelikefalin system, cmp13 reduced the distance between TM6 and TM7 (8.83 ± 1.57 Å). This reduction is accompanied by a decrease in SASA of the G protein binding pocket residues (1656 ± 116 $Å^2$), indicating that cmp13 also narrowed the intracellular pocket of KOR like difelikefelin. In contrast, cmp7 increased the TM6-TM7 distance to 12.23 ± 1.87 Å and enlarged the SASA into 1780 ± 136 $Å^2$, reaching a level comparable to that observed in the beta01 system (12.22 ± 2.29 Å and 1801 ± 117 $Å^2$).

Collectively, these results demonstrate that cmp7 and cmp13 differentially modulate the intracellular pocket of KOR in a manner consistent with their respective signaling biases, further supporting the mechanistic hypothesis derived from the β01 data.

The conformational alterations for the intracellular domain of KOR induced by difelikefalin, beta01, cmp7 and cmp13 in molecular dynamics were shown in the **new Supplementary Fig. 12** and are discussed in the results section.

(P.12-13, Lines 316-325).

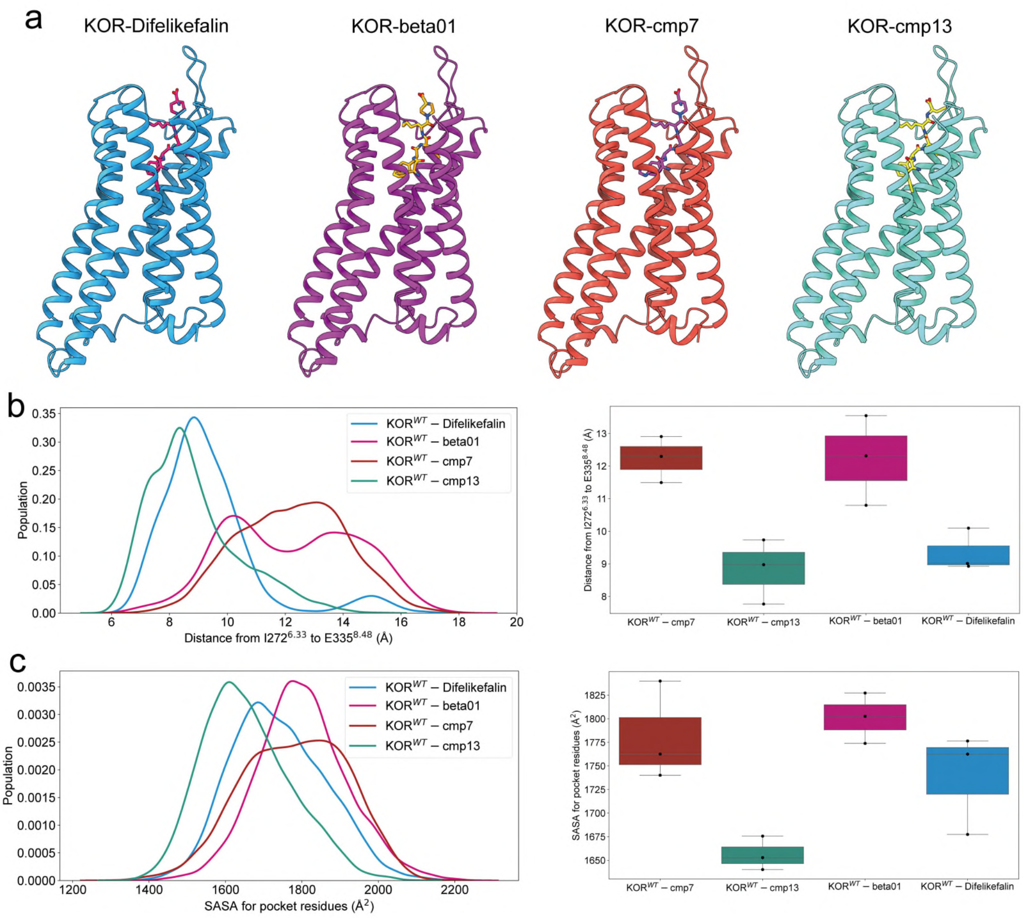

Fig. R3 (Supplementary Fig. 12 in revised manuscript) | Conformational alterations for the intracellular domain of KOR induced by difelikefalin, beta01, cmp7 and cmp13 in molecular dynamics. a, the initial structures of simulation systems KOR-difelikefalin, KOR-beta01, KOR-cmp7 and KOR-cmp13. **b,** the distribution of the Cα distance between I272$^{6.33}$ and E335$^{8.48}$ for the KOR-difelikefalin, KOR–beta01, KOR-cmp7 and KOR-cmp13 systems in MD, illustrated using a KDE plot (left) to show the probability density and a boxplot (right) to summarize statistical variation between the two systems. **c,** the distribution of SASA values for pocket residues in the KOR-difelikefalin, KOR–beta01, KOR-cmp7 and KOR-cmp13 systems in MD, illustrated using a KDE plot (left) and a boxplot (right).

Reviewer #2 (Remarks to the Author):

Summary

A high-resolution structure of the difelikefalin-KOR-Gi complex was determined and key ligand interacting residues were screened for their role in Gi or arrestin signaling. Y320 was identified as a key residue involved in arrestin signaling. Analogs of difelikefalin were synthesized with subsitutions of the Y320 interacting residue. One ligand showed a strong bias for Gi activation. Subsequently a high-resolution structure of this ligand-KOR-Gi complex was solved and a model of the molecular mechanism for the biased signaling was deducted. In addition, the ligand was studied in mouse behavioral assays, which showed reduced side effects.

I recommend the manuscript with minor revisions for publication in Nature Communications.

Response:

We appreciate the reviewer's positive comments.

Significance

It is a significant work in the field of GPCR research. Especially the subtle differences in the structure of the biased ligand and resulting changes in the Gi binding pocket will help to further understand GPCR and especially KOR signaling. It is not the first work describing the molecular mechanism of biased signaling at the kappa opioid receptor. But the structures and the new ligand with mouse characterization are novel.

Data and methodology

The manuscript is clearly written the Figs are well designed and easy to understand and the methods include everything to reproduce the experiments.

Validity

The structure determination, the biochemical assays, the ligand synthesis, the mouse studies and the MD simulation look valid, and the deducted conclusions seem reasonable to me.

Point 1: My only concern is the interpretation of the NMR data. Usually 13C-Met labeling results in a receptor where all Met are labeled. With these multiple probes a receptor wide dynamic can be observed. Assignments are usually obtained by point mutations. The construct used in this study has 12 Met. But for the NMR measurements all Met were mutated to Leu and an additional L333M mutation. With this system they do not have problems with assignments and peak overlap, but they investigate a highly mutated receptor. For example M142 is in the ligand binding pocket and M112 is directly next to the important Y320. Met and Leu are very similar in volume, but they have a different

hydrophobicity. In Ext. Data Fig 11 b they show that this receptor still activates Gi in a wt like way. But they do not show if this mutant can also recruit arrestin.

Response:

We thank the reviewer for the valuable suggestion.

We absolutely agree with the reviewer on the concern regarding the functional integrity of the NMR constructs.

Our initial NMR experiments were indeed performed on the wild-type KOR. However, as the reviewer and we anticipated, the presence of 12 methionine residues resulted in severe spectral overlap, making site-specific assignment and interpretation almost impossible (**Fig.R4**). This necessitated the mutation strategy, leading to the construction of the $KOR^{\Delta M}$-L333M mutant, which yielded clean, interpretable spectra.

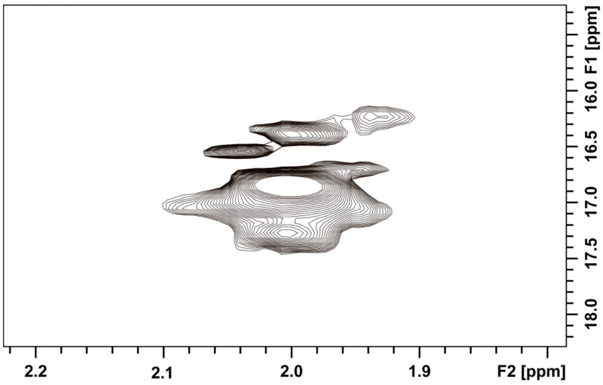

Fig. R4 | ^{1}H-^{13}C HMQC spectra of the [methyl-^{13}C-Met] KOR-9M in the naltrexone bound.

Additionally, our preliminary data shows that the mutant retains the ability to activate G protein. Following the reviewer's suggestion, we have performed the β-arrestin2 recruitment assay. The results demonstrate that the $KOR^{\Delta M}$-L333M mutant retains the capacity to recruit β-arrestin2 (**Fig.R5**).

A key strength of our experimental design is that all NMR data were collected using the identical mutant receptor. Thus, spectral differences, such as the unique $M333^{G\text{-}bias}$ peak observed with beta01, can be attributed to from distinct ligand pharmacology rather than receptor variation. This internal control confidently establishes that beta01 stabilizes a receptor conformation, which is distinct from that induced by the balanced agonist difelikefalin.

The functional validation of the $KOR^{\Delta M}$-L333M mutant for both Gi activation and β-arrestin2 recruitment is now provided in Supplementary Fig. 14b.
We have clarified the use of this mutant in the main text.

(P.13, Line 342-345)

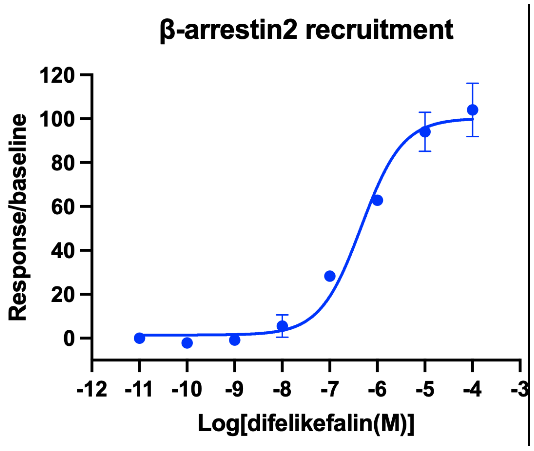

Fig. R5 (Supplementary Fig. 14b in revised manuscript) | β-arrestin2 recruitment of KOR$^{\Delta M}$-L333M.

Point 2: They clearly show that naltrexone shows only an “inactive state”. And with difelikefalin they mainly see an “active state”. But should difelikefalin not show a Gi and an arrestin state? In the beta01 bound state they see 3 different peaks where they assign the additional one to a “G bias state”. To confirm this hypothesis, they could record a spectrum in a Gi or arrestin bound state to see which peak is really involved in Gi or arrestin binding. For me it is unclear if this peak is a real feature of an intermediate biased state or just an artifact from the mutated receptor.

Response:

We thank the reviewer for this valuable question.

We agree that acquiring Gi- or arrestin-saturated spectra would represent a good standard for assigning the putative "G-protein-biased" peak.

Regrettably, the generation of ternary complexes (KOR-difelikefalin-Gi or β-arrestin) that meet the stringent criteria of stability, monodispersity, and sufficient concentration for NMR analysis has proven technically challenge.

Therefore, we have softened and qualified our interpretations of the NMR data in the revised manuscript.

(P.3, Lines 53-55; P.14, Lines 360-362)

Point 3: Additionally, they compare these 3 different peaks of KORΔM-M333 to 3 different rotameric states of Chi2 of KOR-L333 in the MD simulation. Not only are these 2 completely different proteins but also 2 different time scales. A slow exchange in NMR can be observed if an additional peak for one atom is observed, while in fast exchange the dynamics are averaged and only one peak can be observed. While sidechain dynamics are usually in the ps-ns range, slow exchange in NMR is in the µs-s range. Because of this I do not think one can make a correlation.

Response:

Thanks for this valuable suggestion.

We agree that the three-peak pattern observed in the NMR spectra reflects slow exchange on the μs-s timescale, which is not directly comparable to the ps-ns χ2 rotameric dynamics observed in MD simulations.

We appreciate the reviewer's point that a one-to-one correlation between NMR peak multiplicity and χ2 rotameric states is NOT justified, particularly given the different timescales and that $KOR^{\Delta M}$-M333 and KOR-L333 represent two different constructs.

Our original intention was not to assign each NMR peak with a specific rotameric state, but rather to illustrate that multiple conformational substates of 333 exist and that both NMR and MD suggest conformational heterogeneity at this residue.

To avoid misinterpretation, we have revised the words in the manuscript to remove any implication of a direct one-to-one correlation, and clarify that MD supports the existence of side chain conformational variability but does not resolve the slower exchange processes detectable by NMR.

Besides, we performed tICA (time-lagged Independent Component Analysis) based on the globally aligned heavy-atom coordinates of $L333^{7.56}$. As depicted in the **Fig. R6**, there were two distinct conformational clusters in the KOR-difelikefalin system (S1 and S2), whereas three sets of conformational clusters (S1, S2 and S3) were observed in the KOR-beta01 system, highlighting the pronounced differences in the conformational states of L333 between binding of difelikefalin or beta01.

(P.14, Lines 363-371)

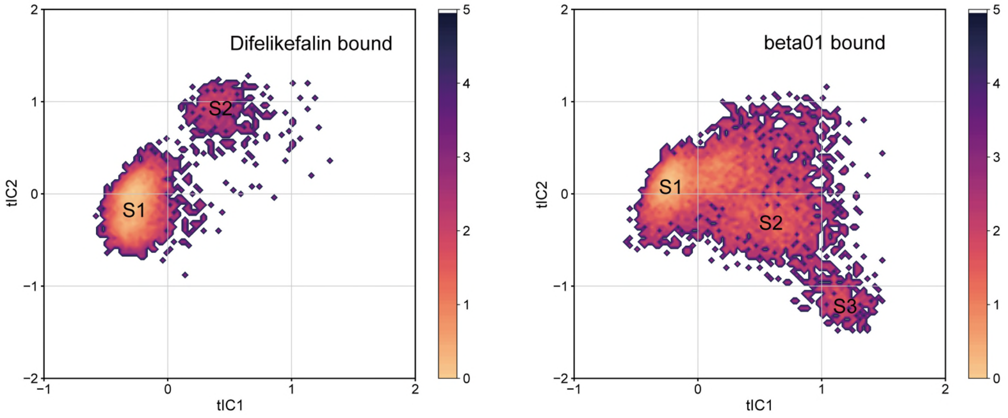

Fig. R6 (Fig. 4f-g in revised manuscript) | Free energy landscapes showing the conformational states of $L333^{7.56}$ in the KOR-difelikefalin (left) and KOR-beta01 (right) systems in MD. The color bar indicates the free energy values (in kcal/mol). These landscapes were generated from the two-dimensional tICA (time-lagged Independent Component Analysis) projections computed using the globally aligned heavy-atom coordinates of $L333^{7.56}$.

In the revised manuscript, we now state that "*the NMR and MD data offer a multi-timescale perspective on the conformational landscape of* $L333^{7.56}$, *demonstrating that* $L333^{7.56}$ *adopts distinct conformational states in the two ligand-bound systems.*". We

have also removed the previous direct comparison words and now present MD as complementary evidence supporting the presence of multiple conformational substates. (P.14, Lines 371-374)

Point 4: From the MD simulation it can be observed that the S2 rotameric state has a higher population and that the RMSD of L333 is greater in the beta01 state. This fits with the result from the structure that beta01 has a bigger cavity between helix 6 and 7 and that this leads to biased signaling. I am not sure if the NMR data really supports this hypothesis.

Response:

Thanks for this insightful suggestion.

We agree that the NMR data should NOT be interpreted as a direct reporter of the specific χ_2 rotameric states observed in MD simulations, which occur on much faster timescales.

The key contribution of the NMR experiment is distinct. It identifies a unique, slowly-exchanging conformational state of KOR that is exclusively stabilized by both beta01, and the reference biased agonist nalfurafine, but not by the balanced agonist difelikefalin or antagonist naltrexone. This state, manifested as a distinct resonance for the $M333^{7.56}$ probe, provides direct, solution-phase experimental evidence that biased ligands stabilize a receptor conformation distinct from canonical active or inactive states.

The expanded intracellular cavity observed by cryo-EM and its dynamic properties quantified by MD are entirely consistent with and provide a plausible structural basis for this uniquely stabilized conformational ensemble detected by NMR. Thus, the NMR data offer orthogonal validation of the central hypothesis that beta01 promotes a unique conformational landscape conducive to G protein signaling.

Suggested improvements

Point 5: In line 318 they should clearly state that they used the KORΔM mutant. And they should show how this mutant can recruit arrestin.

Response:

Thank you for your valuable suggestion.

We have revised the text at P.13, Line 342-345 to explicitly state: "... *we engineered a mutant by replacing all Met with Leu and generating a single Met probe at position 333 (named $KOR^{\Delta M}$-L333M)*". We apologize for the lack of clarity in the original phrasing.

Furthermore, we have performed an additional β-arrestin2 recruitment assay to address this concern. As depicted in **Fig.R5 (Supplementary Fig. 14b in revised manuscript)**, the results confirm that the $KOR^{\Delta M}$-L333M mutant retains its capacity to recruit β-arrestin2. (P.13, Line 342-345)

Point 6: The difference in the dynamic range of the MD and NMR results needs to be reanalyzed.

Response:

Thanks for your thoughtful comment regarding the dynamic range of the MD and NMR results.

We acknowledge the difference in the intrinsic timescales of MD simulations and NMR experiments, and agree that the two types of data should NOT be interpreted as directly equivalent.

In the revised manuscript, we currently show MD results as a valuable complementary evidence, supporting the observation that the conformational states of L333$^{7.56}$ differ between the KOR-difelikefalin and KOR-beta01 systems.

(P.14, Line 363-374)

Point 7: I think a more elegant way to use NMR would be to use the normal KOR and not the KORΔM receptor with 13C-Met labeling. A complete assignment would be difficult, but maybe just a difelikefalin, a beta01 and a naltrexone bound spectra could give insight into a different ratio of inactive, active and intermediate states. I am a great fan of NMR but somehow I feel without these ambiguous NMR results the overall statement of the paper would be more convincing.

Response:

We sincerely thank you for this valuable suggestion.

We fully agree that comparing ^{13}C -Met spectra of native KOR bound to difelikefalin, beta01 and naltrexone could, in principle, reveal population shifts among inactive, active and intermediate states without the need for complete assignment.

In practice, we first pursued exactly this way. We recorded ^{13}C-Met spectra of a construct retaining all nine endogenous methionines (M81$^{1.54}$, M90^{ICL1}, M112$^{2.57}$, M121$^{2.66}$, M142$^{3.36}$, M151$^{3.45}$, M152$^{3.46}$, M226$^{5.38}$, M249$^{5.61}$; named KOR-9M). As anticipated, the spectra were severely overlapped **(Fig. R4)**, preventing unambiguous detection of ligand-dependent changes at the functionally critical L333$^{7.56}$ position.

We are optimistic that future advances, such as higher-resolution NMR probes and high field NMR instrumentation, will overcome these challenges and enable comprehensive studies in fully wild-type receptors. For the purposes of this study, however, our site-specific strategy could provide the unambiguous data necessary to directly link ligand binding to distinct conformational states at a key functional interface.

Reviewer #3 (Remarks to the Author):

Zhang et al. combine in vitro assays, cryo-EM structures, and molecular simulations to investigate the structural foundations of G-protein bias at the κ-opioid receptor (KOR). Given that, despite some controversies, G-protein bias has been associated with favorable therapeutic outcomes for opioid receptor–targeting drugs, this study is timely, novel, and of clear scientific relevance.

However, before publication, I recommend that the authors address the following points:

Point 1: The authors present an alanine mutation at position Y7.43, emphasizing its role in bias modulation. To strengthen this conclusion, I recommend also generating Y7.43F and possibly Y7.43L mutants, as these substitutions maintain similar physicochemical properties and would help disentangle steric from polar effects.

Response:

Thank you for your insightful suggestion.

Following the reviewer's suggestion, we have performed functional assays for Y320$^{7.43}$F and Y320$^{7.43}$L mutants, and combined these data with the mechanistic insights from our difelikefalin analog beta01.

The Y320$^{7.43}$F mutation resulted in significantly impaired β-arrestin2 recruitment induced by difelikefalin, while Gi protein signaling potency was reduced 10 fold.

The Y320$^{7.43}$L mutation resulted in significant impairment in both G protein signaling and β-arrestin2 recruitment. This dual impairment may be attributed to steric hindrance effects arising from the Y320$^{7.43}$L substitution.

Collectively, these results further clarify the critical role of Y320$^{7.43}$ in regulating both G protein and β-arrestin2 signaling pathways.

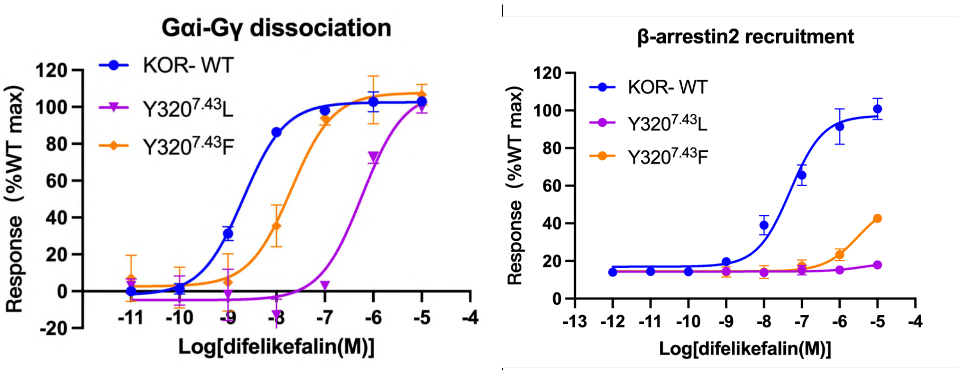

Fig. R7 | Effects of Y320$^{7.43}$L and Y320$^{7.43}$F of KOR on G-protein signaling (left) and β-arrestin2 recruitment (right) in response to difelikefalin.

Point 2: The MD trajectories are not currently available in any repository. I would like to review these data before final acceptance. Moreover, the authors should deposit the MD simulations in a public repository (e.g., Zenodo, OSF, or GPCRmd) to ensure

reproducibility and transparency. How was the protonation of tiratable residues assigned for MD simulations?

Response:

Thanks for emphasizing the importance of data transparency and reproducibility.

We have organized and compressed the MD trajectories and associated input files, and have deposited these datasets in the public repository Zenodo. The data are now publicly accessible via the following link: https://zenodo.org/records/17984759.

Regarding the protonation states of titratable residues, the protonation assignments were determined using PROPKA 3.1 at physiological pH (pH 7.0). All histidine residues were visually inspected, and their protonation states (HIS/HIE/HID/HIP) were assigned based on local hydrogen-bonding environments and known structural context within KOR. The final protonation states were validated before system minimization and equilibration. This entire procedure has been clearly stated in revised methods.

(P.29, Line 850-855)

Point 3: I recommend performing MD simulations of the Y7.43A mutant in complex with both ligands, followed by the same analyses used for the wild-type receptor—particularly monitoring the TM7–TM6 distance and other molecular features highlighted in the manuscript.

Response:

Thank you for your insightful suggestion!

Following your recommendation, we performed MD simulations of the KOR$^{Y7.43A}$ mutant in complex with both difelikefalin and beta01, and analyzed the TM6-TM7 distance as well as the SASA of the G protein binding pocket residues using the same procedures applied to the wild-type receptor.

(P.13, Line 326-332)

As displayed in the **Fig. R8,** our results show that, for both ligands, the Y$^{7.43}$A mutation leads to an increase in the TM6-TM7 distance compared with the KOR^{WT}-difelikefalin system (KOR^{WT}-difelikefalin: 9.35 ± 1.74 Å, KOR$^{Y7.43A}$-difelikefalin: 11.99 ± 2.45 Å, KOR$^{Y7.43A}$-beta01: 11.12 ± 1.56 Å). Consistently, the SASA of the G protein pocket residues is also enlarged in the mutant systems (KOR^{WT}-difelikefalin: 1738 ± 123 Å^2, KOR$^{Y7.43A}$-difelikefalin: 1767 ± 155 Å^2, KOR$^{Y7.43A}$-beta01: 1764 ± 145 Å^2). These observations aligned well with our experimental findings, indicating that the Y$^{7.43}$A mutation promotes a more open intracellular pocket and shifts the receptor toward preferential activation of G-protein signaling.

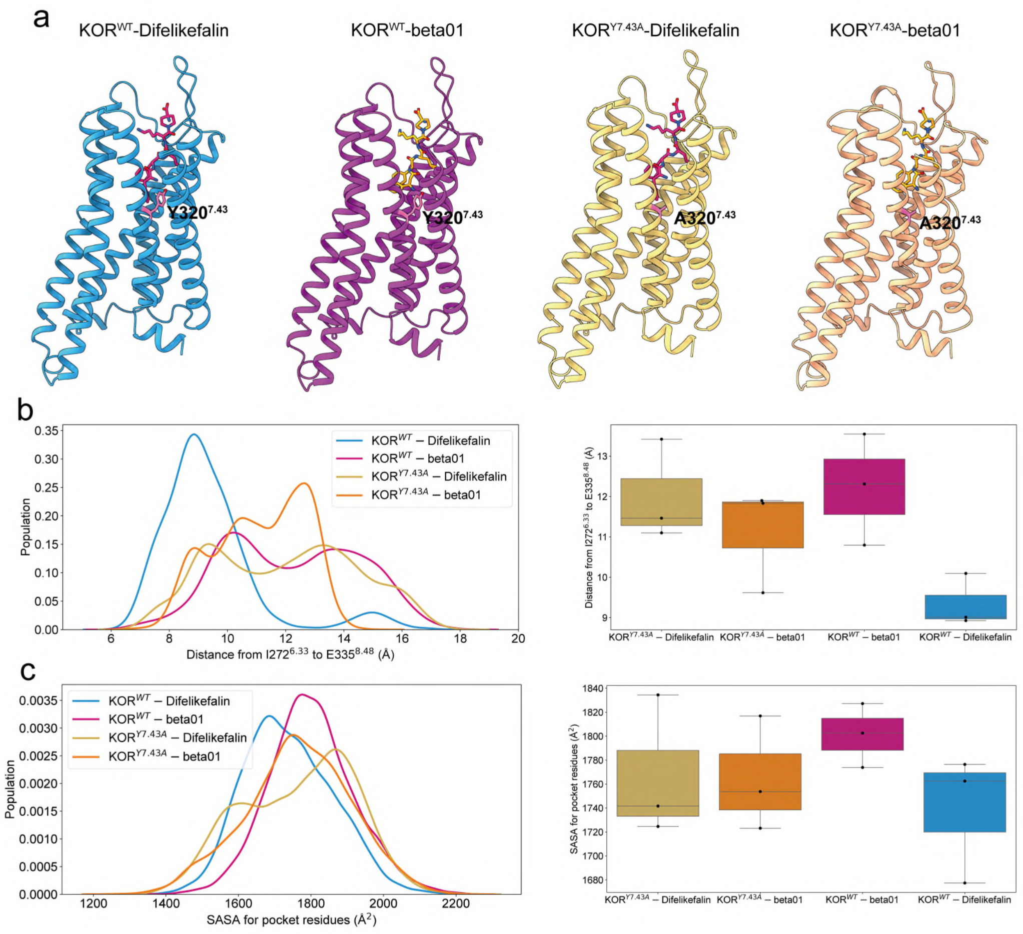

Fig. R8 (Supplementary Fig. 13 in revised manuscript) | Conformational alterations for the intracellular domain of KOR^{WT} and KOR$^{Y7.43A}$ induced by difelikefalin and beta01 in MD.

a, The initial structures of simulation systems KOR^{WT}-difelikefalin, KOR^{WT}-beta01, KOR$^{Y7.43A}$-difelikefalin and KOR$^{Y7.43A}$-beta01. **b,** the distribution of the Cα distance between I272$^{6.33}$ and E335$^{8.48}$ for the KOR^{WT}-difelikefalin, KOR^{WT}-beta01, KOR$^{Y7.43A}$-difelikefalin and KOR$^{Y7.43A}$-beta01 systems in MD, illustrated using a KDE plot (left) to show the probability density and a boxplot (right) to summarize statistical variation between the two systems. **c,** the distribution of SASA values for pocket residues in the KOR^{WT}-difelikefalin, KOR^{WT}-beta01, KOR$^{Y7.43A}$-difelikefalin and KOR$^{Y7.43A}$-beta01 systems in MD, illustrated using a KDE plot (left) and a boxplot (right).

Point 4: In Fig 4, I suggest including a supplementary panel showing conformational clustering of the L333 residue. This would help visualize the local conformational diversity and support the interpretations drawn from the main text.

Response:

Thank you for your insightful suggestion!

In accordance with your recommendation, we performed tICA-based conformational clustering of residue L333$^{7.56}$, and the results is provided in revised **Fig. 4**. As shown

below, the difelikefalin system exhibits two distinct conformational clusters (S1 and S2), whereas the beta01 system displays three well-defined clusters (S1, S2 and S3), highlighting the greater local conformational diversity of L333$^{7.56}$ in the latter. Such result corresponds with NMR results in a different timescale and confirm the conformational heterogeneity induced by different ligands.

(P.14, Line 363-374)

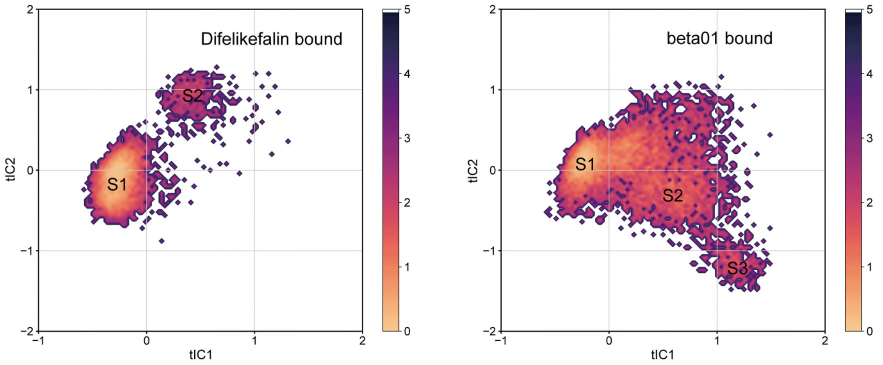

Fig. R6 (Fig. 4f-g in revised manuscript) | Free energy landscapes showing the conformational states of L333$^{7.56}$ in the KOR-difelikefalin (left) and KOR-beta01 (right) systems in MD. The color bar indicates the free energy values (in kcal/mol). These landscapes were generated from the 2D tICA projections computed using the globally aligned heavy-atom coordinates of L333$^{7.56}$.

Point 5: Did the authors attempt to back-calculate NMR spectra from their MD simulations? Comparing simulated and experimental spectra (if available) could provide valuable validation of the proposed conformational states.

Response:

We thank the reviewer for this valuable suggestion.

Following this recommendation, we attempted to back-calculate NMR chemical shifts from our MD simulations using the SHIFTX2 software, which employs machine learning approaches to predict NMR chemical shifts based on structural inputs.

Specifically, we extracted structures from the full trajectories of the KOR-difelikefalin and KOR-beta01 systems and calculated the CG/CD1/CD2–HG two-dimensional chemical shifts. The reconstructed spectra are shown in **Fig. R9**.

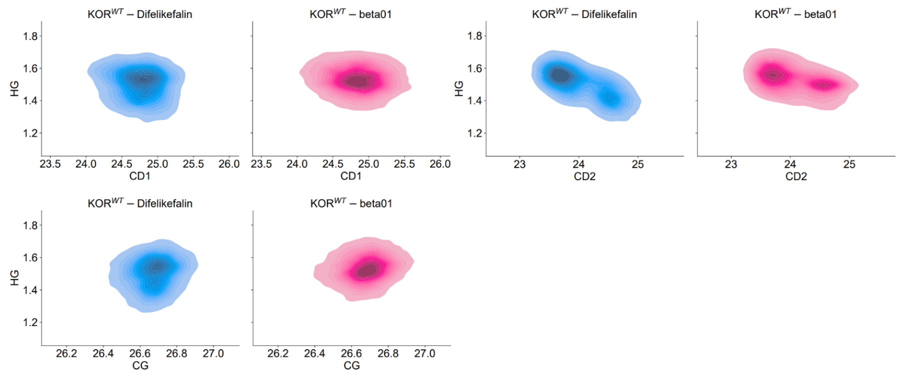

Fig. R9 | Reconstructed 2D chemical shift from MD simulations.

Reviewer #4 (Remarks to the Author):

Overall, this is clean and well written manuscript that details peripherally restricted KOR agonist Korsuva used in the treatment of pruritus. The authors interrogate mutations surrounding the orthosteric binding pocket and identify a modified Korsuva molecule (b01) that exhibits G protein over bArrestin bias. Structures with this new molecule show atomic details of enhanced binding. The authors follow up on this new compound in mice models showing “adverse” effects with this new biased molecule are mitigated, presumably through its greater G-protein bias. Additionally, NMR studies find along with MD studies that suggest this new compound appears to stabilizes an intermediate intracellular receptor conformation, that perhaps appears to modify the intracellular cavity. To improve transparency, the authors are encouraged to acknowledge data limitations, provide additional panels demonstrating map quality in critical regions, and supplement histogram plots with box and whisker overlays.

Major:

Point 1: The comparison between b01 and difelikefalin is awkward due to different construct preps (LgBit fusion for b01). The authors must explicitly address this in the main text especially when discussing intracellular cavity differences.

Response:

We thank the reviewer for this kind suggestion.

We fully appreciate the concern that different constructs may introduce systematic bias.

To address this directly, we presented key experimental evidence to validate the structural and functional integrity of our constructs.

(1) Direct structural comparison evidence.
During the early stages of the project, we determined the structures of the Gi complex for difelikefalin bound to KOR (without LgBiT) and difelikefalin bound to KOR-LgBiT. This experimental detail has been added to the revised Methods section. As shown in the **Fig. R10** below, the superposition of these two structures reveals

that their transmembrane core, orthosteric binding pocket, and, crucially, the conformation of the intracellular regions (including TM6, TM7, and Helix8) are nearly identical. This provides direct experimental proof that C-terminal fusion of the LgBiT tag does not significantly alter the overall structure of KOR or the conformation of its intracellular cavity.

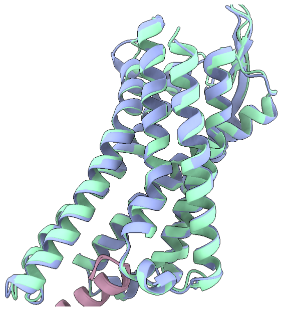

difelikefalin-KOR (no LgBiT)-Gi / difelikefalin-KOR-LgBiT-Gi

Fig. R10 | Structure comparison of difelikefalin bound KOR-Gi and KOR-LgBiT-Gi

(2) Functional validation

Furthermore, as shown in **Fig. R11**, the KOR-LgBiT fusion protein exhibits virtually indistinguishable functional properties in Glosensor assay compared to the wild-type KOR. This functionally confirms that the introduction of the LgBiT does not compromise the receptor's signaling competence, thereby indirectly supporting its structural integrity.

Based on the above validation, we are confident in the reliability of the LgBiT-containing construct. For this manuscript, we ultimately selected the difelikefalin-KOR (LgBiT-less) structure for presentation and comparison for the sole reason that this dataset achieved a higher final resolution (2.4 Å), which is beneficial for finer interaction analysis.

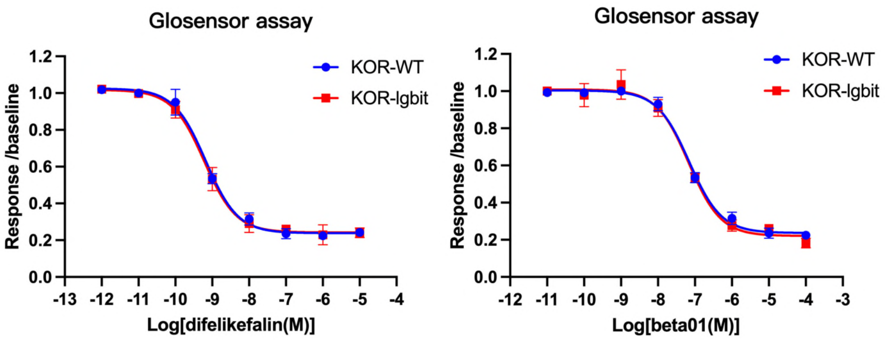

Fig. R11 | Glosensor assay for KOR and KOR-LgBit activated by difelikefalin and beta01

Point 2: The larger intracellular cavity (fig 3h and page 11, line 280) is small, especially (1) given the often-poorer density of TM7/TM8 and (2) the beta01 bound complex employed a LgBit (presumably to this H8) while the difelikefalin did not. I would again

strongly advise specifically addressing this in the results, temper the observations with this caveat and state the rationale to perform additional MD and NMR to investigate these observations using orthogonal methodologies.

Response:

We thank the reviewer for this valuable suggestion.

(1) Regarding local density and resolution: We acknowledge that the cryo-EM density for intracellular loops and helix ends can be challenging. In response to below Point 7 suggestion (and as shown in **Fig. R13**), we provide a side-by-side view of the local density for the TM7-H8 region, colored by local resolution. This visualization demonstrates that, while the resolution is lower in these peripheral regions compared to the transmembrane core, the density is sufficiently defined to model the backbone and key side chains reliably for comparative analysis. The observed outward shift of Helix8 in the beta01 structure is supported by continuous density.

(2) Regarding the LgBiT fusion construct: As detailed in our response to Reviewer #4 (Point 1), we have previously determined the structure of the difelikefalin-bound KOR-Gi complex using an identical LgBiT-fused construct. Superposition of this structure with the one presented in the manuscript shows negligible differences in the transmembrane core and the intracellular conformation of TM6, TM7, and H8. This confirms that the LgBiT tag itself does not dictate the conformation of the intracellular cavity (**Fig. R10**). Furthermore, functional assays confirm that the LgBiT fusion does not alter receptor signaling properties (**Fig. R11**).

In addition, following your suggestions, MD simulations based on the same KOR setting were performed and the results met our hypothesis (**Fig. R3, R8 and R12**).

Point 3: The MD RMSD fluctuations appear within error and are complicated by the different construct preps. That is, starting models could bias MD observations. Perhaps, if the authors wish to be more thorough starting with essentially the same atomic model with the two different ligands bound would help make this data more compelling (or less).

Response:

Thanks for your constructive suggestion.

To address the concern regarding potential bias from different starting models, we reconstructed the simulation systems using the same initial KOR conformation for both ligands. The beta01 ligand was aligned to the binding pocket of difelikefalin, and 500-ns MD simulations were performed for each system with three independent replicates.

As shown in **Fig. R12**, compared with the KOR-difelikefalin system, the KOR-beta01 system exhibits a pronounced increase in the TM6-TM7 distance, accompanied by a significant enlargement of the SASA of G protein binding pocket residues. These results provide evidence that beta01 promotes widening of the intracellular G protein pocket,

thereby favoring G protein signaling over β-arrestin signaling, consistent with our experimental observations.

Regarding the RMSD of the intracellular segment of TM7 heavy atoms, we observed that the KOR-beta01 system shows a higher RMSD compared with the KOR-difelikefalin system. A plausible explanation is that difelikefalin can form a π–π interaction with KOR $Y^{7.43}$, whereas beta01 cannot, potentially leading to increased flexibility and higher RMSD in the TM7 intracellular segment in the beta01-bound system.

(P.12, Line 293-312)

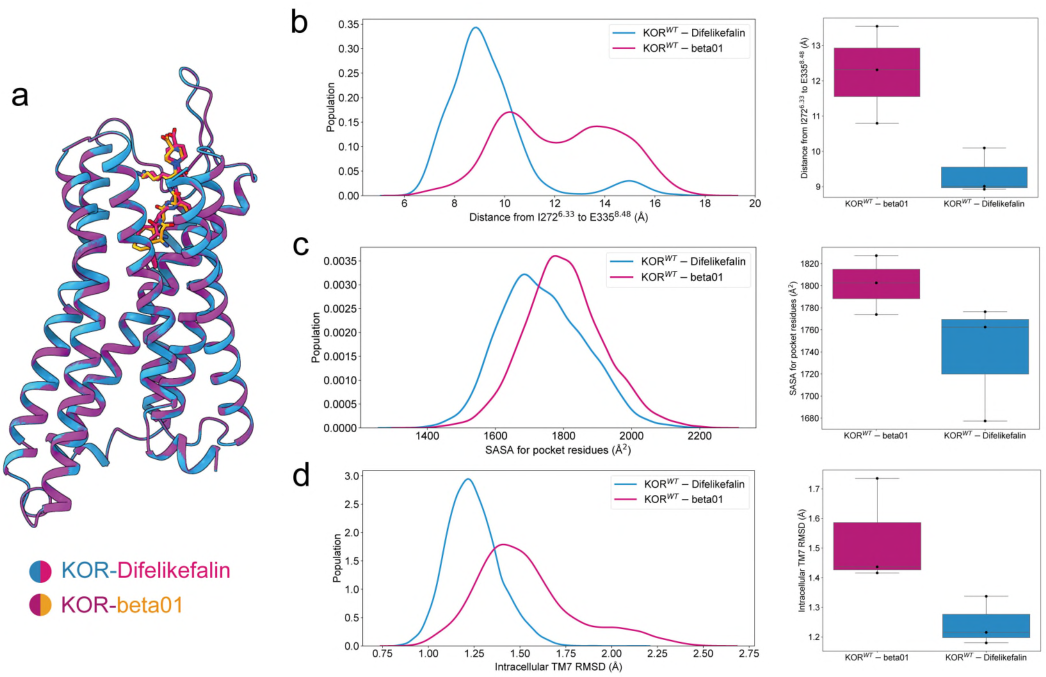

Fig. R12 | (Supplementary Fig. 11b, d and f in revised manuscript) Conformational alterations for the intracellular domain of KOR induced by difelikefalin and beta01 in molecular dynamics. a, The initial structures of simulation systems KOR-Difelikefalin and KOR-beta01. **b,** the distribution of the Cα distance between I272$^{6.33}$ and E335$^{8.48}$ for the KOR–difelikefalin and KOR-beta01 systems in MD, illustrated using a KDE plot (left) to show the probability density and a boxplot (right) to summarize statistical variation between the two systems. **c,** the distribution of SASA values for pocket residues in the KOR-difelikefalin and KOR-beta01 systems in MD, illustrated using a KDE plot (left) and a boxplot (right). **d,** the distribution of heavy-atom RMSD for intracellular TM7 in the KOR–difelikefalin and KOR-beta01 systems in MD, illustrated using a KDE plot (left) and a boxplot (right).

Point 4: In regards to above please clarify methods for MD simulations to address this.

Response:

Thanks for your valuable suggestion.

We have revised and expanded the Methods section to provide a more detailed description of the MD simulations. In particular, we have clarified that the initial conformations of the difelikefalin and beta01 systems were identical, ensuring that any observed differences in the simulations arise from the ligands rather than starting model bias.

(P.29, Line 838-847)

Point 5: The RMSD fluctuations page 12 line 302-307 seem to be within error; perhaps a movie would be more beneficial. Fig 4g and Fig 10 (and all other MD histograms) must be co-plotted with the current histogram in addition to an overlay of a box and whisker plot.

Response:

Thanks for your suggestion.

To better illustrate the dynamic differences underlying the RMSD fluctuations, we have added Supplementary Video 1, which visualizes TM7 movements in the KOR-difelikefalin and KOR-β01 systems from our new simulations. As shown in the video, the KOR-difelikefalin complex (cyan) exhibits an inward movement of TM7, whereas the KOR-beta01 complex (magenta) shows an outward displacement of TM7, resulting in a larger intracellular pocket. These conformational changes provide a more intuitive and direct representation of the RMSD differences observed in the simulations.

(P.12, Line 312-315)

In addition, in response to the reviewer's comment regarding data visualization, we have updated all MD-related histograms by co-plotting box-and-whisker plots alongside the existing histograms. This combined presentation enables clearer visualization of the distributions, central tendencies, and variability of the measured parameters, thereby facilitating more straightforward comparisons across different systems. The revised plots are provided in **Supplementary Figs. 11–13.**

Point 6: Much of the difference between the two models appears to be around the TM7/8 region with respect to the larger cavity size. If the authors wish to demonstrate these differences an additional main Fig panel showing density of TM7/TM8 in a side-by-side view for each KOR-ligand complex with transparent local resolution mapped to the surface of these two maps/models is critical for reader evaluation. This would be helpful to illustrate key structural differences and local resolution and map quality for this region.

Response:

We thank the reviewer for this valuable suggestion. We have generated a new Fig panel to provide a side-by-side view of the cryo-EM density maps for the TM7-Helix8 region in the difelikefalin- and beta01-bound KOR–Gi complexes, with the local resolution mapped as a transparent color surface onto the maps, and the corresponding atomic models overlaid.

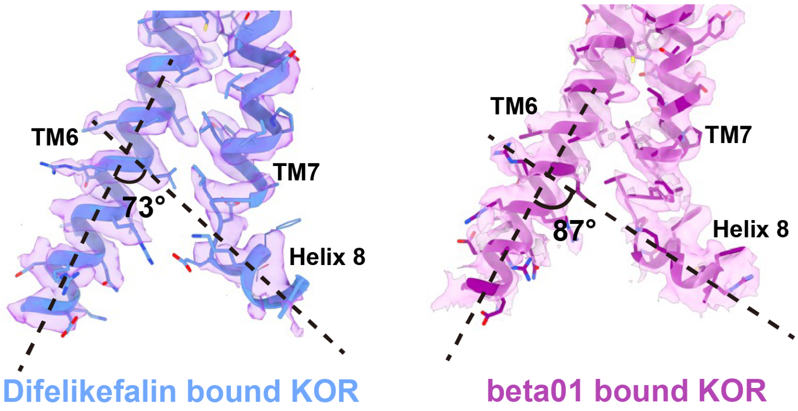

Fig. R13 | Cryo-EM density maps of the intracellular portions of TM6, TM7, and Helix8 of KOR, in the presence of difelikefalin and beta01.

This Figure has been added in **Supplementary Fig. 10** in the revised manuscript.

(P.11, Line 283)

Point 7: Comparing the two models derived from the cryo-EM maps the intracellular cavity difference is subtle the numbers on page 12 line 296-297 show a small difference within error. Given the difference in the two preparations this does cause some concern. Although, the NMR experiments help assuage these concerns.

Response:

We thank the reviewer for this important perspective.

We fully appreciate the caution required when evaluating subtle conformational differences from single cryo-EM structures derived from different preparations. However, the robustness of our conclusion stems not from the structural comparison alone, but from convergent evidence provided by three orthogonal methods: cryo-EM, MD, and NMR.

Specifically: (1) Cryo-EM provided the initial structural clue. (2) MD simulations (performed in identical, tag-free systems) independently validated this trend with quantitative data, showing statistically significant differences in metrics like the TM6-Helix8 distance and cavity volume. (3) NMR ultimately provided direct experimental evidence in solution for a unique conformational ensemble in this region.

Thus, we believe the cross-validation across multiple methodologies robustly supports the conclusion that beta01 induces a conformational change in the intracellular cavity.

Point 8: Please address the extra density between beta01's N-terminal phenylalanine and Y320. Are multiple binding poses congruent with the ensemble map density. How much different would the beta01 pose vs difelikefalin be line with this alternative pose? How would that alter Y320? Is the Y320 in beta01 strong enough to make claims about relative changes in side chain position, the density seems poor for this residue for the beta01 map.

Response:

We thank the Reviewer for this careful observation. In our initial KOR–beta01 reconstruction, we indeed observed a weak, elongated density feature between the N-terminal phenylalanine of beta01 and Y320$^{7.43}$ (**Fig. R14, left**), raising the possibility of local heterogeneity that could affect interpretation of the ligand pose and Y320$^{7.43}$.

To address this, we reprocessed the beta01 dataset with a binding-pocket–focused local refinement using a mask centered on the orthosteric site. This yielded an updated map with improved density definition in the ligand-binding pocket (**Fig. R14, right**). In the refined map, the previously observed “continuum” density is substantially reduced and the density supports a single dominant beta01 pose; we do not observe features that require an alternative binding pose. Moreover, alternative placements that would bring the N-terminal phenylalanine into a stable direct contact with Y320$^{7.43}$ are not supported by the refined density and worsen the fit elsewhere in the ligand.

Consistent with this, the refined map indicates no evidence for a persistent direct interaction between the beta01 N-terminal phenylalanine and Y320$^{7.43}$. We have also revised the manuscript to avoid over-interpreting subtle Y320$^{7.43}$ side-chain differences beyond what is supported by the local map quality. All relevant figures and text have been updated accordingly.

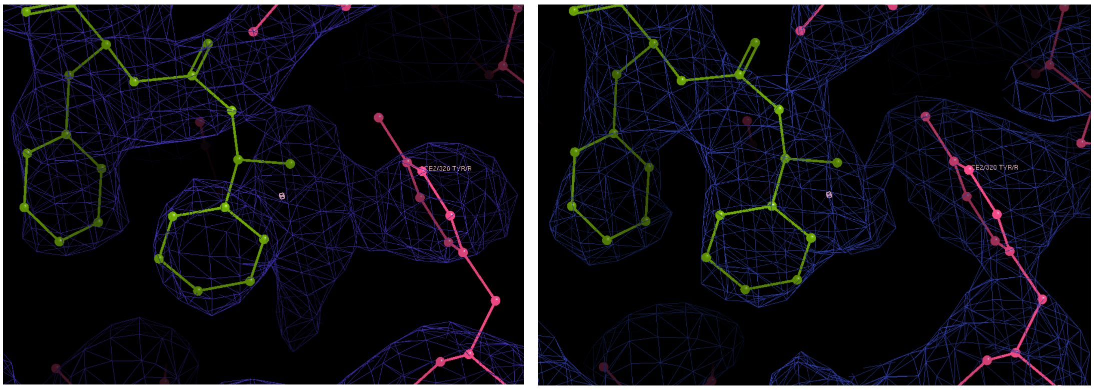

Fig. R14 | Local view of the electron density between the N-terminus of beta01 and Y320$^{7.43}$.
The left panel shows the initial map, and the right panel shows the map after re-processing and refinement. Beta01 is shown in green, and KOR is shown in red.

Point 9: How do mutations around beta01 differential pocket affect binding. For example Y320A? Are these results consistent with two binding poses for beta01, given the unaccounted for density?
Response:

We sincerely thank the reviewer for this insightful question, which prompted us to conduct the experiment of testing beta01 on the Y320A mutant. The new functional data reveal that in the Y320A mutant, beta01's potency for G protein activation decreases by nearly 1000-fold, and its already minimal β-arrestin2 recruitment is completely abolished **(Fig. R15)**.

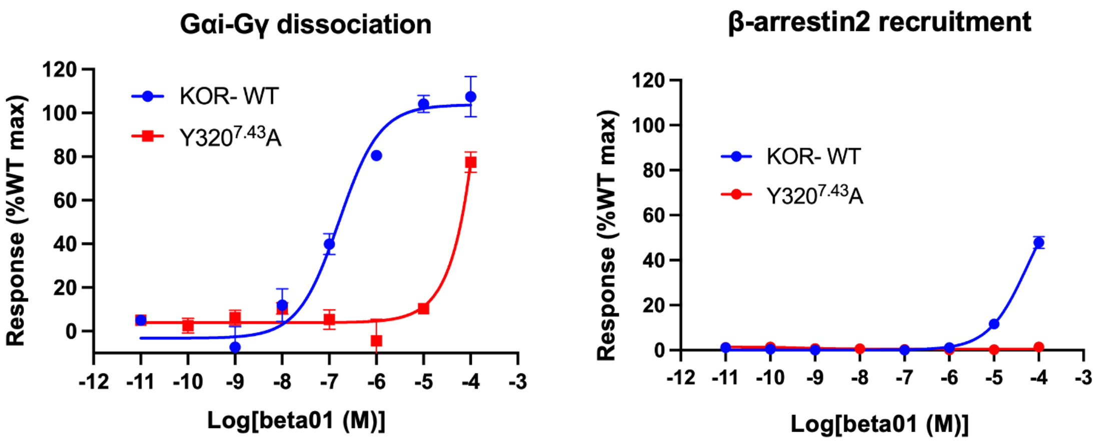

Fig. R15 | Effects of Y320$^{7.43}$A of KOR on G-protein signaling (left) and β-arrestin2 recruitment (right) in response to beta01.

These results are consistent with our structural observations and reveals a switch in the interaction mode with Y320$^{7.43}$.

(1) For the balanced agonist difelikefalin, the primary interaction with Y320$^{7.43}$ is a strong π-π stacking between its N-terminal phenyl ring and the tyrosine's aromatic ring. This specific geometry is highly effective at promoting the conformational changes that lead to robust β-arrestin recruitment.

(2) For the biased agonist beta01, our cryo-EM structure shows a fundamental shift. The interaction with Y320$^{7.43}$ transitions from strong π-π stacking to a reinforced set of hydrogen bonds involving the backbone of its N-terminal β-phenylalanine. Crucially, the phenolic hydroxyl group of Y320$^{7.43}$ is integral to this new hydrogen-bonding network.

The switch from a π-π-driven to a H-bond-driven interaction with Y320 alters the strain and micro-switches activated within the receptor, disfavoring the conformational ensemble that recruits β-arrestin2.

The new data show that Y320$^{7.43}$ remains critically important for beta01 not as a passive scaffold, but as an active participant in a new, polar interaction network. The Y320A mutation destroys this network by removing the essential hydroxyl group, explaining the catastrophic loss in beta01's G protein potency. The aromatic ring of Y320$^{7.43}$ is likely still important for orienting the residue to form these specific H-bonds.

Minor:

Point 10: Fig 5 “sodium binding site is odd” since this is in part between 2.50 and other residues in the inactive state. 4N6H of DOR shows interaction of sodium with 3.35,3.39,6.48 in their 1.8A inactive state structure. Perhaps putative sodium coordinating residue would be more appropriate language or omit.

Response:

We thank the reviewer for this expert comment.

We agree that in the active-state KOR-Gi complex, this site differs from the canonical sodium pocket conformation. Following the suggestion, we have revised the terminology to "putative sodium binding site" in **Fig. 5** and the main text to more accurately reflect the interpretive nature of this assignment.

Point 11: Local resolution map to the surface of the ligand in Extended Data Fig 1 and 8 would be helpful

Response:

We thank the Reviewer for this insightful suggestion.

In the revised manuscript, we have now added local resolution colored transparent maps over the ligand densities to allow direct visualization of the quality of the difelikefalin and beta01. The improved visualization clearly shows that both ligands are supported by high-quality density (2.5-2.7 Å for difelikefalin and 2.9-3.1 Å for beta01), thereby enabling reliable model interpretation **(Fig. R16)**. These updated local-resolution presentations are included in the new Supplementary Fig. 1d and Supplementary Fig. 8d.

(P.6, Line 133 and P.10, Line 260)

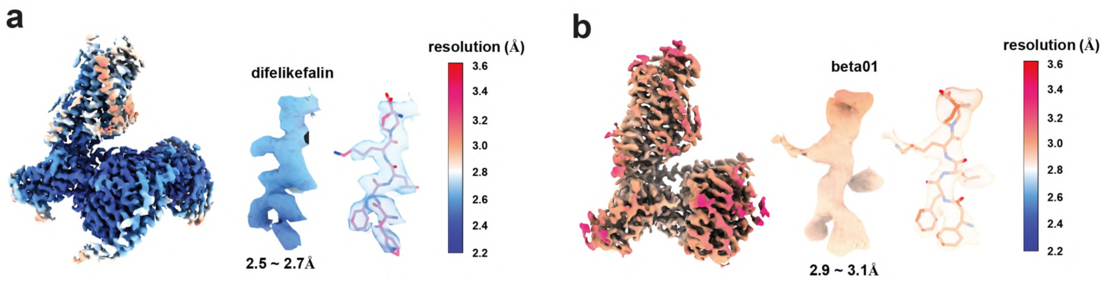

Fig. R16 (Supplementary Fig. 1d and Supplementary Fig. 8d in revised manuscript) Local resolution of the difelikefalin- and beta01–bound KOR–Gi complexes.

a, Local-resolution map of the difelikefalin-KOR-Gi complex, shown with the surface of the ligand and a transparent local-resolution-colored surface fitted to the model. The ligand region is well resolved (2.5-2.7 Å), supporting accurate model interpretation.

b, Local-resolution map of the beta01-KOR-Gi complex, displayed using the same unified color scale. The beta01 density is clearly visualized at 2.9-3.1 Å, enabling confident placement of the ligand. Note: Color bar indicates local resolution in Å.

Point 12: The heat map key for local resolution lacks units and coloring between 1 and 8 is different, which is odd given the high quality and uniformity in the rest of the manuscript.

Response:

We thank the Reviewer for this insightful suggestion.

The unit (Å) of the heat map key has been added and the coloring of two maps has been unified **(Supplementary Fig. 1d and Supplementary Fig. 8d)**.

(P.6, Line 133 and P.10, Line 260)

Point 13: The “dust” could be omitted from 1d to aid in visualization of pertinent density.
Response:

We thank the Reviewer for this insightful suggestion.

We have omitted the "dust" from **Supplementary Fig. 1d** and updated the optimized Fig as new **Supplementary Fig. 1d** in the revised manuscript.

Point 14: Statistics lacking on data tables.
Response:

We thank the reviewer for this suggestion.

We have performed a comprehensive check and revision of all data tables throughout the manuscript. All cryo-EM statistics and cellular functional data are provided in the tables. Raw data points from the in vivo behavioral studies are available in the Source Data file. Furthermore, the cryo-EM structures determined in this study have been deposited in the Protein Data Bank (PDB). The corresponding accession numbers and data availability details are provided on page 31, lines 914-918 of the revised manuscript.
